# The Pursuit of Shortwave Infrared-Emitting Nanoparticles with Bright Fluorescence through Molecular Design and Excited-State Engineering of Molecular Aggregates

**DOI:** 10.1021/acsnanoscienceau.1c00038

**Published:** 2022-02-21

**Authors:** Hubert Piwoński, Shuho Nozue, Satoshi Habuchi

**Affiliations:** Biological and Environmental Science and Engineering Division, King Abdullah University of Science and Technology, Thuwal 23955-6900, Saudi Arabia

**Keywords:** shortwave infrared, Pdots, J-aggregates, fluorescence microscopy, single particle, time-gated imaging

## Abstract

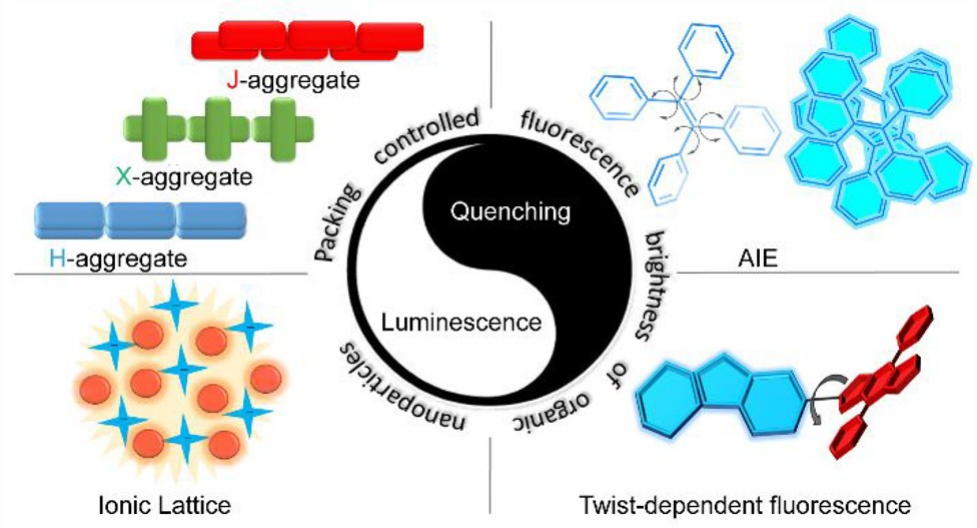

Shortwave infrared (SWIR) fluorescence detection gradually becomes a pivotal real-time imaging modality, allowing one to elucidate biological complexity in deep tissues with subcellular resolution. The key challenge for the further growth of this imaging modality is the design of new brighter biocompatible fluorescent probes. This review summarizes the recent progress in the development of organic-based nanomaterials with an emphasis on new strategies that extend the fluorescence wavelength from the near-infrared to the SWIR spectral range and amplify the fluorescence brightness. We first introduce the most representative molecular design strategies to obtain near-infrared-SWIR wavelength fluorescence emission from small organic molecules. We then discuss how the formation of nanoparticles based on small organic molecules contributes to the improvement of fluorescence brightness and the shift of fluorescence to SWIR, with a special emphasis on the excited-state engineering of molecular probes in an aggregate state and spatial packing of the molecules in nanoparticles. We build our discussion based on a historical perspective on the photophysics of molecular aggregates. We extend this discussion to nanoparticles made of conjugated polymers and discuss how fluorescence characteristics could be improved by molecular design and chain conformation of the polymer molecules in nanoparticles. We conclude the article with future directions necessary to expand this imaging modality to wider bioimaging applications including single-particle deep tissue imaging. Issues related to the characterization of SWIR fluorophores, including fluorescence quantum yield unification, are also mentioned.

## 1 Introduction

Fluorescence microscopy has been developing faster than any other imaging modality. Through recent innovations of fluorescence imaging techniques, including laser scanning confocal, multiphoton, light sheet, and superresolution microscopy, it has been realized that their applicability and future improvements depend heavily on the development of new fluorochromes used for labeling the targeted structures. The development of fluorescent probes for tissue and whole organism imaging becomes even more important because visualizing and characterizing spatiotemporal dynamics of cells in the context of a whole organism is the most definitive way to understand their functions and physiology, together with understanding the pathophysiology of diseases.

The general requirements for ideal fluorochromes are a large fluorescence quantum yield (Φ_fl_, QY), large molar extinction coefficient (ε), large two-photon absorption cross section (δ), narrow spectral bandwidths, high photostability under irradiation, water solubility, and small size. In addition, the tissue and whole-organism imaging often requires the characterization of spatiotemporal dynamics of biological molecules and structures at millimeter to centimeter depth with micrometer resolution. The fluorescence imaging of a large biological specimen in the visible wavelength region (400–650 nm) faces several issues. First, biological tissues show a large absorption of visible light. This causes (1) a large autofluorescence from the specimens and (2) a significant attenuation of the excitation light and fluorescence emission. Second, biological tissues exhibit a large scattering of light in the visible wavelength region. This prevents us from obtaining sharp and bright fluorescence images from the tissue specimens. These issues are less pronounced in the near-infrared (NIR, 650–950 nm, a synonym for the term near-infrared I (NIR-I)) spectral region. In the shortwave infrared (SWIR, 1000–1700 nm, a synonym for the term near-infrared II (NIR-II)) spectral region, the absorption and scattering of light by biological tissues are even more suppressed, and thus this wavelength region has been explored as new spectral window for bioimaging applications.

A wide range of innovative fluorescent probes that emit photoluminescence in the SWIR spectral window have been developed during the past decade. While both inorganic (e.g., semiconductor quantum dots (QDs), rare earth-doped nanoparticles (NPs)) and organic fluorophores have been developed, in this review, we focus only on organic-based fluorescence probes. Inorganic probes, in particular, QDs, often exhibit a relatively large Φ_fl_ compared with organic probes, inherent structural properties of inorganic materials including chemical composition (e.g., presence of heavy elements), surface chemistry (e.g., functionalization with amines versus carboxylic acids), size (that affects clearance pathway, preferred <5.5 nm for renal clearance), and shape (that affects biodistribution, nonspecific uptake in the liver and spleen) have a certain effect on toxicity and explicit pharmacokinetics. Several studies on inorganic semiconductor-based luminophores indicated a pharmacokinetics or acute or chronic toxicity of these probes in vivo.^1−8^ The progress of the development of SWIR emitting probes based on inorganic luminophores can be found in other excellent review articles.^9−11^ Organic probes (e.g., small-molecule dyes, polymers, and organic nanoparticles) have been used for a wide range of applications, including drug delivery systems,^12^ antiviral therapeutics,^13^ photoactivatable enzymes,^14^ enzyme monitoring systems,^15,16^ neurotransmitter sensing,^17^ cancer phototherapeutics,^18^ glucose level monitoring systems,^19^ imaging of intracellular traffics,^20^ etc. Their applicability often goes beyond the scope of bioimaging. They have been used for latent fingerprint detection,^21^ chemical sensing,^22−24^ photocatalytic hydrogen generation,^25^ and so on. Many articles indeed mentioned that organic fluorophores have better biocompatibility than inorganic probes,^26^ which promotes their applications in bioimaging and related fields.

This review outlines progress in the development of NIR/SWIR emitting organic-based fluorescence probes, starting from a basic design strategy for obtaining small organic molecules and polymers that have been utilized as basic building blocks of fluorescent probes based on molecular aggregates and nanoparticles. We then introduce strategies to improve the fluorescence brightness of aggregates and nanoparticles based on both small molecules and polymers. This includes excited-state engineering in molecular aggregates (e.g., aggregation-induced conformation change and rigidification, formation of J-aggregates) and preservation of fluorescence characteristics in molecular aggregates (e.g., molecular spacers, side chains, and twist conformation). While most of the previous review articles on SWIR fluorophores focused on their imaging applications,^27^ in this review article, we discuss fluorescence properties (e.g., fluorescence brightness and wavelength) of SWIR fluorophores from a photophysics perspective, which provides a guideline for the rational design of new organic SWIR fluorescent probes. In addition, we introduce imaging applications of SWIR fluorophores with particular emphasis on single-particle imaging, which would be one of the important future directions of the SWIR fluorescence imaging. We conclude this review with a future outlook that discusses potential solutions for key challenges that we need to overcome for the future development of SWIR probes. This review is intended for both an expert and a graduate student who work on the development of new SWIR probes from a photophysics standpoint, in particular, those who work on SWIR probes based on molecular aggregates and nanoparticles.

## 2 Small-Organic-Molecule Fluorescent Probes

In recent years, there is a visible transition in the direction of life science research, from the study on simplified model processes occurring in an isolated environment (e.g., cultured cells) to the study on more complicated biological processes occurring at a larger scale in order to understand functions and physiology of cells at the level of tissues and the whole organism. Therefore, rational engineering of molecular structures is a prerequisite to yield biocompatible optical nanomaterials applicable for deep-tissue imaging, which should possess absorption and fluorescence spectra covering the NIR/SWIR spectral region. This is extremely essential as we shift from a cozy visible to a darker NIR/SWIR spectral zone. Since the energy gap between an electronic ground and excited state determines absorption and fluorescence wavelengths of organic fluorophores, designing molecules with a low energy gap is a key to shift their spectra to the NIR/SWIR spectral range. Currently, small organic molecules are most basic building blocks of fluorescent probes utilized at the forefront of applications in state-of-the-art fluorescence imaging techniques. In addition to their imaging application as a molecular probe, they have also been used as building blocks of fluorescent probes based on molecular aggregates and nanoparticles. In this section, we briefly introduce two types of NIR/SWIR emitting small organic molecules, polymethine and donor–acceptor dyes, which have most frequently been used as the building block for molecular aggregates and nanoparticle probes.

### 2.1 Polymethine Dyes

**Figure 1:**
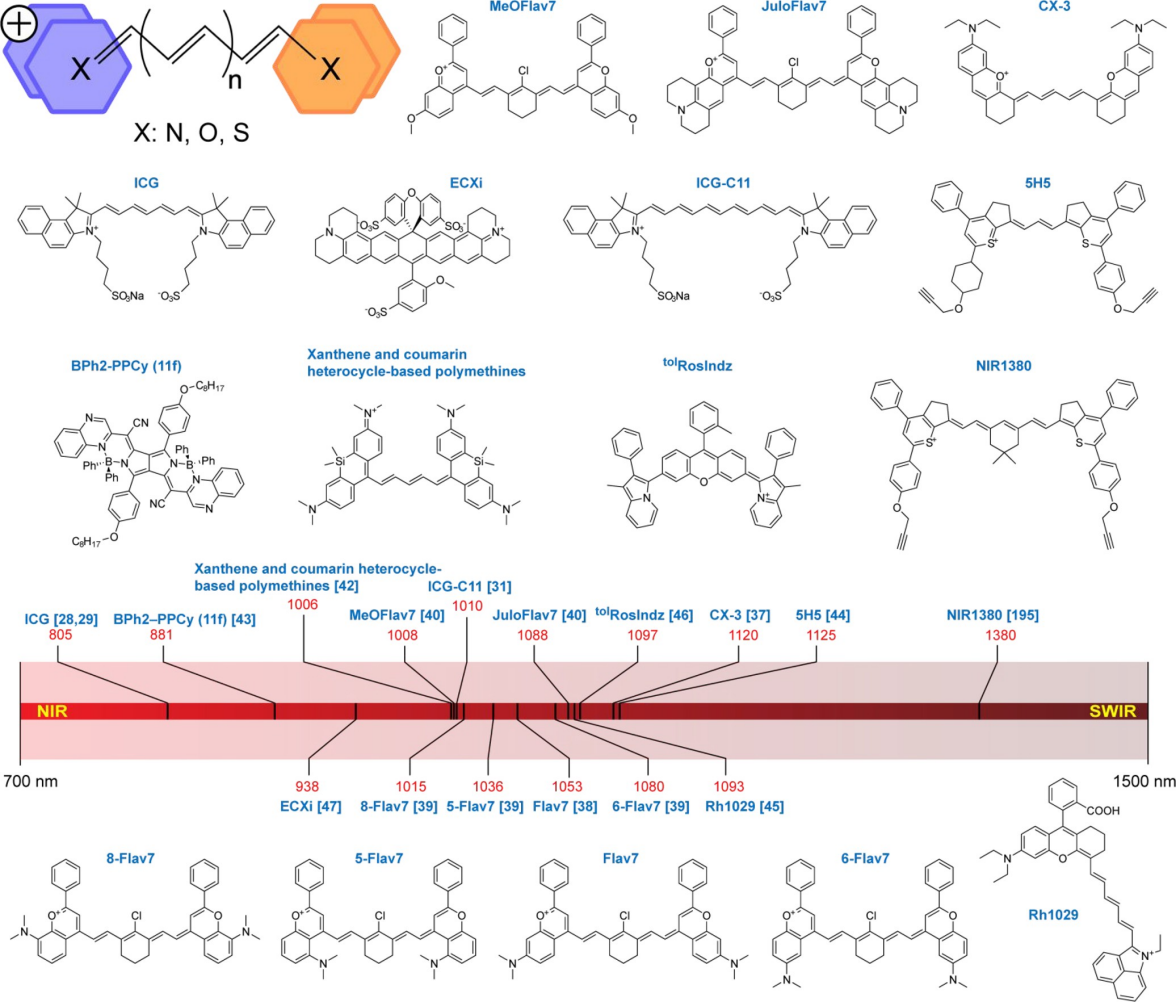
NIR/SWIR emitting small-organic-molecule fluorescent probes based on polymethine dyes. General structure of polymethine dyes together with examples of the molecules that belong to this category. The numbers in red show peak fluorescence wavelengths of each molecule observed in an aqueous environment. The numbers in the brackets indicate the reference numbers for each molecule.

The most common strategy to design NIR/SWIR fluorophores is based on a structural modification of polymethine dyes (Figure 1). Polymethines are chromophoric systems consisting of π-conjugated double-bond chains flanked at opposite terminal carbons with electron donor and electron acceptor groups. A reduction of the energy gap is achieved by extending the polymethine chain. An addition of every vinylene (−CH=CH−) unit leads to nearly a 100 nm bathochromic shift. The molar absorption coefficients and oscillator strength of polymethines increase noticeably as the chain length is extended. However, such a modification is generally accompanied by a significant decrease in photo- and thermal stability. The emission properties are ruled by the chain length and composition of the terminal groups. Within the extension of polymethine chains, the fluorescence efficiency of the generated series initially increases until it reaches a maximum, then decreases at longer chain lengths. In general, fluorescence quantum yields of longer conjugates are rather low, caused by a flexible chain structure that undergoes photoisomerization in the excited state.

#### 2.1.1 Indocyanine Green and Its Derivatives

The most known class of polymethine dyes is cyanines, the first dye synthesized in 1856 that revealed a cyan color. Recently, the spectroscopic characterization of NIR indocyanine green dye (ICG) revealed measurable long-tail fluorescence emission that stretches to 1500 nm in the SWIR region.^28,29^ Follow-up studies indicated that many other conventional NIR-polymethine fluorophores could also be employed for SWIR imaging, as they produce measurable fluorescence emission in this regime.^30^ Large efforts have been made to design and synthesize ICG analogues; ICG-C11 and ICG-C11–NHS fluorophores have absorption and emission maxima shifted to ca. 100 nm longer wavelengths.^31^ Water-soluble two-double bond extended ICG analogues emit beyond 1010 nm with QY up to 0.13%. The common issue of low chemical stability and reduced fluorescence intensity of polymethines in an aqueous environment can be overcome by proper charge balancing and the introduction of steric shielding.^29,32,33^ A flexible structure of polymethine dyes including indocyanine green and its derivatives causes a large conformational distribution, which results in a relatively broad spectral width. It is also important to note that spectroscopic properties including the spectral bandwidth depend largely on a local environment in general because the local environment has a significant effect on their conformational state.

#### 2.1.2 Polymethine Dyes with Heterocyclic Systems

Cyanine dyes with varied energy gaps have been designed through structure modifications that allowed the development of fluorophores with emission greater than 1000 nm.^34−36^ One of the very promising ways of structural modifications is to bridge large rigid heterocyclic systems with a short polymethine chain, which allows an expansion of the overall π-conjugation system while maintaining its structural stiffness. This structural modification led to the development of CX-dyes (abs/em: 1089–1140 nm) with much improved stability in an aqueous environment.^37^ The introduction of dimethylamino flavylium heterocycles as terminal groups allowed Cosco et al. to construct fluorophore 7-methine Flav7 (λ_em_ = 1045 nm), which exhibits fluorescence brighter than most of the previously existing SWIR polymethine dyes.^38^ Progressive spectral red-shifts have further been achieved by the introduction of the dimethylamino group at different positions of the flavylium heterocycle.^39^ An adjustment of spectral properties by a heterocycle modification on a 7-substituted flavone intermediate allowed the development of JuloFlav7 and MeOFlav7 SWIR dyes that are applicable for multispectral imaging purposes.^40^

A significant reduction in nonradiative channels was achieved in chromenylium dyes based on a flavylium polymethine scaffold.^41^ The introduction of silicon (SiMe_2_) into xanthene and coumarin heterocycle-based polymethines scaffolds imparts red shifts of similar magnitude as the extension of the polymethine chain itself, resulting in the development of a series of NIR/infrared fluorophores with fluorescence above 900 nm.^42^ Similarly, flavylium fragments can be conjugated to polymethine termini to form silylrhodapolymethines.^40^ Pyrrolopyrrole cyanines based on a rigidified heterocyclic ring core platform allow one to tune the absorption between 684 and 864 nm, while high fluorescence quantum yields (32–69%) are maintained.^43^ The introduction of tripyrylium heterocycles leads to the development 5H5 (absorption λ_max_ = 1069 nm, emission λ_max_ = 1125 nm).^44^

In another approach, the rigidification of the polymethine unit was achieved by the polyene bridge linkage in hybrid Rhodamine-benz[c,d]indolium dye analogues (Rh824, Rh926, and Rh1029) with absorption peaks ranging from 824 to 1029 nm and emission peaks ranging from 872 to 1093 nm.^45^ The SWIR emitting RosIndolizine dyes based on xanthene consist of mixed amine (rosamines) or oxygen/nitrogen (Rosol) donor groups to delocalize the positive charge over the entire π-conjugation system (^tol^RosIndz and ^Ph^RosIndz).^46^ The spectral properties of these systems fall short on the SWIR spectral region for absorption and emit primarily above 1000 nm. They have similar absorption profiles in an organic solvent with peaks at 930 nm that stretch well into the SWIR region reaching up to 1100 nm. ECX dyes based on a bisbenzo-C-rhodamine unit has been developed by modifying the unit with a bulky diphenyl ether moiety installed through a rigid spirolinkage, which hampers rotational freedom and prevents undesirable nonradiative quenching.^47^ ECX dyes have absorption and emission in the NIR region (abs 880 nm, em 915 nm) with a fluorescence quantum yield up to 1.4% in aqueous solution.

### 2.2 Donor-Acceptor Systems

**Figure 2:**
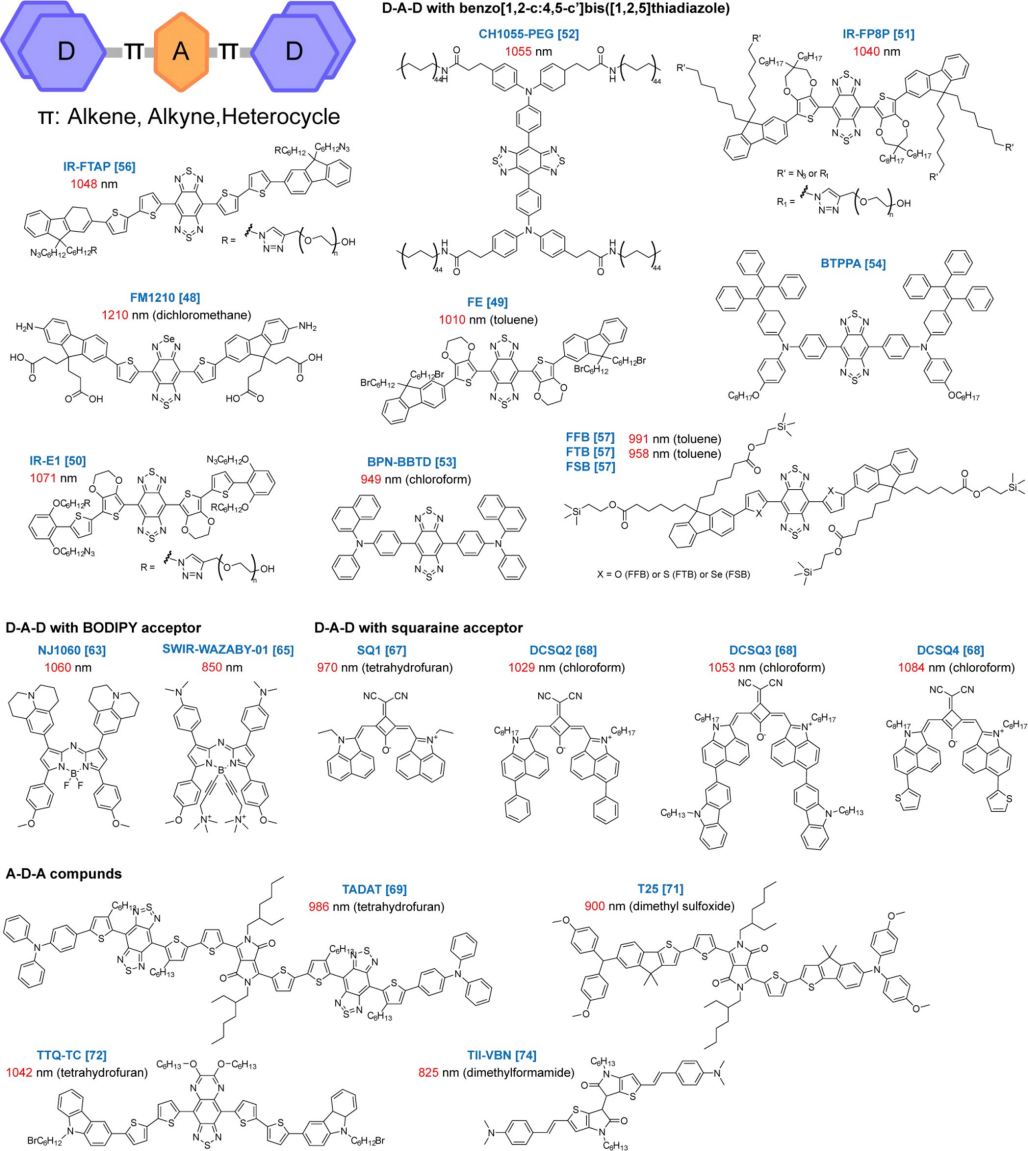
NIR/SWIR emitting small-organic-molecule fluorescent probes based on donor–acceptor systems. General structure of donor–acceptor dyes together with examples of the molecules that belong to this category. The numbers in red show peak fluorescence wavelengths of each molecule observed in an aqueous environment. The numbers in the brackets indicate the reference numbers for each molecule.

Another extensively exploited design that allows for fine-tuning of the energy gap in π-conjugated systems is based on a donor–acceptor (D–A) approach that utilizes control over the energetics of intramolecular charge transfer (ICT) from an electron-rich donor to electron-deficient acceptor moieties (Figure 2). The charge transferred from donor to acceptor can be systematically modulated by varying the strengths of the donor and/or acceptor units and improving the electronic interactions between them by incorporating the conjugated π-spacer, thus maintaining efficient control over the energy gap. The configuration of the D–A system can be further adjusted by considering their molecular symmetries and asymmetries (e.g., D-A, D-π-A, D-A-D, D-π-A-π-D, A-D-A, A-π-D-π-A). Thus, one can adjust the energy gap of the whole system by altering electron-donating and -withdrawing abilities of D and A, resulting in absorption and fluorescence at longer wavelength.

#### 2.2.1 D-A-D with Benzo[1,2-c:4,5-c′]bis([1,2,5]thiadiazole)

In general, molecules with expanded π-electron systems and introduced heteroatoms such as N, O, and S exhibit higher electronic ground-state energy. On the contrary, the incorporation of electron-withdrawing units such as -F usually lowers the energy of both electronic ground and excited states. Currently, the overwhelming majority of reported SWIR dyes are based on a D-A-D structure with benzo[1,2-c:4,5-c′]bis([1,2,5]thiadiazole) (BBT) as the electron acceptor unit. One of the merits of the BBT unit is the presence of the hypervalent sulfur atom that exhibits a high electron affinity. In addition, BBT tends to adopt a quinoid form stabilized by low resonance energy. Both contribute to an overall increase in its strength as an acceptor. By replacing the S atom with Se in the BBT skeleton, fluorescence emission can be shifted far beyond 1200 nm (e.g., FM1210).^48^ In the series of D-BBT-D SWIR fluorophores, spectral properties were successfully tuned by selecting donor units with proper electron-donating characteristics. For example, 3,4-ethylenedioxythiophene (EDOT, e.g., FE^49^ and IR-E1^50^), 3,4-propylenedioxy thiophene (PDOT, e.g., IR-FP8P),^51^ triphenylamine (TPA, e.g., CH1055-PEG),^52^ and *N*,*N*-diphenylnaphthalen-1-amine (BPN, e.g., BPN-BBTD)^53^ have been used as the donor. The electron-donating characteristic has also been tuned by further expansion of the donor structure (e.g., BTPPA),^54,55^ inserting a second donor (e.g., IR-FTAP),^56^ and altering donor heteroatoms (e.g., FFB, FTB, FSB).^57^ As stated above, the π-spacer also plays an important role in tuning the energy levels of the D-π-A-π-D type of chromophores, as it extends the π-conjugation that promotes/stabilizes the quinoid structure or introduces steric effects to control the overall twist affecting the ICT properties of the system.^56,58−61^

#### 2.2.2 D-A-D with BODIPY Acceptor

The 4,4-difluoro-4-bora-3a,4a-diaza-s-indacene (BODIPY) dyes, which, in principle, can be treated as a rigid type of cross-conjugated cyanine where the complexation with boron leads to stiffening of the conjugated polymethine chromophore, have recently gained great attention as an electron acceptor, since they extend the fluorescence of D-A-D systems into the SWIR region. By replacing the meso-methine group in the acceptor BODIPY core with an aza-N atom, an additional red shift in both absorption and fluorescence transitions was achieved.^62^ A new series of aza-BODIPY core-based dyes, which present prolonged emission in the SWIR region and high quantum yields, was developed by incorporating strong electron-donating groups in the 3,5-positions of aza-BODIPY (e.g., NJ1060).^63^ A bright NIR dye was also obtained using a dimeric-type aza-BODIPY analogue called pyrrolopyrrole aza-BODIPY (PPAB) with thiazole heteroaromatic ring as an enhanced electron-accepting unit and triphenylamine (TPA) units as donors.^64^ A substitution of fluorine atoms in the boron center with *N*,*N*-dimethylpropargylamine followed by a quaternization of amino groups allowed the development of a highly water-soluble aza-BODIPY whose fluorescence emission reaches 1200 nm in biocompatible solutions (e.g., SWIR-WAZABY-01).^65^ The BODIPY framework has also been utilized to develop H_2_S activable D-π-A probes with emission reaching more than 1200 nm.^66^

#### 2.2.3 D-A-D with Squaraine Acceptor

Squaraine dyes typically comprise an electron-accepting four-membered ring of squaric acid (diketocyclobutenediol) and two electron donors in the D-A-D pattern, forming a quasi-zwitterionic fluorophore. Squaraines exhibit exceptionally sharp and intense absorption associated with a strong fluorescent emission in the NIR region. Fluorescence in the SWIR window has rarely been reported due to the lack of a simple and universal design strategy. However, the optical properties of the NIR-squaraine dye SQ2 (benz [cd] indolium capped) constructed by ethyl-grafted 1,8-naphtholactam as donor units and squaric acid as an acceptor has been tuned to the SWIR spectral region by adding a malonitrile electron-withdrawing group (e.g., SQ1).^67^ In addition, a family of benz[cd]indole-capped SWIR squaraine dyes has been synthesized by coupling phenyl- (e.g., DCSQ2), carbazole- (e.g., DCSQ3), and thienyl- (e.g., DCSQ4) substituted benz [cd]indoles with squaric acid.^68^

#### 2.2.4 A-D-A Compounds

Recently, A-D-A compounds have become an attractive alternative for generating SWIR fluorescence because of their lower orbital energies and the tendency to increase exciton separation and charge transport compared to their D-A-D counterpart (e.g., TADAT).^69^ Optical properties of an A-D-A chromophore with CT character has been tuned simply by a complexation of Lewis acid to a basic site of the π-delocalized framework.^70^ Other examples of commonly used acceptor units include diketopyrrolopyrrole (DPP, e.g., T25),^71^ thiophene-thiadiazoloquinoxaline (TTQ, e.g., TTQ-TC),^72^ diphenylfumaronitrile (DBFN),^73^ derivatives of thienoisoindigo (TIIG, e.g., TII-VBN),^59,74^ and borondipyrromethenes (BODIPY).

### 2.3 Limitation of Small Organic Fluorophores for SWIR Imaging

A reduction of energy gap leads to a red shift of absorption and fluorescence spectra. Chromophores designed by the strategies described in Sections 2.1 and 2.2 exhibit a high fluorescence brightness in the NIR/SWIR spectral range. While polymethine dyes exhibit a large ε (ε > 10^5^ M^–1^ cm^–1^) with tunable peak fluorescence wavelength up to 1300 nm, most of the D-A-D dyes show a rather low ε (ε ≈ 10^3^ to 10^4^ M^–1^ cm^–1^) with peak fluorescence values up to 1050 nm. An important inherent limitation in the design of SWIR emitting fluorophores is that the decrease in energy gap (i.e., spectral shit to longer wavelength) leads to an increase of the rate constant of internal conversion (*k*_IC_) between the excited and ground states (i.e., nonradiative deactivation). According to the energy gap law, *k*_IC_ decrease exponentially with energy gap.^75^ Thus, a significant decrease of Φ_fl_ at longer wavelength is expected, in particular, in the SWIR spectral region.

In addition, a further drop in Φ_fl_ is often observed in an aqueous environment. In the case of polymethine dyes, water can reduce Φ_fl_ by up to threefold.^76^ A hydrogen-bond-assisted excited-state nonradiative deactivation pathway often predominates in dyes with an ICT character.^77−79^ All these limitations become a driving force toward engineering excited states of molecules in the context of intermolecular interactions to achieve brighter SWIR fluorescence (e.g., enhancing intramolecular charge transfer, reducing nonradiative processes through direct deuteration,^80,81^ increasing molecular stiffness, introducing shielding units to avoid undesirable molecular interactions,^51,56,58,82^ and shielding by self-protecting aggregates formation).

## 3 Small Organic Molecule-Based Fluorescent Nanoparticles

The organic SWIR fluorophores that we introduced in the previous section have a small size and mitigated toxicity, which are suitable for many imaging applications. However, an inevitable dark fluorescence of the SWIR small molecular probes because of their very small Φ_fl_ and relatively low ε, in particular, in the donor–acceptor type molecules, sometimes limits their imaging application. Therefore, SWIR probes based on molecular aggregates and nanoparticles that contain a large number of fluorophores are, in principle, a promising alternative to the small molecular probes. While the ε value of molecular aggregates and nanoparticles is greatly enhanced compared with that of small molecules, an inherent strong tendency of π–π stacking of planar organic molecules and strong electronic interaction between adjacent chromophores in aggregates state often cause notorious aggregation-caused quenching (ACQ) in which the molecules form less-emissive species such as excimers. Since the ACQ leads to a reduction in Φ_fl_, ACQ greatly impedes the practical use of organic fluorophores in aggregate states to imaging applications. To obtain molecular aggregates and nanoparticles with bright fluorescence, one must develop a strategy to avoid ACQ. This section introduces various approaches to avoid ACQ, including excited-state engineering (e.g., aggregation-induced conformation change and rigidification, formation of J-aggregates).

### 3.1 Excited-State Engineering through Aggregation

#### 3.1.1 Various Types of Molecular Aggregates

For more than a century after Sheppard first postulated aggregation of isocyanine dye in water^83^ followed by observations of unusual spectral behavior of pseudoisocyanine by Scheibe^84^ and Jelly,^85^ molecular aggregates have continuously attracted the attention of scientists across many disciplines because interaction between chromophores in aggregate states can significantly alter their spectroscopic properties beyond what can be achieved by controlling the molecular structure of individual chromophores. Organic dyes at a high concentration in a poor solvent tend to self-assemble to minimize interaction with the solvent. Weak intermolecular interactions govern spatial packing of the molecules in self-assembled (i.e., aggregate) states and lead to new photophysical properties, which deviate from those present in the molecules in a spatially isolated state.

**Figure 3:**
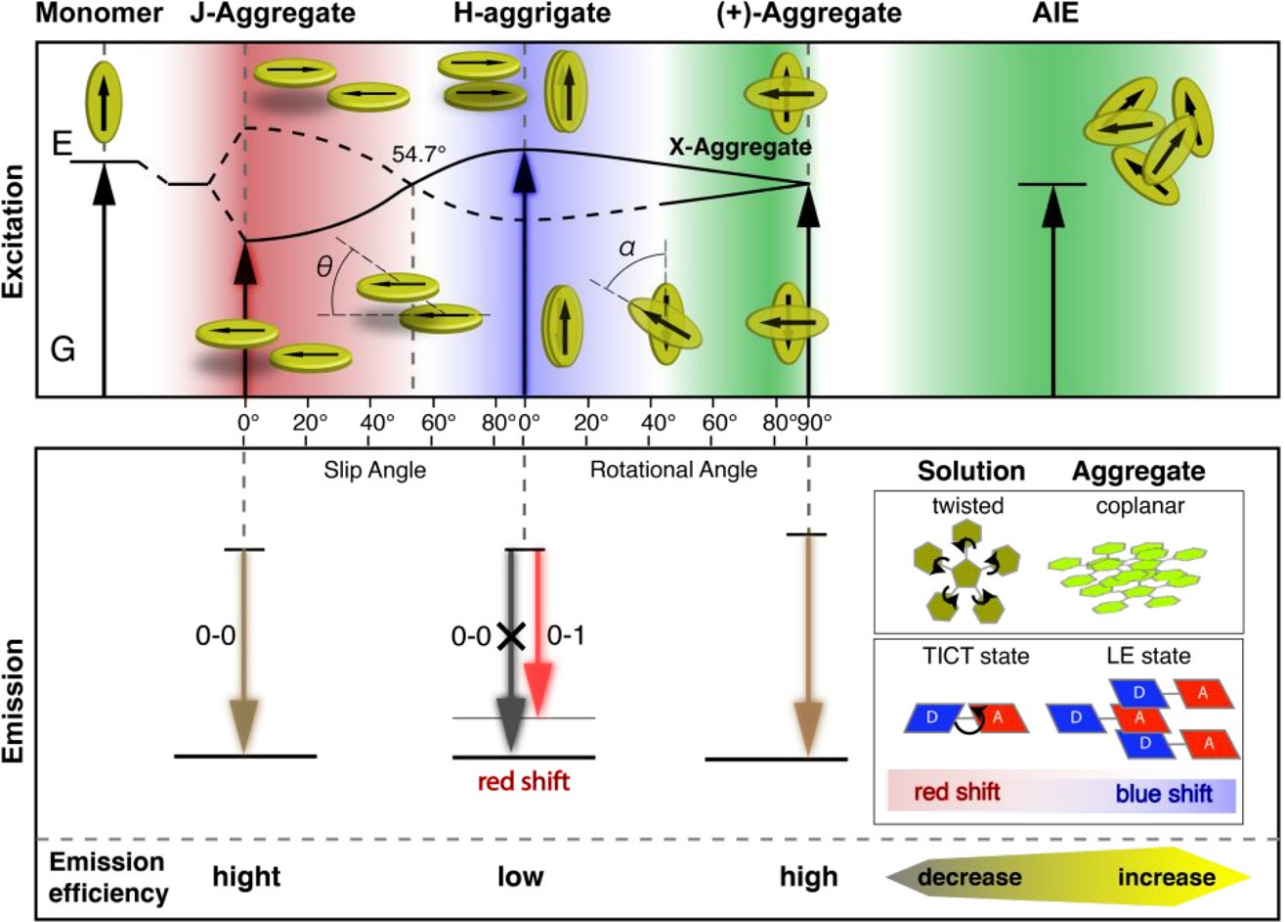
Exciton splitting diagram for aggregates with J-aggregate, H-aggregate, X-aggregate, and AIE configurations. Reproduced with permission from ref (88), copyright 2018 Wiley-VCH Verlag GmbH & Co.

According to the exciton model, when two rigid and planar π-conjugated molecules are confined in a crowded environment, their transition dipole moments start to interact strongly with each other.^86,87^ The excited-state energy of such a dimer system becomes resonantly split (Figure 3).^88^ Depending on the center-to-center distance and relative orientation of the transition dipole moments of adjacent molecules, basal dimer configurations are divided into different types of aggregates. In H-aggregates (cofacial stacking where chromophores stack parallel in a face-to-face order with slide pitch angle greater than 54.7°), only transition to the higher-energy exciton is allowed, leading to a blue-shifted absorption band observed in the UV/visible spectrum as compared to a constituent monomer form. The absence of optical coupling between the ground state and the lower-energy exciton implies that the H-aggregate usually has reduced fluorescence efficiency with respect to the isolated monomer.^89^ In rare cases, fluorescence of H-aggregates has been observed,^90^ in which the monomers form cross-packing layer structures^91^ or aggregates with a slight rotation of the coupled dye molecules.^92^

In J-aggregates (slipped stacking where chromophores stack in a head-to-tail arrangement with slide pitch angle less than 54.7°), the transition to a lower-energy exciton is allowed, leading to a distinct bathochromically shifted absorption band as compared to the free monomer. Because the lower-energy exciton state is optically allowed, J-aggregation usually leads to preservation or even increase in the fluorescence efficiency compared to the isolated chromophore. In X-aggregates (crossed stacking where molecular planes remain parallel with each other, but the long axis of molecules forms a rotational angle around the stacking axis), the rotation of the adjacent transition dipoles reduces the energy-level splitting of the excited state, leading to an optically allowed transition from both of the split excitonic states. The shrinkage of dipole interaction results in spectral signatures of X-aggregates similar to the monomeric molecule, including preservation of the fluorescence emission. While cross-dipole stacking has been addressed as a preferred motif for aggregated state emission, molecules possessing predominant X-type stacking are still quite rare. This is mainly due to the difficulty of the molecular design and structural modification requirements.^93−96^

#### 3.1.2 J-Aggregates

One of the most specific features of J-aggregates is the appearance of a new excitonic band (J-band) in the absorption spectrum, which is red-shifted with respect to the monomer spectrum (see the Supporting Information: definition of J-aggregates).^89^ The line width of the absorption band becomes severely narrowed as a result of the occurrence of coherently coupled excited states and delocalized molecular excitons. J-Aggregates act as one unit that consists of coupled chromophores with a strongly increased transition dipole moment and, thus, exhibit strong light absorption in a narrow range of energies. The amplitude of spectral narrowing is proportional to the number of coherently coupled molecules. The fluorescence matches the absorption spectra and therefore shows a narrow spectral width. The limited space for vibrational and rotational relaxation of the associated monomers in the aggregates restricts nonradiative relaxations. This, in combination with an increase in the transition dipole moment of the whole system, leads to an enhancement in the fluorescence brightness. Another characteristic feature of J-aggregates is a significant reduction in the radiative lifetime known as superradiance.^97^

The self-assembly of dyes can be controlled at the level of molecular design through substituent coupling so that the bulkiness of the donor substituent leads to strong H-type or strong J-type exciton coupling.^98−100^ Alternatively, the formation of specific self-assembled aggregates can be guided by several parameters,^101^ including salt concentration, temperature,^102,103^ pH, and solvent.^104,105^ For instance, the formation of either J-aggregates or H-aggregates of 1,1′-diethyl-2,2′-cyanine (chloride, iodide, etc.) known as pseudoisocyanine (PIC) depends on the concentration of the dye or its counterions (salt concentration) and temperature of precipitation process.^89^ A reversible switching between different aggregation states has been reported by combining the two approaches.^106^

#### 3.1.3 J-Aggregates for Bioimaging Application

The pronounced bathochromic shift in the absorption spectrum makes J-aggregates the most promising materials from the perspective of bioimaging applications. In some cases, it can lead to over 100 nm red shift of both absorption end fluorescence compared to monomer dye.^107^ Because of the spectral narrowing, they can act as a color purity emitter^108^ that enables multiplexed imaging. In addition, an enhanced molar extinction coefficient has resulted in brighter fluorescence. J-Aggregates (in majority polymethine) usually form highly ordered tubular structures or sheet-like morphologies, and it is often difficult to obtain stable particles, as they exist only at high dye concentrations or under special stabilizing conditions, which hamper their application in fluorescence microscopy. Some non-polymethine J-aggregates stably exist in an aqueous environment.^109,110^ However, most of the small molecules that form J-aggregates in an aqueous environment have a low fluorescence quantum yield. Stable J-aggregates in aqueous environments have also been obtained by encapsulating (i.e., molecules are loaded into an empty interior of a shell or capsule-type component without a separation between loaded molecules) them in nanostructures, including polymer micelles, liposomes, mesoporous silica nanoparticles, and carbon nanotubes.

### 3.2 Fluorescent J-Aggregate Nanoparticles

#### 3.2.1 Cyanine Dyes

**Figure 4:**
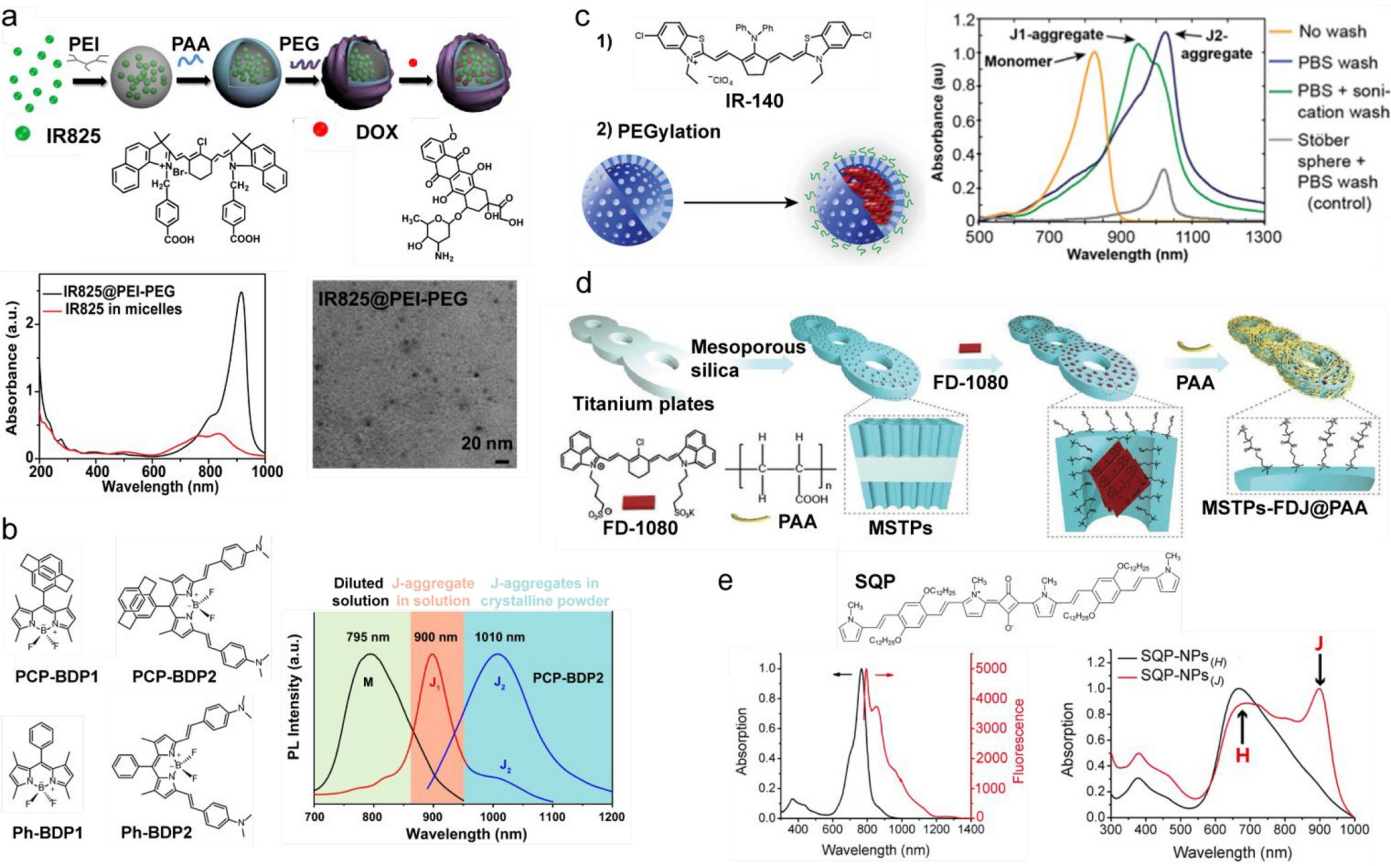
Fluorescent J-aggregate nanoparticles by encapsulating in nanostructures. (a) Schematic illustration to show the fabrication process of IR825@PEI–PEG/DOX (top). Absorption spectra of IR825@PEI–PEG (black) and IR825 in PEGylated micelles (red) (left). Transmission electron microscopy image of IR825@PEI–PEG. (b) Chemical structures of PCP-BDP1, PCP-BDP2, Ph-BDP1, and Ph-BDP2 (left). Fluorescence spectra of PCP-BDP2 in diluted dichloromethane (DCM) solution (10 μM) (black line, “M” refers to monomer), THF–water binary solvents (10 μM, 1:9, v/v) (red line, J1-band), and the crystalline powder (blue line, J2-band) (right). (c) Schematic illustration describing the stabilization of IR-140 J-aggregates in hollow mesoporous silica nanoparticles (HMSNs) to result in biocompatible SWIR-emissive contrast agents (left). Absorption spectra of the IR-140 monomer and J-aggregates in HMSNs (right). A washing step facilitates the J-aggregates formation. (d) Schematic illustration of the preparation of titanium plates coated with a silica layer encapsulated by SWIR emitting J-aggregates (MSTPs-FDJ@PAA). (e) Chemical structure of SQP (top). Absorption (black) and fluorescence (red) spectra of SQP in THF (left). Absorption spectra of SQP-NPs-NPs in THF (SQP-NPs_(H)_) and in DCM (SQP-NPs_(J)_) (right). (a) Reproduced with permission from ref (111), copyright 2015 Elsevier Ltd. (b) Reproduced with permission from ref (118), under CC BY 4.0. (c) Reproduced with permission from ref (114), copyright 2019 American Chemical Society. (d) Reproduced with permission from ref (115), copyright 2021 Wiley-VCH GmbH. (e) Reproduced with permission from ref (116), copyright 2018 The Royal Society of Chemistry.

IR825 cyanine dye encapsulated in low-molecular-weight cationic polymer polyethylenimine (PEI) form IR825@PEI J-aggregates with greatly enhanced and red-shifted NIR absorbance at 915 nm (Figure 4a).^111^ Dicarboxyphenyl cyanine (DCP-Cy) tended to form J-aggregates with a pronounced spectral red shift to 934 nm (from 789 nm in the monomeric form).^112^ Liposomes-entrapped DCP-Cy dye aggregates had a narrow absorption spectrum (full width at half-maximum (fwhm) = 25 nm). FD-1080 cyanine dye coassembled with dipalmitoylphosphatidylcholine (DMPC) lipid via a film dispersion method allowed one to prepare nanoparticles with an average size of 110 nm. Steady-state absorption and fluorescence spectra of FD-1080/DMPC nanoparticles revealed a small (10 nm) Stokes shift and a large red shift (300 nm) as compared to the FD-1080 monomer with spectral peak located at 1360 and 1370 nm, respectively.^113^ The fluorescence lifetime was shortened from 312 to 172 ps.

Inorganic nanocarriers have also been utilized to stabilize J-aggregates. For instance, NIR cyanine fluorophore IR-140 formed J-aggregates by loading the molecules into 85 nm size hollow mesoporous silica nanoparticles (Figure 4c).^114^ Silica particles contain negatively charged pores that help to associate with the cationic IR-140 molecules. The formed J-aggregates displayed a red shift of the absorption spectra (from 826 nm in the monomer form to 1040 nm in the J-aggregates). A mesoporous silica layer with vertical channels grown on the surface of titanium-plate implants provided a confining space for the formation of the FD-1080 J-aggregates, which has been applied to an imaging-guided osteosynthesis (Figure 4d).^115^

#### 3.2.2 Squaraine Dyes

Squaraine-based nanoparticles with bright fluorescence near 1100 nm have been used for SWIR imaging-guided phototermal therapy on MCF-7 (Michigan Cancer Foundation-7) tumor-bearing mice (Figure 4e).^116^ Particles exhibiting J-type interaction of squaraine dye coprecipitated with an amphiphilic copolymer (PEG-*b*-PPG-*b*-PEG, Pluronic-127; PEG = poly(ethylene glycol)) formed stable spherical-shaped particles with a diameter of 82 nm, large ε (ε_max_ ≈ 0.5 × 10^5^ M^–1^ cm^–1^), small Stokes shift (10 nm), and Φ_fl_ of 0.0545%. Bis(squaraine) dye Bis-SQ composed of two collinearly connected chromophores displayed a strongly bathochromically shifted absorption maximum at 961 nm and a fluorescence peak at 971 nm due to an intramolecular J-type coupling in chloroform.^117^

#### 3.2.3 BODIPY Dyes

An SWIR fluorescence emitted from J-aggregates of BODIPY dye has been demonstrated by introducing a paracyclophenyl (PCP) group to the meso-position of 3,5-bis-*N*,*N*-dimethylaminostyryl BODIPY (Figure 4b).^118^ In this system, an interaction between PCP and the *N*,*N*-dimethylaminophenyl group predominates the molecular packing and plays a key role in the slip-stacking of the BODIPY core. Further, by encapsulation of PCP-BDP2 J-aggregates in Pluronic F-127, spherical nanoparticles (average diameter of 70 nm) with absorption and fluorescence peaks at 750 and 1010 nm, respectively, were obtained.

#### 3.2.4 Fluorescent J-Aggregate Nanoparticles without Encapsulation

Indocyanine green in an aqueous solution mainly aggregates with H-type packaging, showing two absorption peaks at 780 and 715 nm. However, under extended water bath heating at 65 °C, these two peaks gradually decrease, and a new absorption peak at 895 nm appears (115 nm red shift in the absorption peak compared to free ICG), indicating transformation into J-aggregates.^119^ The aggregates reveal an average diameter of ∼91.7 nm and remain stable in various solutions. Once the aggregates are internalized into cells, they disassociate into free ICG.^120^

**Figure 5:**
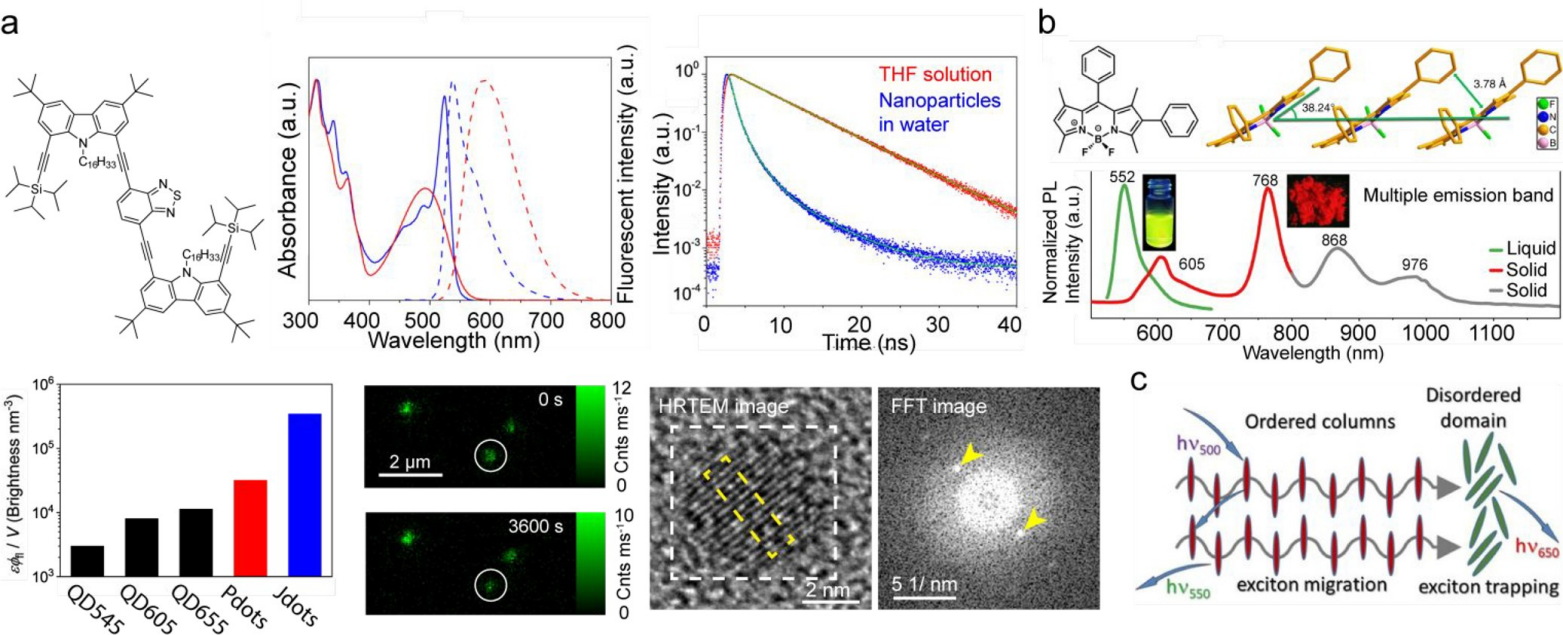
Fluorescent J-aggregate nanoparticles without encapsulation. (a) Chemical structure of the CzBTCz molecule (top left). Absorption (solid lines) and fluorescence (dashed lines) spectra of CzBTCz in THF (red lines) and CzBTCz nanoparticles dispersed in water (blue lines) (top middle). Fluorescence decays obtained for CzBTCz in THF (red) and the CzBTCz nanoparticles dispersed in water (blue) (top right). Fluorescence brightness per unit volume calculated for Qdots 545 (QD545), Qdots 605 (QD605), Qdots 655 (QD655), (CzBT)_*n*_ polymer dots (Pdots), and CzBTCz Jdots (Jdots) under the one-photon excitation condition (bottom left). Fluorescence images of the CzBTCz Jdots captured at time point 0 and 3600 s of continuous illumination^109^ (bottom middle). High-resolution TEM image of a single CzBTCz nanoparticle with corresponding fast Fourier transform (FFT) image of the region marked with a white box (bottom right). (b) Structural details (top) and fluorescent properties of aryl-substituted BODIPY dye, BDP1 (bottom). Molecular packing diagrams of BDP1 at room temperature extracted from single-crystal XRD data. Normalized photoluminescence spectra of BDP1 in solution (green curve) and microcrystalline powder state (red and gray curves).^126^ (c) Pictorial representation of the putative exciton migration and trapping model.^125^ (a) Reproduced with permission from ref (109), copyright 2021 American Chemical Society. (b) Reproduced with permission from ref (126), under CC BY 4.0. (c) Reproduced with permission from ref (125), copyright 2019 Wiley-VCH Verlag GmbH & Co.

It is important to stress that the currently reported J-aggregates formed in an aqueous environment, in particular, polymethine-based J-aggregates, generally showed relatively low Φ_fl_.^121−124^ The self-assembly of dye molecules into large J-aggregates may introduce morphological disorder and defects that quench excitons, leading to greatly reduced Φ_fl_. The amorphous aggregate domains that act as excitation traps for J-aggregates were observed in BODIPY-based nanomaterials. The presence of such amorphous domains at an interface with the ordered crystalline J-aggregate columns promotes energy transfer from the crystalline phase to the amorphous phase, with subsequent fluorescence from the weakly emissive more red-shifted aggregates (Figure 5c).^125^ Successive domino-like energy transfer in 2-/2,6-aryl substituted BODIPY dyes (BDP1) has been observed from high to step-wisely lower-located energy levels corresponding to different excitation states of aggregates, which leads to multifluorescence emissions across red and NIR-SWIR in their aggregation states (Figure 5b).^126^ A spectral shift of the fluorescence emission was observed from 552 nm in tetrahydrofuran (THF) to 605, 768, 868, and 976 nm in the multifluorescence microcrystalline states (formed at varied volumetric factions of water and THF) with a decent Φ_fl_ (Φ_fl_ = 10%).

Unlike J-aggregates assembled from benzothiadiazole-based charge-transfer complexes, a V-shaped D-A-D system composed of benzothiadiazole and two electron-donating thiophene (or bithiophene) units formed a dark state associated with an intermediate aggregate form, which was responsible for the observed fluorescence quenching.^127^ On the contrary, studies of the carbazole (Cz)-benzothiadiazide (BT)-based D-π-A-π-D molecule (CzBTCz), which mimics the generic cyanine dye structure consisting of two large heterocyclic components connected by a π-conjugated linker, revealed the formation of a highly emissive J-aggregate (Figure 5a).^109^ The 3.5 nm size CzBTCz nanoparticles showed a narrow absorption spectrum (fwhm = 27 nm), enhanced peak molar extinction coefficient, and near-unity Φ_fl_ (Φ_fl_ = 0.95). In contrast to CzBTCz, CzBT composed of the same structural motif but different symmetry (D-π-A) did not show any sign of J-aggregates upon the fabrication of the nanoparticles. The absence of the J-aggregate formation in the CzBT nanoparticles illustrates a critical role played by the shape of the molecule on their spatial packing inside the particles.

### 3.3 Small Molecules Embedded in Polymer Nanoparticles

Fluorescence properties of molecules can be modified by a rigidification in aggregated states (i.e., the conformation of the molecules is self-rigidified by a direct contact between each monomer in a densely packed environment) or by embedding/doping (i.e., introduction of a small quantity of loading molecules into host matrices in which a direct interaction between loaded molecules is minimized) in rigid matrices (i.e., conformation of the molecules is rigidified by host molecules), instead of designing a molecular skeleton with a high structural rigidity.^128^ Embedding fluorophores in a matrix (e.g., polymer, lipid, etc.) protects them from quenching caused by water. In addition, this introduces a hardness/stiffness of the microenvironment, which reduces the structural flexibility of dye molecules (i.e., minimizes nonradiative channels). Thus, the embedding of the molecules often results in enhanced Φ_fl_. Most of the small fluorophore-embedded polymer nanoparticles are based on spectrally neutral hosts (e.g., poly(ethylene glycol) (PEG), poly(methyl)methacrylate (PMMA), and Pluronic). However, the use of conjugated polymers (CPs) as a host offers a great advantage. Such polymers exhibit a strong light-harvesting ability because of the efficient excition migration along the polymer backbones, facilitating efficient energy transfer to low-energy acceptors (i.e., molecular wire effect). The careful pairing of CP energy donors with suitable energy-acceptor dyes allows one to obtain brighter fluorescence probes due to the signal amplification provided by the energy donor.^129,130^

### 3.4 Conformational Rigidification and Fluorescence Enhancement

**Figure 6:**
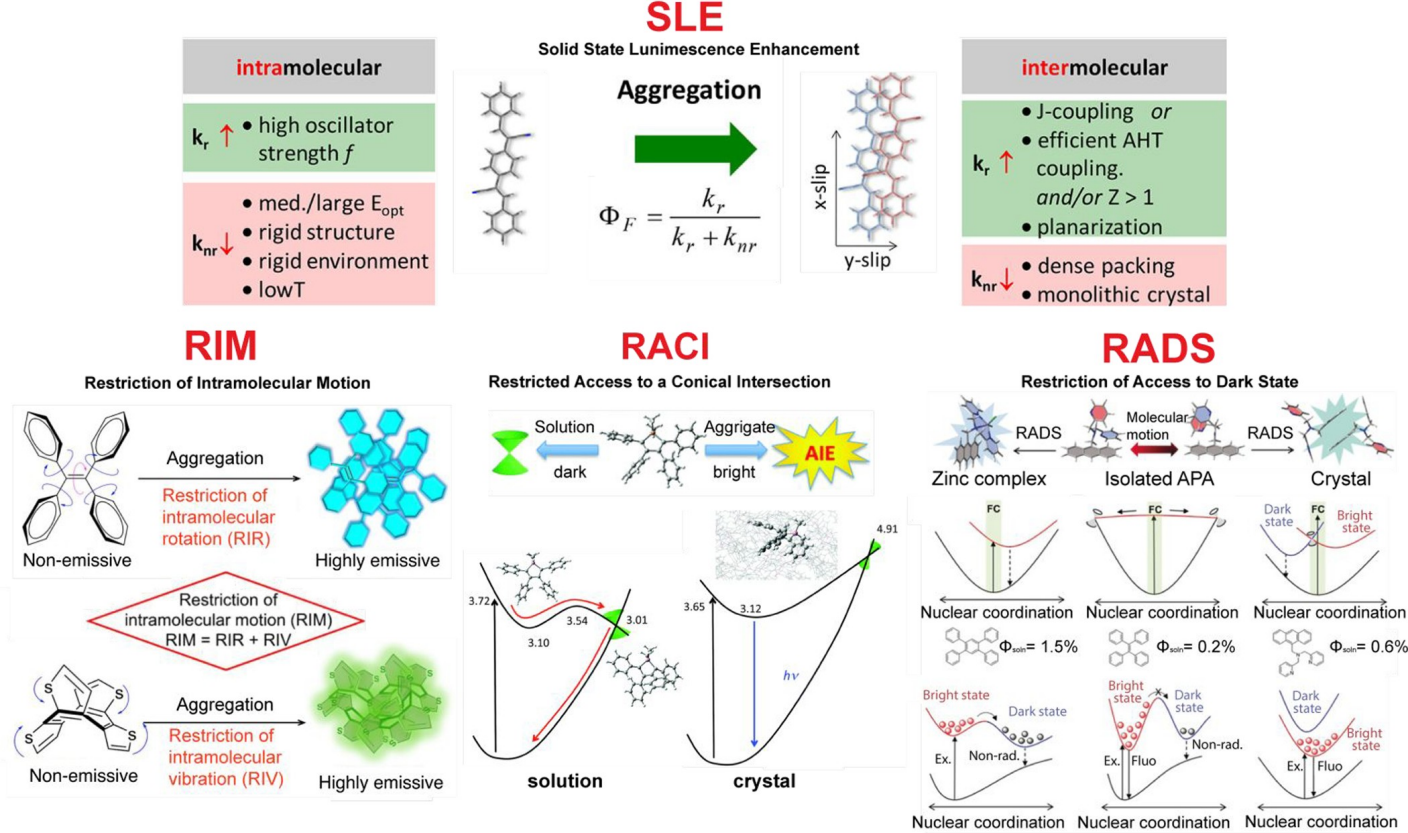
Conformational rigidification and fluorescence enhancement. Current models applied to explain observed fluorescence enhanced in aggregated states of π-conjugated materials; SLE: solid state luminescence enhancement (intramolecular vs intermolecular contributions and radiative vs nonradiative contributions),^137^ RIM: restriction of intramolecular motion,^133^ RACI: restricted access to a conical intersection,^136^ and RADS: restriction of access to a dark state.^142^ Reproduced with permission from refs (133, 136, 137), and (142), copyright 2016 The Royal Society of Chemistry, 2017 American Chemical Society, 2020 Elsevier Ltd., and 2019 Wiley-VCH Verlag GmbH & Co.

Fluorescence brightness that can be achieved by doping/embedding common planar dye molecules into matrices (e.g., polymer nanoparticles) is limited because increased dye doping is plagued by ACQ. Recently, special emphasis has been given to molecules that possess dim fluorescence in solution but become bright in the aggregate or solid state (solid-state luminescence enhancement, SLE) (Figure 6). This long-known phenomenon has received a new boost in its now-popularized term aggregation-induced emission (AIE, see the Supporting Information: Definition of aggregation-induced emission). A history of the development of the theory explaining this phenomenon from a broad perspective can be found in a recently published essay.^131^ In brief, excitation energy in “floppy” molecules with built-in flexibility or free rotating groups (e.g., rotatable aromatic rings) can be nonradiatively dissipated when their motions are not perturbed (as in the case of solution), resulting in these molecules being nonemissive. However, when an aggregation occurs or when a molecule is introduced into a rigid microenvironment, the motional freedom becomes restricted, and light emission is turned on or substantially enhanced. The main mechanisms associated with motions are restriction of intramolecular rotations (RIR), restriction of intramolecular vibrations (RIV), and restriction of intramolecular motions (RIM) (Figure 6).^132,133^ The current paradigm in photochemistry (unreasonably overlooked in the materials science community) points out that nonradiative deactivation from the excited-state molecules involves a conical intersection (CI).^134−139^ Prominent examples for the appearance of a CI are tetraphenylethylene^140^ and cyanines.^141^ The magnitude of vibronic interactions approaches infinity at the CI, where the S_1_ and S_0_ degenerate, from which the excitons decay nonradiatively. Molecular motions that lead to the CI geometry can be restricted upon aggregation, and this restriction of access to the conical intersection (RACI) restores fluorescence emission (Figure 6).^136^ This model explains the AIE mechanism and is consistent with the RIM mechanism, since the intramolecular motions responsible for AIE are those leading to the intersection. For heteroatom-containing systems, restriction of access to dark state (RADS) has been proposed to complete the picture of the AIE mechanism (Figure 6).^142^

#### 3.4.1 Anti-Antiaggregation-Caused Quenching and Aggregation-Induced Emission

**Figure 7:**
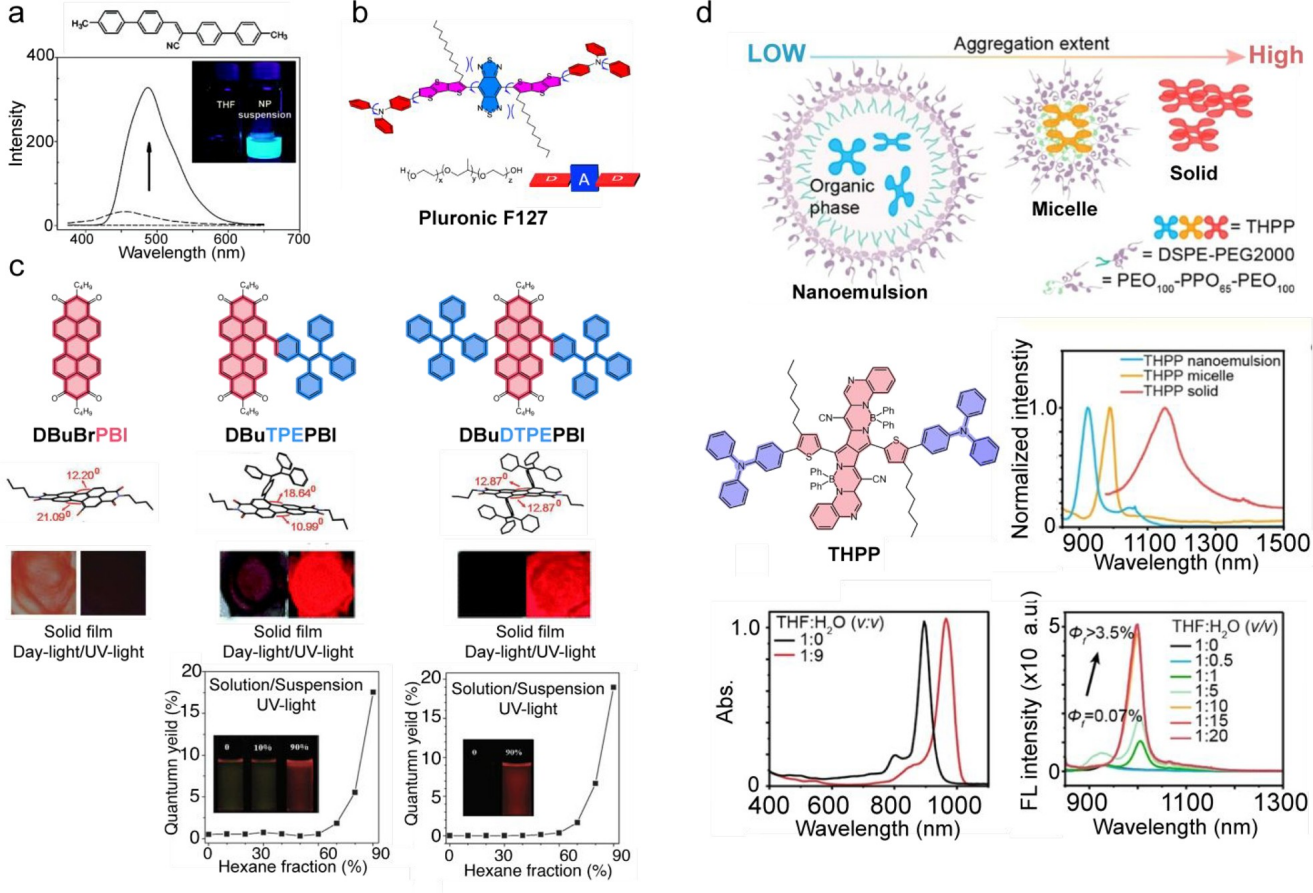
Fluorescence enhancement in small-organic molecule-based fluorescent nanoparticles through conformational rigidification. (a) Chemical structure of cyano-substituted stilbenic dye, 1-cyano-trans-1,2-bis(4′-methylbiphenyl)-ethylene (CN-MBE) (top). Fluorescence spectra of CN-MBE in THF solution and NP suspensions (80 vol % water in THF) (bottom).^96^ (b) Schematic illustration of the chemical structure of a D-A-D-type molecule, TT3-*o*CB, and its intramolecular motions in a solution phase, which are suppressed in the aggregation state.^61^ (c) Molecular structures of the non-, mono-, and di-TPE substituted perylene bisimides with optimal structures of DBuBrPBI, *N*,*N*′-dibutyl-1-(4-(1,2,2-triphenyl)vinyl)phenylperylene-3,4:9,10-tetracarboxylic acid bisimide (DBuTPEPBI), and DBuDTPEPBI calculated by the semiempirical AM1 method (top). The photographs display the solution cast films of perylene bisimides shown above taken under daylight (bottom, left columns) and 365 nm UV light (bottom, right columns). Variation in Φ_fl_ of DBuTPEPBI and DBuDTPEPBI in hexane/DCM mixtures with varied hexane fraction (right). The insets show fluorescence images (taken under 365 nm UV light) of the corresponding mixtures.^144^ (d) Schematic illustration describing different aggregation extent of THPP in the nanoemulsion, micelle, and solid (top). Chemical structure of THPP (middle left). Normalized fluorescence spectra of THPP nanoemulsion, THPP micelle, and THPP solid (middle right). Absorption spectra of THPP in THF and THF/water = 1:9 (v/v) solution (bottom left). Fluorescence spectra of THPP in different ratios of THF/water solutions (bottom right).^152^ (a) Reproduced with permission from ref (96), copyright 2011 American Chemical Society. (b) Reproduced with permission from ref (61), copyright 2020 American Chemical Society. (c) Reproduced with permission from ref (144), copyright 2012 The Royal Society of Chemistry. (d) Reproduced with permission from ref (152), copyright 2020 Wiley-VCH Verlag GmbH & Co.

AIE fluorophores allowed one to generate bright NIR emissive nanoparticles by avoiding the common ACQ effect.^143^ The common AIE-inspired design of D-A-D molecules that gain fair-emissive NIR/SWIR nanoparticles is based on an approach known colloquially as “lesser of two evils” and can be illustrated by an example of perylene bisimide derivatives (PBIs) (Figure 7c). PBIs have a quasi-unity fluorescence quantum yield in dilute solutions (*N*,*N*′-dibutyl-1-bromoperylene-3,4:9,10-tetracarboxylic acid bisimide (DBuBrPBI), Φ_fl,sol_ = 95.2%), but they become faintly or even nonemissive in aggregated or solid states. A molecular rotor tetraphenylethenyl (TPE) is introduced to achieve ACQ-to-AIE transformation. The new PBI substituted with TPE shows very dim fluorescence in a dilute solution (*N*,*N*′-dibutyl-1,7-di(4-(1,2,2-triphenyl)vinyl)phenyl-perylene-3,4:9,10-tetracarboxylic acid bisimide (DBuDTPEPBI), Φ_fl,sol_ = 0.07%), whereas upon aggregation the fluorescence intensity is recovered to a large extent (DBuDTPEPBI, Φ_fl,aggr_ = 18.9%).^144^ Thus, by the cost of fluorescence emission in organic solvents, one can gain partial fluorescence recovery in the aggregated state. TPE is a bulky substituent with a propeller-like shape; thus, the attachment of TPEs to the PBI core strongly distorts the π–π stacking between the PBI units that reduce ACQ. On the basis of a consecutive incorporation of common rotors (triphenylamine (TPA), TPE) as electron-donating groups that are directly conjugated to the acceptor unit or introduced to the donor groups, a number of new D-A-D dyes have been developed.^52,61,145−148^ Furthermore, by a coprecipitation with host polymers, bright and stable AIE nanoparticles have successfully been developed.

#### 3.4.2 Aggregation-Induced Conformational Change and Fluorescence Enhancement

Another class of π-conjugated organic molecules that shows high photoluminescence generally possesses a unique built-in molecular “elastic twist” feature that allows for large torsional or conformational changes upon aggregation (Figure 7a).^96^ In general, such systems possess a cyano-substituted stilbenic π-conjugated backbone, which plays an important role in achieving the synergistic combination of aggregation-induced planarization and specific intermolecular interactions. This results in dramatically enhanced emission upon aggregation (aggregation-induced enhanced emission, AIEE).

#### 3.4.3 Effect of Twisted Intramolecular Charge Transfer on Fluorescence Brightness

Twisted intramolecular charge transfer (TICT) is an electron-transfer process that occurs upon photoexcitation in molecules that usually consist of donor (D) and acceptor (A) moieties linked by a single bond.^149^ Upon photoexcitation in a nonpolar solvent, the locally excited ICT state of the D-A molecule is in equilibrium with solvent molecules, and its quasi-planar conformation stabilized by electronic conjugation leads to a sharp emission spectrum. However, in a polar environment, upon photoexcitation, a fast intramolecular electron transfer from the D to the A part of the molecule accompanied by intramolecular D-A twisting around the single bond occurs, which leads to a total charge separation between the D and A units and a nearly perpendicular alignment of the D and A units. In the new equilibrium, such a charge-separated twisted molecular conformation is stabilized by the polar solvent. Therefore, the TICT state has lower energy compared with an excited ICT state initially formed, which leads to the red shift of the emission spectrum. In addition, because of the vulnerability of the TICT state to various quenching processes and nonradiative relaxation pathways, the emission intensity can be weakened.

The excited TICT state might be suppressed by an inhibition of conformational rotation either by embedding the molecule into a rigid matrix^150^ or by the formation of a densely packed aggregated state of the molecule.^109^ The less twisted conformation of the molecule leads to blue-shift emission from the locally excited ICT state with an enhancement in the emission intensity. When there is a space for rotational motion of the molecule in a solid state, in some cases, a bright red-shifted fluorescence from a TICT state of the molecule was observed in a solid state.^151^

#### 3.4.4 Synergetic Effect of Conformational Rigidification and Other Factors

A recent study reported that a synergetic effect of conformational rigidification that leads to AIE and suppression of access to the TICT state greatly enhances fluorescence brightness of small-molecule fluorophores in an aggregate state. A D-A-D-type molecule, TT3-*o*CB, consisting of a benzobisthiadiazole (BBTD) acceptor and triphenylamine (TPA) donor that also acts as molecular rotor displayed dim fluorescence in a solution, whereas they exhibited fivefold brighter fluorescence in the aggregate state (Figure 7b).^61^ The observation has been attributed to the aggregation-induced restriction of the intramolecular motion of TPA, aggregation-induced inhibition of access to TICT state, and suppression of intermolecular cofacial π-π interactions by the intramolecular twist introduced by aliphatic side chains.

A fluorescent probe, THPP, based on the rigidified planar core structure of di-BODIPY, showed roughly an order of magnitude higher molar extinction coefficient than conventional D-A-D systems (Figure 7d).^152^ Benefiting from an extended π-conjugation system of di-BODIPY, the absorption and emission wavelengths in polar solvents shifted to 900 and 920 nm, respectively. An incorporation of the free rotating TPA groups led to a low Φ_fl_ in polar solvents due to the TICT effect. The restriction of the formation of its TICT state led to a 150-fold enhancement in Φ_fl_ in a nonpolar solvent. The aggregation of the THPP molecules in water/THF mixtures revealed a further increase in Φ_fl_ that was accompanied by a shortened fluorescence lifetime, a strong red-shift of the fluorescence spectra (1006 nm), and a spectral narrowing (fwhm from 65 to 36 nm). These characteristics were attributed to the synergetic effect of the suppression of the TICT state and the formation of J-aggregates upon the aggregate formation.

### 3.5 Counterion-Guided Small Organic Nanoparticles

**Figure 8:**
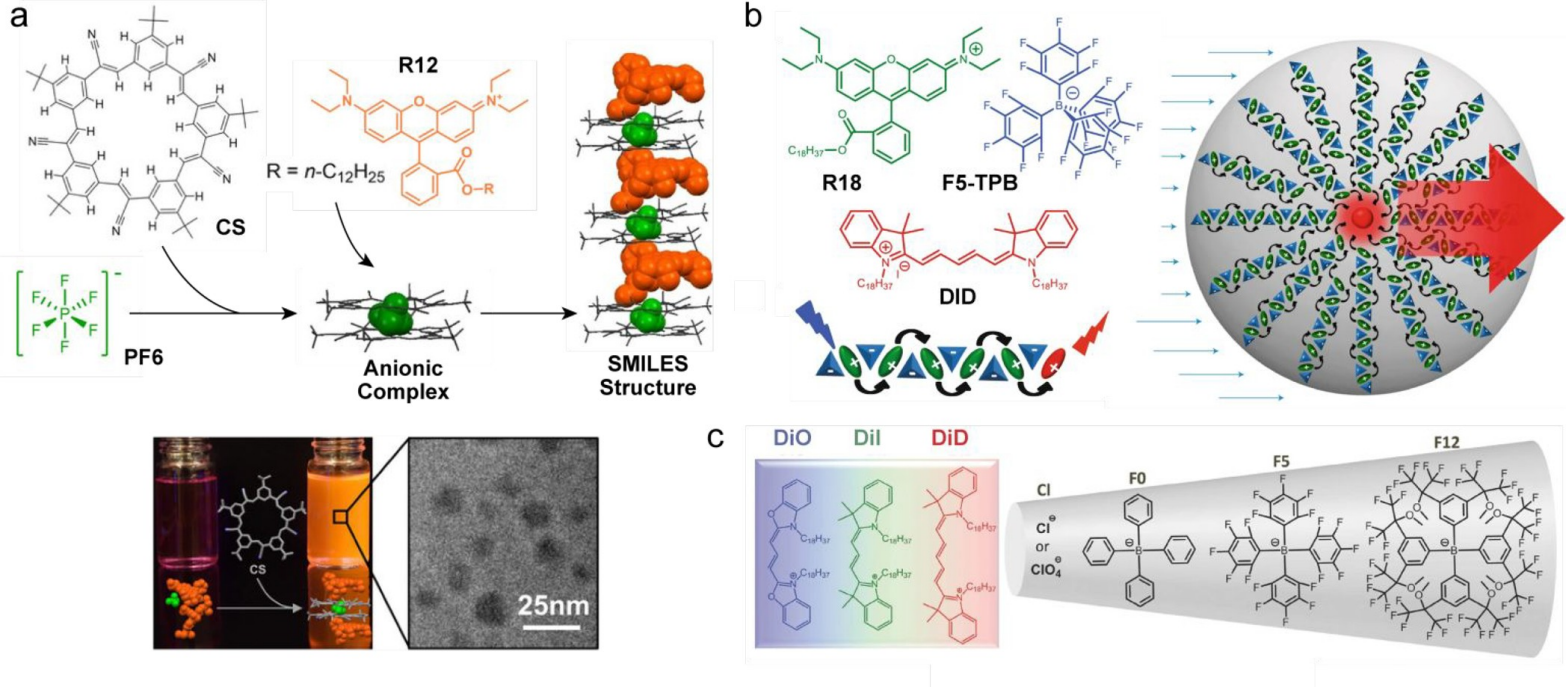
Counterion-guided small organic nanoparticles. (a) Schematic illustration of the self-assembly process and the structure of SMILES materials (top). The cyanostar anion receptor (CS) that binds to the PF6 ion in a 2-to-1 anionic complex spatially and electronically isolates lipophilic rhodamine R12 dyes. The successful formation of SMILES NPs was indicated by a red-transparent and highly fluorescent aqueous solution, which is in contrast to the nonfluorescent control sample without cyanostar (bottom left). The Cryo-TEM micrograph SMILES NPs showing spherical morphology and broad size distribution (25 ± 8 nm) (bottom right).^158^ (b) Chemical structures of the donor dye, rhodamine B octadecyl ester (R18), its counterion, tetrakis(pentafluorophenyl)borate (F5-TPB), and the acceptor dye, Cy5 dye (DiD) (top left). Schematic representation of the giant light-harvesting nanoantenna (bottom left) inside polymer NPs (right). Short-range ordering of R18 cations (green) by the F5-TPB counterion (blue) inside the PMMA-MA matrix prevents dye aggregation at short interfluorophore distances, promoting ultrafast EET with subsequent FRET to a single acceptor molecule (red).^154^ (c) Bright fluorescent NPs with varied fluorescence wavelengths were obtained by a high-density loading of cationic cyanine dyes (left) and their counterions (rigth) into polymer NPs.^155^ (a) Reproduced with permission from ref (158), copyright 2021 Wiley-VCH Verlag GmbH & Co. (b) Reproduced with permission from ref (154), copyright 2017 Macmillan Publishers Ltd., part of Springer Nature. (c) Reproduced with permission from ref (155), copyright 2017 Wiley-VCH Verlag GmbH & Co.

Bulky hydrophobic counterions, generally weakly coordinating anions (e.g., perchlorate, tetraphenylborate, tetrakis(4-fluorophenyl)borate, and tetrakis(pentafluorophenyl)), may serve as a spacer that minimizes dye aggregation and self-quenching.^153^ These bulky conterions are characterized by their large size, high hydrophobicity, and strong delocalization of the negative charge over the large volume of the anion, and they can serve as spacers (or insulators) between cationic dyes. This allows dense packing with short dye–dye distances inside nanoparticles. In addition, such structures exhibit a giant light-harvesting behavior due to the efficient ultrafast excitation energy transfer (EET, <30 fs), enabling the detection of a single particle with ambient light (Figure 8b).^154^ Using this approach, fluorescent polymer nanoparticles with varied fluorescence wavelengths have been developed (Figure 8c).^155^ Counterion-assisted AIE has been reported using cationic styrylpyridinium dyes bearing diethylamino, diphenylamino, and carbazolyl groups. Bulky fluorinated tetraphenylborate counterions light up the NIR fluorescence (Φ_fl_ up to 40%) of poorly fluorescent dyes at 500 mM loading yield in polymeric nanoparticles.^156^

An alternative approach is based on hierarchical coassembly using a scaffold ensuring spatial and electronic isolation to prohibit fluorescence quenching in an aggregate state. This small-molecule ionic isolation lattices (SMILES) transfers the optical properties of typical cationic dyes directly and reliably from solution to high-density molecular crystals (Figure 8a).^157,158^ A cyanostar macrocycle that binds to hexafluorophosphate anions forms a cyanostar-anion complex, which acts as a binding platform to assemble cationic dyes and enforces charge-by-charge alternating packing to isolate the dye molecules. SMILES crystals are formed with most cationic dyes, including cyanines, oxazines, and rhodamines, which deliver predictable optical properties and high fluorescence brightness with high dye densities (one dye per ∼4 nm^3^).

## 4 Conjugated Polymer-Based Nanoparticles (Pdots)

### 4.1 Fluorescence Characteristics of Conjugated Polymers

Conjugated polymer nanoparticles (Pdots) are nanometer-size aggregates of conjugated polymer (CP) chains. CPs consist of a large number of “quasi chromophores”, and photophysical interactions between the chromophores along the chains often determine their fluorescence properties rather than fluorescence characteristics of each chromophore. Single-molecule fluorescence spectroscopy experiments on a poly(phenylenevinylene) derivative revealed that only a small number of chromophores emit fluorescence, although each CP chain contains more than a hundred chromophores.^159^ This finding was interpreted by an efficient excited-state energy funneling to a small number of chromophores.^160^ The efficiency of energy funneling and thus the number of chromophores and fluorescence brightness of CPs depend significantly on the conformational states of chromophores.^161−163^ In addition, a twist between monomers on donor–acceptor-type CPs has a large effect on their fluorescence characteristics.^150,164^ These findings have been extended to interchain interactions in aggregation states. Depending on their aggregation states (i.e., J-, H-, or random aggregates), CP aggregates that contain tens of CP chains showed fluorescence emission from a different number of the chromophores, in some cases only from a few chromophores.^165−167^ These findings indicate that the development of Pdots with bright fluorescence requires a proper molecular design of CPs as well as an optimization of interchain interactions inside the particles.

### 4.2 General Molecular Design Strategy for Obtaining Pdots with Bright Fluorescence

Pdots consist of tightly packed π-conjugated polymer chains with particle sizes ranging from a few to tens of nanometers. The photophysical properties are highly related to the nature of the constituent chromophores, backbone planarity, potentials for torsion and deflection of the backbone, surface charges, functional groups, and aggregation extent. In general, rigid planar π-conjugated structures show remarkably high optical absorption, explained by the high persistence length of the polymer,^168^ and strong emission as isolated species. The spectral properties of conjugated polymers can vary significantly with the band gap. The band gap depends on the degree of extended conjugation, which, in turn, should depend on the level of planarity of the polymer. Planar systems have better orbital overlap, which lowers the band gap. Thus, the rigid and coplanar structure is beneficial to enhance the absorption properties.

However, at the same time, the planarization may cause imperfect fluorescence properties in the Pdots, inducing stronger intra- and interchain interactions (e.g., π–π interactions) that promote the formation of nonemissive excimers and exciplexes (i.e., quenching sites, energy trap sites). The drop in Φ_fl_ is usually more pronounced in larger particles as the efficiency of energy transfer to various fluorescence quenching sites increases. However, ε increases with the particle size, which may lead to the increase in overall brightness. Aggregation-caused quenching of fluorescence becomes especially problematic in Pdots emitting in the NIR and SWIR spectra regions, since their extended π-conjugation system is often composed of large flat-shaped molecules that contribute to increased π–π interactions. To prevent the generation of fluorescence quenching sites, Pdots have often been fabricated by mixing CPs with nonfluorescent amphiphilic copolymers that maintain a physical separation of CP chains inside the particles. On the one hand, this approach effectively maintains large Φ_fl_ inside the particles, although the overall brightness of the particles significantly drops due to the smaller fraction of the fluorochromes inside the particles. On the other hand, blending with amphiphilic copolymers allows for the introduction of functional groups and eliminates nonspecific adsorption issues. Furthermore, the extent of inter- and intrachain interactions in Pdots can partially be controlled through the type of copolymer used and the condition on nanoparticle preparation.^169^

#### 4.2.1 D-A-type Polymers Based on Benzodifuran and Thienothiophene

The first low band gap conjugated polymer with intrinsic fluorescence in the SWIR spectral region was obtained through the alternating donor–acceptor structure based on furan and thiophene units (pDA).^170^ Higher electronegativity and smaller atomic size of oxygen make furan less aromatic, which leads to a greater contribution of the quinoidal resonance structure in polyfurans, resulting in furan-based π-conjugates with small torsional angles. Therefore, such materials have an improved conjugation with reduced π–π stacking.^171^ In addition, they do not suffer from the heavy-atom effect and thus exhibit stronger fluorescence. Nanoparticles with fluorescence in the range of 1050–1350 nm and Φ_fl_ = 1.7% in water (0.17% after correction, Φ_fl_ of IR26 = 0.05%) were obtained by mixing pDA with phospholipid–poly(ethylene glycol).

**Figure 9:**
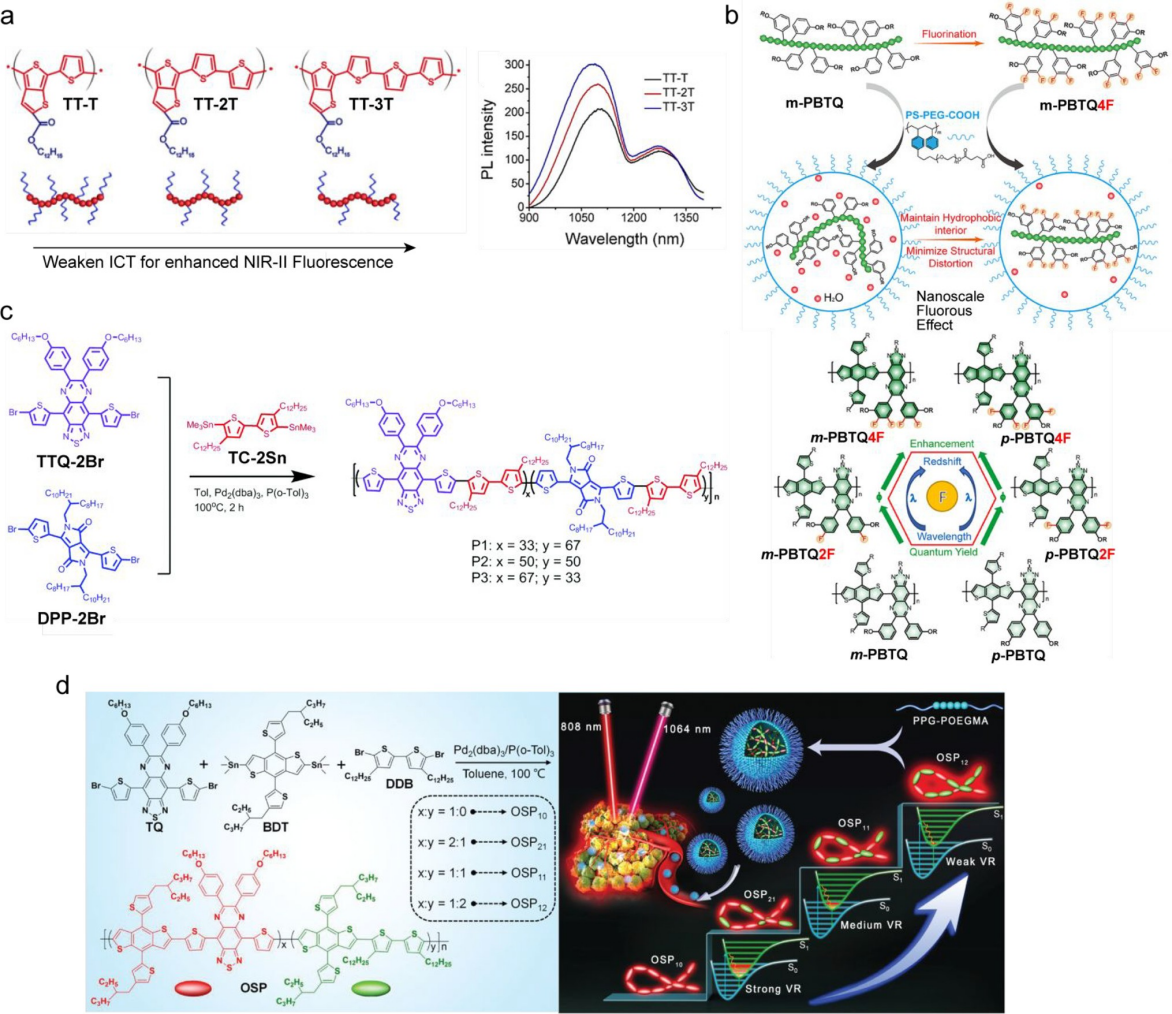
Molecular design strategy for obtaining Pdots with bright fluorescence. (a) Controlling the fluorescence brightness of Pdots in the SWIR range by varied lengths of thiophene units. Chemical structures of the conjugated polymers thienothiophene-thiophene (TT-T), thienothiophene-bithiophene (TT-2T), and thienothiophene-terthiophene (TT-3T) (left). Red region represents the quinoid polymer backbones, and the blue region represents the electron-withdrawing carboxylic acid dodecyl ester group. Absorption spectra and fluorescence emission spectra of the three conjugated polymers at the same concentration in THF (right).^173^ (b) Molecular design and fluorination of CPs. Schematic illustration describing a nanoscale fluorous effect to maintain the hydrophobic interior and minimize the structural distortion of CPs in Pdots (top). The fluorination red shifts the optical spectra and enhances the fluorescence quantum yield (bottom).^178^ (c) Double-acceptor CPs. Synthetic route and chemical structures of three double-acceptor conjugated polymers.^179^ (d) Improved fluorescence brightness by controlling intramolecular vibrational relaxation. Synthetic routes of CPs and OSPs, with various doping ratios of DDB (left). Schematic diagram of the OSPs with different doping ratios of DDB, indicating a gradually enhanced SWIR fluorescence of OSPs with the increasing doping ratio of DDB (right).^180^ (a) Reproduced with permission from ref (173), copyright 2019 American Chemical Society. (b) Reproduced with permission from ref (178), copyright 2020 Wiley-VCH Verlag GmbH & Co. (c) Adopted with permission from ref (179), copyright 2021 The Royal Society of Chemistry. (d) Reproduced with permission from ref (180), copyright 2021 Wiley-VCH GmbH.

Subsequently, another CP (PDFT) based on a planar diketopyrrolopyrrole moiety incorporating furan units was synthesized. PDFT coencapsulated with PEGylated phospholipid (DSPE-mPEG) leads to 68 nm PDFT1032 Pdots, with absorption and fluorescence peaks at 809 and 1032 nm, respectively. Combining the diketopyrrolopyrrole donor and the triazole [4,5-*g*]-quinoxaline (TTQ) acceptor leads to a new narrow band gap conjugated polymer (DPQ).^172^ DPQ coencapsulated with glycolysis inhibitor, 2DG, in liposomes showed absorption at 1064 nm and bright fluorescence emission peaking at 1300 nm. A series of quinoid polymers containing ester-substituted thieno [3,4-*b*] thiophene (TT) connected with a varied lengths of thiophene units (thiophene (T), bithiophene (2T), and terthiophene (3T)) were designed with the aim of controlling the fluorescence brightness in the SWIR range (Figure 9a).^173^ By increasing the length of the thiophene chain from TT-T to TT-3T, the density of the electron-withdrawing group decreased, leading to weaker ICT and thus brighter fluorescence. Among the Pdots consisting of these CPs and an amphiphilic copolymer Pluronic F-127, TT-3T Pdots exhibited a largest Φ_fl_ (Φ_fl_ = 1.75% in water, 0.33% after correction, Φ_fl_ of R1061 = 0.32% in DCE) because of the weaker ICT compared with others.

#### 4.2.2 Fluorination-Enhanced Fluorescence

A conformation and morphology of CP backbone can be modified by fluorination.^174^ In general, fluorination introduces higher degrees of main-chain planarity, rigidity order, and packing tightness. Thus, structural distortions between the excited and ground states are minimized by this substitution, which enhance the Φ_fl_ of Pdots.^170,175,176^ In addition, the fluorination of CPs affects their energy levels and intra- and intermolecular interactions.^177^ This strategy was recently applied to develop highly fluorescent CPs in the SWIR spectral window.^170,178^

The potential benefits of fluorine substitution have systematically been investigated by synthesizing a set of fluorine-substituted D-A polymers composed of benzodithiophene (BDT) as a donor and triazole[4,5-g]-quinoxaline (TQ) as an acceptor unit. The observed brighter fluorescence of the Pdots consisting of the fluorinated CPs was attributed to the nanoscale fluorous effect, a chain–chain segregation in the Pdots due to the fluorine–fluorine interactions through which unfavorable interactions with other elements can be avoided. Thus, the hydrophobicity and molecular planarity of the CPs were largely retained in the Pdots, suppressing the quenching by the interchain interactions (Figure 9b). Fluorine-substituted m-PBTQ4F Pdots exhibited red-shifted absorption (λ_max_ = 946 nm) and fluorescence (two major peaks at λ_max_ = 995 and 1123 nm), with Φ_fl_ = 3.2% (0.32% after correction, Φ_fl_ of IR26 = 0.05%). Similarly, a favorable effect of acceptor fluorination was observed in a series of far-red/NIR emissive D-A CPs that compromise of thiophene donor (T) and quinoxaline (Q) acceptor units.^169^ However, in contrast to previous studies, the observed enhancement in brightness of Pdots upon fluorination was assigned to the presence of unfused and less-rigid polycyclic monomer building blocks, which reduced the planarity of the CP chains in the Pdots through the anchoring of fluorine atoms.

#### 4.2.3 Double-Acceptor Polymers

An improved fluorescence of Pdots in the SWIR region has been reported by introducing additional units (either donor or acceptor) that expand a π-conjugation system. This was done by synthesizing a series of double-acceptor conjugated polymers consisting of the thiadiazolo [3,4-g] quinoxaline derivative (TTQ) and the pyrrolo [3,4-*c*] pyrrole-1,4-dione derivative (DPP) as acceptors and bis(trimethylstannane) (TC) as donor (Figure 9c).^179^ Pdots consisting of the double-acceptor CPs with varied ratios of the two acceptors (TTQ/DPP = 1:2, 1:1, and 2:1 in P1, P2, and P3) exhibited main absorption peaks at ∼700 nm, with corresponding fluorescence maxima at 1257–1272 nm. These Pdots showed Φ_fl_ in the range of 0.05–0.10% (0.009–0.019 after correction, Φ_fl_ of R1061 = 0.32% in DCE).

#### 4.2.4 Improved Fluorescence Brightness by Controlling Intramolecular Vibrational Relaxation

A remarkable amplification of fluorescence brightness in the SWIR region was achieved by the introduction of additional weak-electron-donating units (5,5′-dibromo-4,4′-didodecyl-2,2′-bithiophene, DDB) into the backbone of organic semiconducting polymers (OSPs), OSP_10_, which was composed of a strong benzo-dithiophene-based electron-donating unit (BDT) and a thiadiazolo-quinoxaline (TQ) strong electron-withdrawing unit (Figure 9d).^180^ A strong coupling between BDT and TQ is beneficial to narrow the band gap for the red-shifted absorption spectrum of the OSP_10_ CPs. Furthermore, by the introduction of DDB with controlled doping ratios, self-brightened OSPs (OSP_21_, OSP_11_, and OSP_12_) with gradient SWIR brightness were synthesized. The DDB doping induced a 6.3- to 25-fold fluorescence enhancement compared to the undoped counterpart (OSP_10_). A femtosecond transient absorption spectroscopy revealed that the DDB doping-induced suppression of vibrational relaxation is the major mechanism underlying the observed amplifications of the SWIR fluorescence.

### 4.3 Improving Fluorescence Brightness of Pdots through Chain Conformation

#### 4.3.1 Introduction of Twist in Polymer Backbone

**Figure 10:**
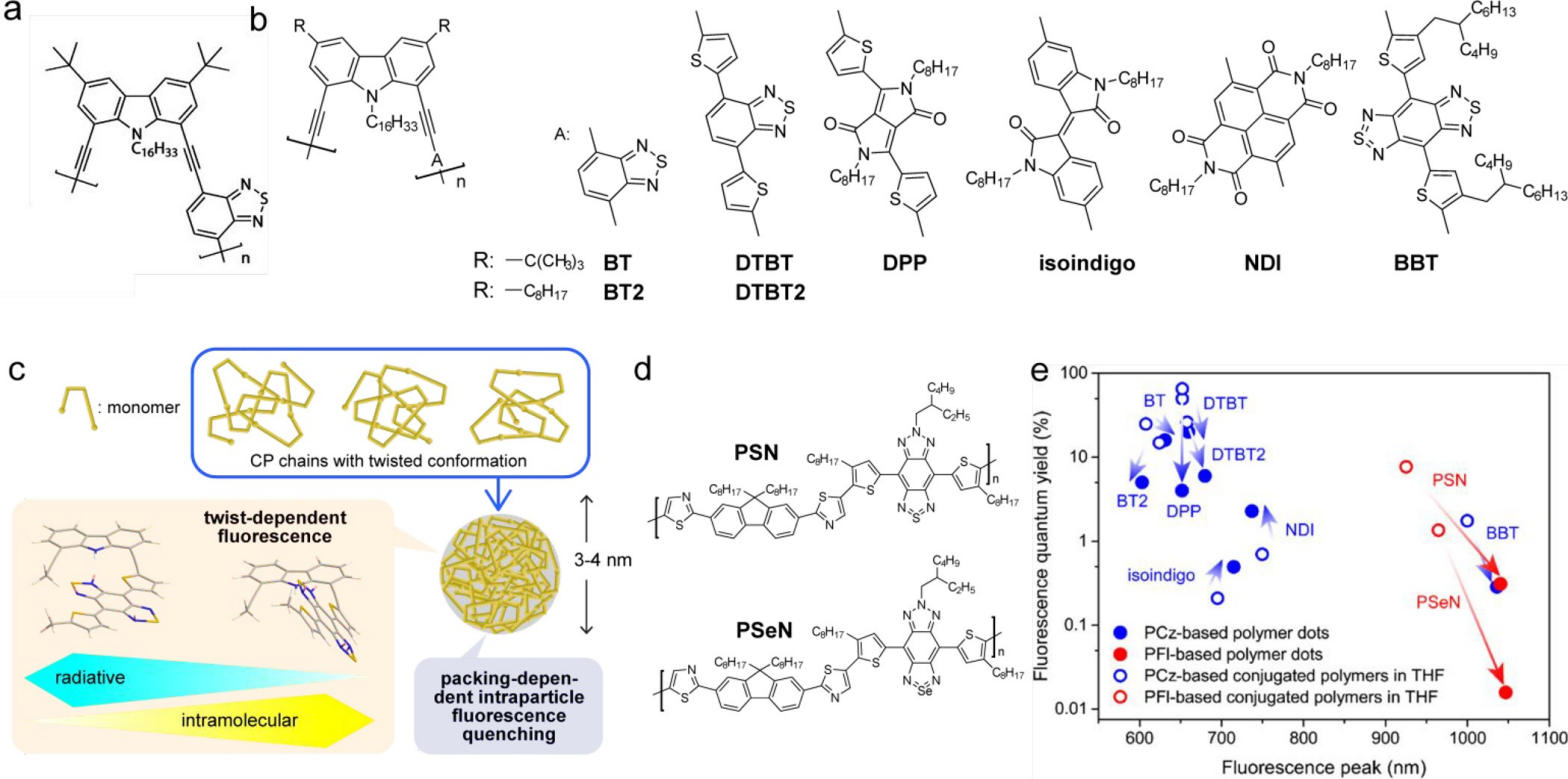
Improved fluorescence brightness of Pdots by introducing a twist in the polymer backbone. (a) Chemical structure of the CzBT CP. (b) Chemical structures of Cz-based D–A-type CPs with varied acceptor moieties (BT, DTBT, DPP, isoindigo, ND, and BBT). These polymers adopt a twisted conformation due to the steric hindrance between the donor and acceptor moieties caused by their bent shape. (c) Schematic illustration of the conformational state of the twisted polymer chains in solution and in Pdots.^181^ (d) Chemical structures of polyfluorene (PF)-based DA-type CPs that adopt a linear and planar shape (PSN and PSeN). (e) Φ_fl_ values of the Cz-based (blue) and PF-based (red) CPs in THF (open circles) and the Pdots dispersed in water (filled circles) plotted against their peak fluorescence wavelengths. Arrows highlight the change in Φ_fl_ and peak fluorescence wavelength upon the Pdots formation.^182^ (b, d, e) Reproduced with permission from ref (182), copyright 2020 American Chemical Society. (c) Reproduced with permission from refs (181 and 182) under CC BY 4.0 and copyright 2020 American Chemical Society.

A single-molecule photophysical study of a D-A-type conjugated polymer CzBT composed of an alkyne-linked 1,8-carbazole (Cz) and benzothiadiazole (BT) revealed unique intersegment interactions and a whole-chain conformation based on detailed spectroscopic characterization, including absorption and fluorescence spectra, fluorescence lifetime, and exciton migration that depend on the twist angle between donor and acceptor (Figure 10a).^150,164^ The unique design of twisted PCzBT CPs and its dithienylbenzothiadiazole analogue, CzDTBT CPs (Figure 10b), further allowed one to fabricate ultrasmall (3.0–4.5 nm) Pdots with suppressed interchain interactions due to their twisted conformation, resulting in excellent fluorescence brightness and great photostability (Figure 10c).^181^ In addition, the Φ_fl_ value of the Pdots increased by a factor of ca. 2 by controlling the polymer chain packing through the optimization of the precipitation protocol. The structural characterization pointed to the essential role played by the packing of the polymer chains in the Pdots on the intraparticle spatial alignment of the emitting sites, which regulate the fluorescence brightness and the intraparticle energy migration efficiency.

The effect of the twisted conformation of CPs on the fluorescence brightness of resulting Pdots has systematically been investigated by synthesizing a series of Cz-based D-A-type CPs with varied acceptor units (benzothiazole (BT), dithieno-benzothiadiazole (DTBT), diketopyrrole (DPP), isoindigo, naphthalene diimide (NDI), and benzobisthiadiazole (BBT)) (Figure 10b). The study revealed that the fluorescence brightness of the resulting Pdots is determined by a subtle balance between the fluorescence quenching caused by the polymer chain interaction inside the particles and the twisting between the donor and acceptor moieties of the conjugated polymers inside the particles.^182^ Importantly, most Cz-based polymer dots (BT, BT2, DTBT, DTBT2, and BBT) showed a relatively small reduction in Φ_fl_ (up to 6 times reduction) upon particle formation compared to polyfluorene-based CPs that adopt a linear and planar shape (poly(dithiazolfluorene-*alt*-thiadiazolobenzotriazole) (PSN) and poly(dithiazolfluorene-*alt*-selenadiazolobenzotriazole) (PSeN), 25–87-fold reduction) (Figure 10d,e). These results provide strong evidence that fluorescence quenching can partly be suppressed by the fabrication of Pdots using CPs that have a bent and twisted conformation.

#### 4.3.2 Polymer with Planar-Twist Conformation

**Figure 11:**
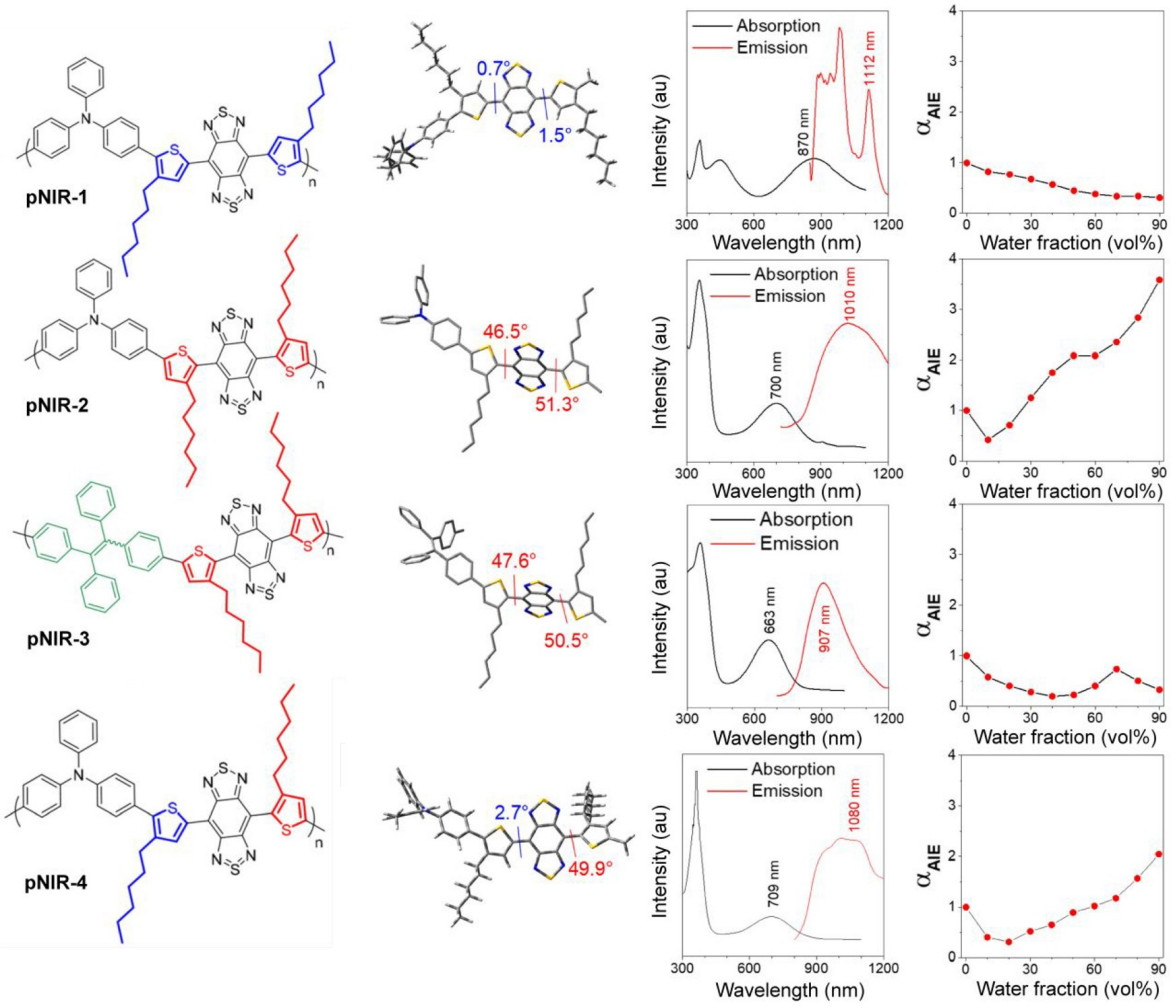
Improved fluorescence brightness of Pdots by introducing a planar-twist conformation in polymer backbone. Chemical structures of pNIR-1, pNIR-2, pNIR-3, and pNIR-4 polymers and their respective ground-state geometries with the distinct dihedral angles (left). Absorption and fluorescence spectra of the polymers in THF (center). Fluorescence intensity variation of the polymers in THF/H_2_O mixtures with varied water fraction (right). Reproduced with permission from 183, copyright 2020 American Chemical Society.

The effect of the twisted CP chains on the improvement of fluorescence brightness of Pdots has also been confirmed by synthesizing a series of D–A-type CPs with either triphenylamine (TPA) or tetraphenylenthylene (TPE)-based D units connected through an alkylthiophene linker (T) to the benzo[1,2-c:4,5-c′]bis([1,2,5]thiadiazole) (BBTD) acceptor (Figure 11).^183^ In this study, the magnitude of the chain twist was controlled by the dihedral angle between the D unit and the T linker group through the balanced steric hindrance of chain substituents. The Φ_fl_ values of Pdots composed of twisted pNIR-2 (Φ_fl_ = 3.2%, 0.32% after correction, Φ_fl_ of IR26 = 0.05%) was 1.45 times higher than in Pdots composed of partially planarized pNIR-4 (Φ_fl_ = 2.2%, 0.22% after correction, Φ_fl_ of IR26 = 0.05%). It was found that the overall fluorescence brightness of pNIR-4 was 20% larger than that of pNIR-2 because of the extended π-conjugation in pNIR-4, which led to larger ε value. The coplanar pNIR-1 displayed no fluorescence (Φ_fl_ = 0) because of the fluorescence quenching induced by interchain interactions. The lack of fluorescence in pNIR-1 might also be attributed to a very short length of the polymer chain in which π–π quenching could be pronounced.

#### 4.3.3 Anti-aggregation-Caused Quenching and Aggregation-Induced Emission

**Figure 12:**
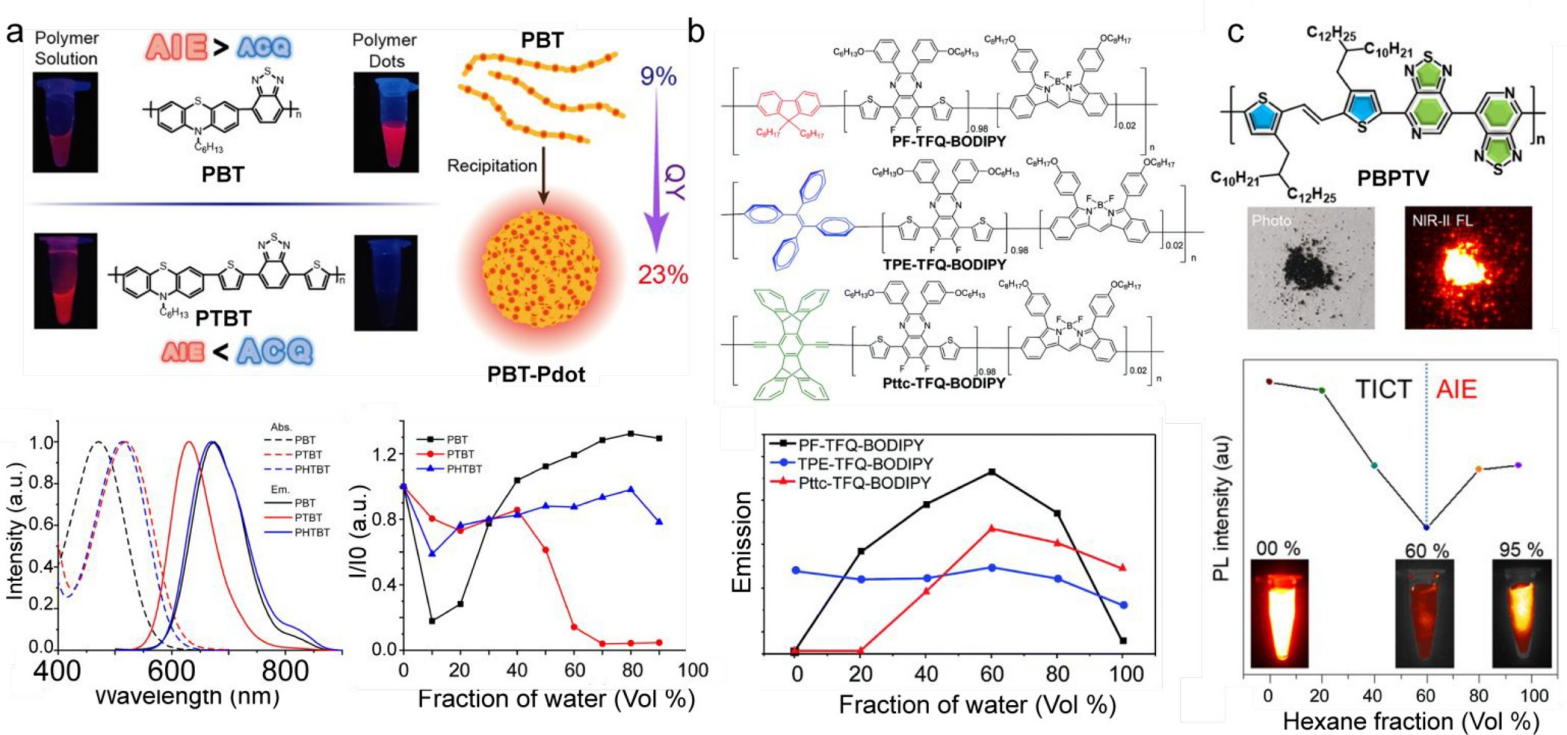
Improved fluorescence brightness of Pdots by antiaggregation-caused quenching and aggregation-induced emission. (a) Competition between the AIE and ACQ effects (top). The AIE polymer PBT demonstrated a remarkable increase in fluorescence intensity (QY increased from 9% in solution to 23% in Pdots). In contrast, the PTBT polymer displayed an intense ACQ effect, which resulted in low fluorescence emission after Pdot formation. Absorption and fluorescence spectra of the polymers PBT, PTBT, and PHTBT in THF (bottom left). Fluorescence intensity changes of PBT, PTBT, and PHTBT in THF/water mixtures with different water fractions (bottom right).^187^ (b) Chemical structures of three CPs with different donors; PF, dominated by the ACQ effect, TPE, AIE active fluorogen, and Pttc, a rigid three-dimensional moiety for anti-ACQ effect (top). Fluorescence intensities of the three CPs in mixed solvents of THF/water (water fraction, v/v%) (bottom).^186^ (c) Molecular structure of the PBPTV polymer. The inset shows bright-field and NIR-II fluorescence images of solid PBPTV (top). Fluorescence intensities of the CP in DCM/hexane mixtures with the change in the volume fractions of hexane (bottom).^184^ (a) Reproduced with permission from ref (187), copyright 2019 American Chemical Society. (b) Reproduced with permission from ref (186), copyright 2019 The Royal Society of Chemistry. (c) Reproduced with permission from ref (184), copyright 2020 American Chemical Society.

It is common that polymer nanoparticles exhibit reduced fluorescence compared to the former/constitutional polymers in organic solvents as a result of aggregation-caused quenching (ACQ). A CP possessing a twisted conformation PBPTV has been synthesized using a dual-acceptor CP based on a bispyridal [2,1,3]thiadiazole (BPT) unit as the acceptor and an alkyl-substituted (*E*)-2-(2-(thiophen-2-yl)vinyl)thiophene (TVT) unit as the donor (Figure 12c).^184^ Upon aggregate formation, the PBPTV chains adopt a geometry with tilted or twisted close contact similar to a herringbone-like structure.^185^ Such a backbone conformation enhanced the interchain organization in an aggregated state that minimized fluorescence quenching (Φ_fl_ = 8.6% in organic solvent, 0.86% after correction, to Φ_fl_ = 7.9% in water, 0.79% after correction), misleadingly referred to as the AIE effect. Pdots fabricated using a mixture of PBPTV and an amphiphilic polymer DSPE-PEG_2000_ coated by a natural killer cell membrane exhibited absorption and fluorescence peaks at ∼700 and 960 nm, respectively.

A direct comparison between anti-ACQ and AIE has been conducted by synthesizing CPs containing a BODIPY acceptor and donor moieties with contrary characteristics (AIE tetraphenylethene (TPE) donor or anti-ACQ pentiptycene (Pttc) donor) bridged by quinoxaline (TFQ). It was found that the anti-ACQ-based platform outperformed the AIE-based approaches (Figure 12b).^186^ The introduction of a bulky rigid H-shaped Pttc unit effectively suppressed ACQ in Pdots, resulting in over 7 times improvement in the Φ_fl_ as compared to that of a planar counterpart. The anti-ACQ Pdots exhibited 1.4 times higher fluorescence brightness than the AIE Pdots.

An AIE effect in Pdots has been investigated using CPs consisting of an AIEgen phenothiazine (PTZ) donor and varied acceptors (benzothiazole (BT), dithieno-BT (TBT), and hexyl-substituted DTBT (HTBT)) (Figure 12a).^187^ PTBT displayed an intense ACQ, resulting in a drop of Φ_fl_ from 15% to 9% upon the Pdots formation. PHTBT showed a reduced ACQ effect due to an extra alkyl chain attached at thiophene that weakens interchain interactions in the Pdots (Φ_fl_ changed from 10% to 12% upon the Pdots formation). PBT demonstrated a remarkable increase in Φ_fl_ from 9% to 23% upon the Pdot formation. A similar behavior was observed in other phenothiazine-based polymers, namely, tetraphenylethylene- and triphenylacrylonitrile-substituted polyphenothiazines,^188^ polycarbazoles, and polytriphenylamines.^189^

Recently, Pdots that exhibit both anti-ACQ and AIE effects have been reported. A synergetic anti-ACQ/AIE effect was investigated by synthesizing a series of CPs consisting of AIEgen PTZ conjugated to side groups with a different bulkiness (nonbulky aliphatic chain, bulky TPA, and highly bulky diphenylanthracene (DPA)).^190^ Electron-acceptor units were also inserted to the CPs to shift the fluorescence wavelength into the SWIR spectral region. While the AIE effect (i.e., fluorescence brightness change upon particle formation using the CP with nonbulky chains) was marginal (Φ_fl_ = 0.08% in THF, 0.008% after correction, Φ_fl_ = 0.1% in Pdots, 0.01% after correction), the synergetic anti-ACQ/AIE effect was pronounced when Pdots were fabricated using the CPs with highly bulky DPA anti-ACQ moieties (Φ_fl_ = 0.6% in THF, 0.06% after correction, Φ_fl_ = 1.7% in Pdots, 0.17% after correction).

### 4.4 Conjugated Polymers Bearing Fluorescent Dye

**Figure 13:**
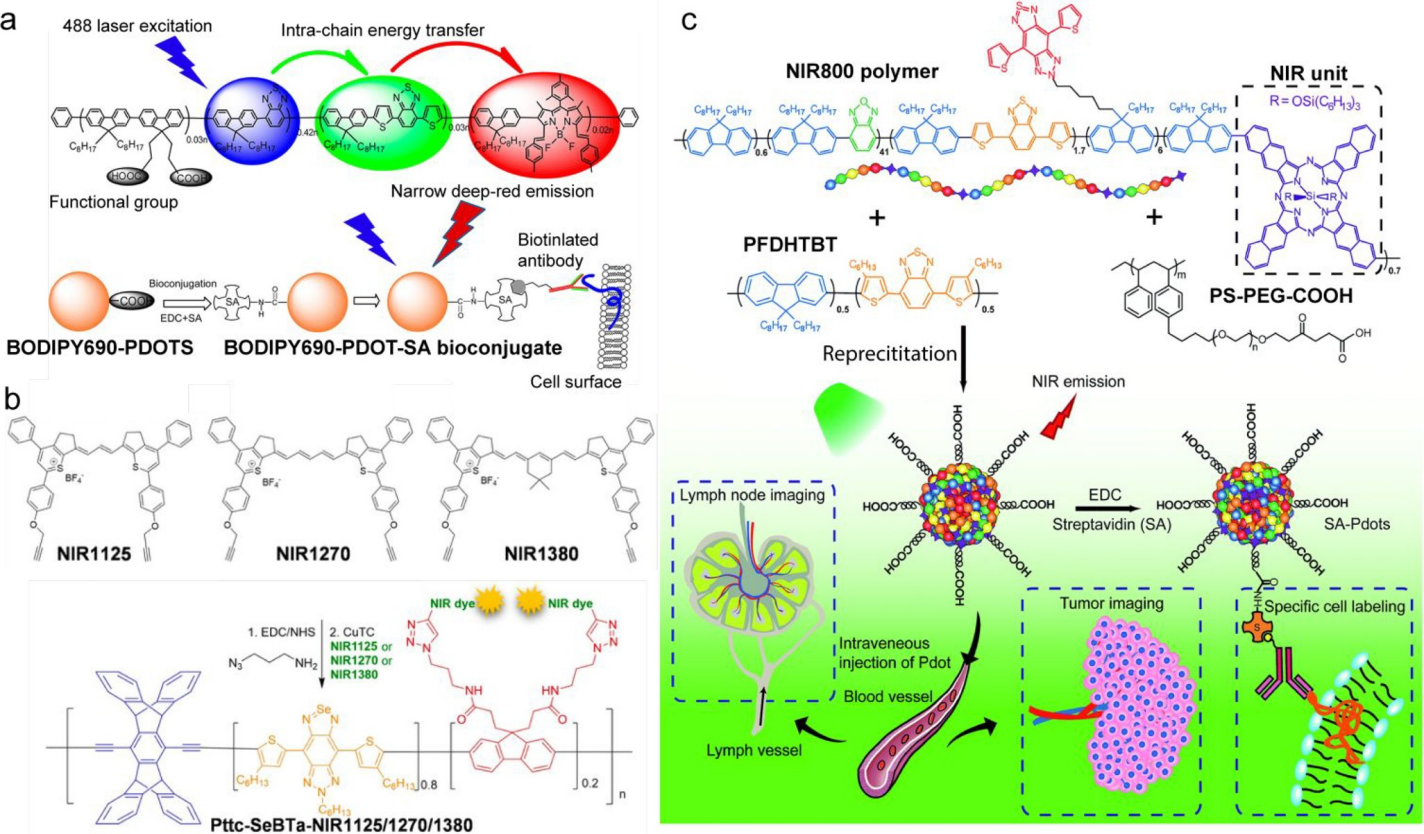
Improved fluorescence brightness of Pdots by incorporating fluorescent dyes into conjugated polymers. (a) Chemical structure and schematic illustration of cascade energy transfer in multicolor narrow emissive Pdots based on BODIPY units (top). Diagram of bioconjugation for specific cellular targeting (bottom).^191^ (b) Chemical structures of CPs conjugated to a polymethine dye. Three types of SWIR fluorescent polymethine dyes (NIR1125, NIR1270, and NIR1380) were used for conjugation.^195^ (c) Structure of the conjugated polymer backbone incorporating a porphyrin unit emitting in NIR (top). Schematic illustration of the preparation of NIR800 Pdots for a bioimaging application (bottom).^193^ (a) Adopted with permission from ref (191), copyright 2013 American Chemical Society. (b) Reproduced with permission from ref (195), copyright 2020 Wiley-VCH Verlag GmbH & Co. (c) Reproduced with permission from ref (193), copyright 2017 The Royal Society of Chemistry.

A variety of CPs bearing fluorescent dyes built in along the CP backbone have been reported. The covalent incorporation of fluorescent dyes overcame troublesome issues, including dye leaching and segregation, that could occur in a physical doping method. D–A-type CPs consisting of different BODIPY units as energy acceptors that emit sharp fluorescence at multiple wavelengths have been synthesized and used as precursors for preparing the Pdots (Figure 13a).^191^ On the basis of the same strategy, squaraine-based Pdots that show large Stokes shifts (∼340 nm) and narrow-band emissions in the NIR region (fwhm = 36 nm, which is ∼2 times narrower than those of inorganic quantum dots in the same wavelength region) with a large Φ_fl_ (Φ_fl_ = 0.3) have also been developed.^192^ A similar cascade energy transfer strategy has been used to design NIR emitting (peak fluorescence at 800 nm) NIR800 Pdots with relatively high Φ_fl_ (Φ_fl_ = 8%) and a narrow spectral bandwidth (fwhm ≈ 23 nm) (Figure 13c).^193^ A very efficient excitation energy migration/transfer from multiple donor units to a porphyrin acceptor unit was achieved by introducing these monomer units into one CP backbone.

SWIR emitting Pdots that consist of CPs bearing fluorescent dyes have been developed by incorporating a π-bridge selenyl benzothiadiazole (SeBTa), SWIR emitting polymethine dyes, NIR1125, NIR1270, or NIR1380, as an energy acceptor, and a bulky anti-ACQ unit, pentiptycene (Pttc) into one CP backbone (Figure 13b).^194^ Since the polymethine dyes were incorporated into pentiptycene selenyl benzothiadiazole (Pttc-SeBTa)-NIR1125/1270/1380 CPs by click reactions,^195^ this approach could be universal and could readily be utilized to integrate all kinds of fluorophores possessing reactive alkynes into CP chains.

## 5 NIR/SWIR Organic Probes in Imaging Applications

Fluorescence imaging of biological organisms at tissue, organ, and whole organism levels has become more important with an advance in our understanding of biological organisms at a system level. Advances in fluorescence microscopy techniques together with a recent development of new fluorophores with bright NIR/SWIR photoluminescence have been expanding the applicability of fluorescence-based bioimaging techniques. In this section, we introduce microscopy techniques used for deep-tissue imaging. Then we focus on single-particle (molecule) imaging in the SWIR spectral region and discuss factors affecting the SWIR imaging.

### 5.1 Fluorescence Microscopy Techniques for Deep Tissue Imaging with NIR/SWIR Emitting Fluorophores

#### 5.1.1 Single-Photon Excitation Microscopy and Related Approaches

Detecting fluorescence photons in the SWIR spectral range has proven to be the most effective one-photon approach for maximizing the imaging penetration depth. Despite the major achievements including real-time imaging of different pathologies such as vascular disorders, tumor angiogenesis, applications in image-guided surgeries, and background-free optical sensing, the main obstacles are the noise and scattering from tissues that reduce the precision of the method. To circumvent this and maximize the benefit of reduced photon scattering at long wavelengths, light-sheet microscopy with SWIR emitting inorganic nanoparticle using 1320 nm excitation light. The lowest degree of intensity decay and the least light-sheet thickness broadening were achieved using this method.^196^ An integration of structured illumination microscopy with the SWIR light-sheet microscopy with ultralong excitation and emission wavelengths up to ∼1540 and ∼1700 nm, respectively, lead to further diminished background and improved spatial resolution by approximately twofold.^197^ This mode of SWIR imaging allowed for great suppression of light scattering and large volumetric three-dimensional (3D) imaging of tissues with deep-axial penetration depths. A penetration depth up to ∼750 μm was achieved in the through-scalp and -skull imaging of intact mouse head without craniotomy^196^ and through-skin imaging of tumors.^197^ However, a spatial resolution below 1 μm at a high depth has not been achieved by these imaging techniques. This can be partially addressed by applying an image analysis using deep neural networks to transform a blurred image to a much higher-clarity image closely resembling the ground truth.^198,199^

#### 5.1.2 Two-Photon Excitation Microscopy

An alternative approach for improving the imaging depth and resolution includes intravital two-photon microscopy. Imaging with excitation in SWIR and detection in NIR was demonstrated with 35 nm size AIEdots composed of crab-shaped D–A-type dye molecules TQ-BPN possessing a planar thiadiazolo [3,4-g]quinoxaline-based core and several twisting phenyl/naphthyl rings to afford both a high fluorescence quantum yield and efficient two-photon activity.^200^ The in vivo two-photon fluorescence microscopy imaging of mouse brain using 1300 nm excitation through conical window allowed one to record 3D vascular information with superb spatial resolution (sub-3.5 μm) at white matter (>840 μm) and hippocampus (>960 μm) regions and detect small blood vessels of ∼5 μm as deep as 1065 μm in mouse brain. High-quality two-photon microscopy images were also acquired using 4 nm-size Pdots consisting of PIDTDBT CPs upon excitation at 800, 1040, and 1200 nm. Two-photon fluorescence microscopy imaging of blood vessels in mouse brain with a cranial window stained by the Pdots reached the depth of 1010 μm using 1200 nm fs laser excitation provided. Through-skull two-photon microscopy revealed blood vasculature architectures in bone marrow within the skull and beneath the skull with a high image contrast at the depth of 400 μm.^201^ Three-photon excitation microscopy with visible/NIR emitting fluorophores using 1550–1675 nm fs pulsed laser excitation^202,203^ provided vascular structures and neurons within intact mouse brain at a depth of 1000 μm.^202^

### 5.2 Single-Particle Fluorescence Imaging in NIR/SWIR Spectral Regions

The optical detection of single molecules occupies a central position of numerous biophysical studies, since it opens up the possibility of investigating individual molecular behavior usually hidden in ensemble measurements. Similar to the stepwise development of fluorescence imaging in the visible spectral range, the next important step in the SWIR fluorescence imaging would be single-molecule and single-particle-based studies. The key for a successful expansion of deep-tissue fluorescence imaging to single-particle detection is to optimize and extract a weak signal from a high background. Notwithstanding the many successes of single-molecule microscopy studies in the visible range, we face many challenges when we switch to fluorophores absorbing in the NIR spectral range with dim SWIR fluorescence. In general, the shift to longer wavelength improves the ensemble imaging capability by an efficient suppression of tissue autofluorescene. However, very low Φ_fl_ of SWIR emitters drastically limits single-molecule detection ability.

#### 5.2.1 Single-Particle SWIR Imaging with Nonorganic Fluorophores

Single-walled carbon nanotubes (SWCNTs) have a large size (sub-micrometer and micrometer lengths) and high rigidity with SWIR emission. By utilizing their relatively slow diffusion in biological tissues due to their large size and high rigidity, a video rate single-SWCNT imaging in the mouse brain has been reported. The small diameter of SWNTs gives accessibility to complex environments, allowing one to directly observe local extracellular space (ECS) structures and rheology in the mouse brain tissue.^204^ Because of the interplay between the nanotube geometry and the ECS local environment, the extract information on the dimensions and local viscosity of the ECS was also extracted. The brightness of SWNTs was improved by the introduction of sp^3^ defects, which enabled single SWNT imaging with high signal-to-noise ratios using unprecedentedly low excitation intensities (100 W cm^–2^), an order of magnitude lower than previously reported.^205^ In vivo mouse brain vasculature imaging with SWIR emitting indium-arsenide-based quantum dots (QDs) allowed single-particle image velocimetry with high spatiotemporal resolution.^206^ Detailed three-dimensional quantitative flow maps in the mouse brain were demonstrated and allow the study of ischemia–reperfusion in stroke. Other examples of SWIR single-particle microscopy include single-photon counting from individual single lead sulfide nanocrystals,^207^ shell thickness dependence studies of blinking behavior and Auger recombination in core/shell InP/CdS nanocrystals,^208^ lifetime dynamics in individual InAs QDs,^209^ photophysics of PbSe/CdSe/CdSe QDs,^210^ single-particle line width emission from InAs/CdSe,^211^ and DNA-stabilized silver nanocluster (DNA-AgNC).^212^

#### 5.2.2 Single-Particle NIR Imaging with Organic Fluorophores

The first single-molecule imaging with NIR emitting dyes was conducted using NIR emitting dyes IRdye 700DX and IRDye 800CW. Single-molecule images of MCF-7 cells labeled with these NIR dyes were captured by using epi-fluorescence microscopy and total internal reflection fluorescence microscopy.^213^ A visualization of NIR emitting single organic nanoparticles in vivo was demonstrated using octadecyl rhodamine B dye loaded with the assist of bulky hydrophobic counterion (perfluorinated tetraphenylborate) in poly(methyl methacrylate)-sulfonate nanoparticles (PMMA-SO_3_H) as a fluorophore insulator.^214^ These nanoparticles were further applied in live imaging of mouse brain by two-photon excitation at 780 nm. The bright fluorescence emitted by these particles allowed for the separation of individual particles, allowing one to track their trajectories in the blood vessels and acquire images with a high signal-to-noise ratio up to 800 μm depth.

#### 5.2.3 Single-Particle SWIR Imaging with Organic Fluorophores

**Figure 14:**
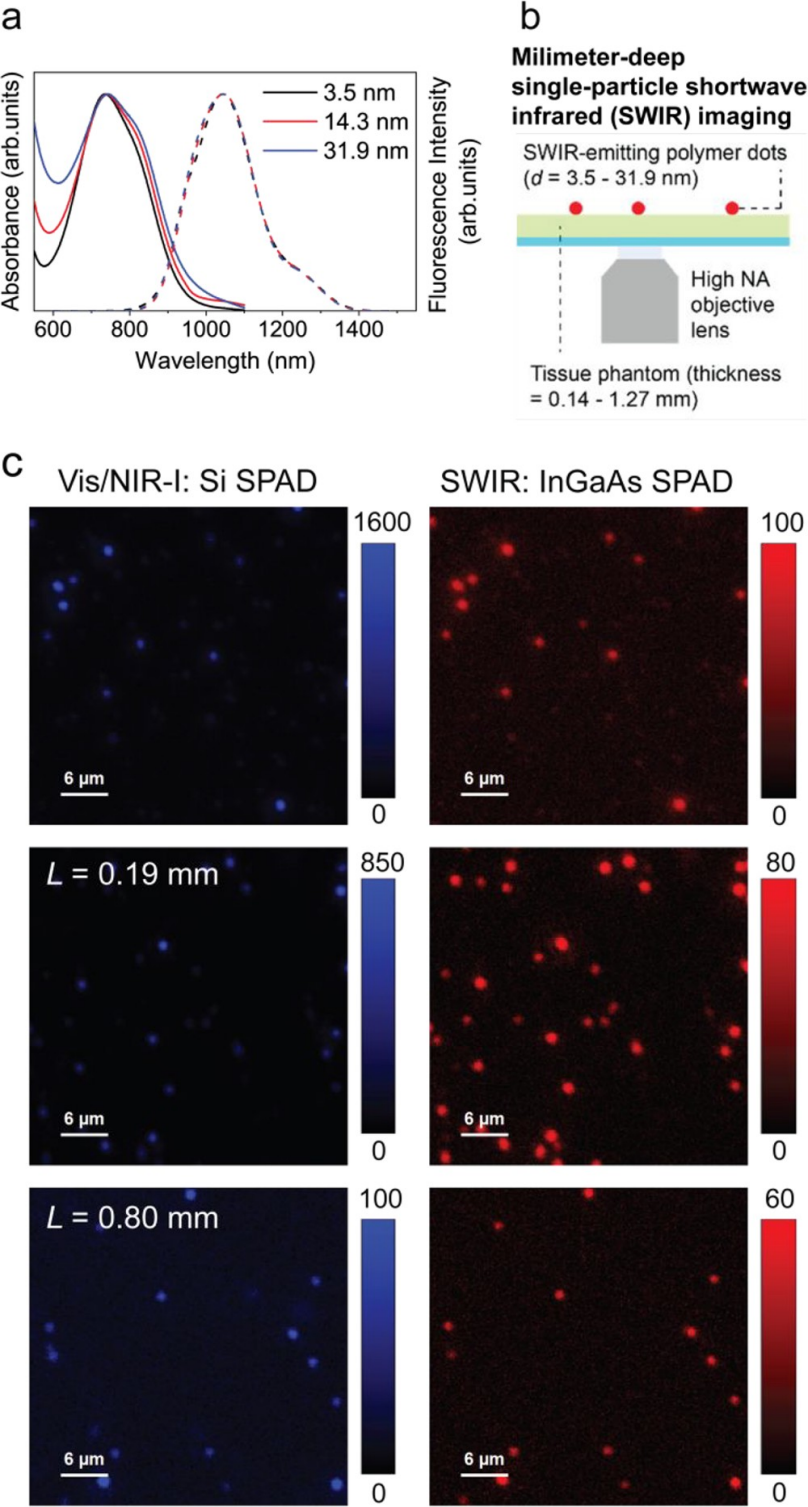
Single-particle SWIR imaging with Pdots (a) Steady-state absorption (solid lines) and fluorescence (dashed lines) spectra of PSN Pdots with three different sizes dispersed in water. (b) Schematic illustration describing the experimental configuration of single-particle fluorescence imaging of the PSN Pdots through tissue phantoms. (c) Fluorescence images of individual PSN Pdots deposited onto the tissue phantoms with varied thickness, as captured by the visible/NIR-I detector (left) and the SWIR detector (right).^215^ Reproduced with permission from ref (215), copyright 2020 American Chemical Society.

Single-particle SWIR fluorescence imaging with one-photon excitation has recently been demonstrated using SWIR emitting Pdots consisting of PSN CP (Figure 14).^215^ The fluorescence brightness of the PSN Pdots was adjusted by controlling the size of nanoparticles formulated in the nanoprecipitation process with an adjusted ratio of the organic to water phases. PSN Pdots with diameters of 3.5, 14.3, and 31.9 nm were obtained by this protocol. Photophysical characterization demonstrated that Φ_fl_ was independent of the particle size, whereas the fluorescence brightness under microscopy conditions increased gradually with the size of Pdots. This was primarily attributed to their large absorption cross section and high fluorescence saturation intensity. The very bright SWIR fluorescence of the PSN Pdots enabled single-particle imaging through tissue phantoms that mimic optical properties of biological tissues. A detection of the Pdots through a millimeter-thick phantom was demonstrated using Pdots as small as 14 nm.

### 5.3 Factors Affecting Single-Particle SWIR Fluorescence Imaging

#### 5.3.1 Fluorescence Brightness and Particle Size

Among many factors involved in design of bioimaging probes, the size (shape and surface coating chemistry) plays a key role in modulating interactions with a variety of physiological barriers present in living organism differentiating their excretion mechanism. Nanoparticles that are less than 6 nm are known to be readily eliminated through the kidneys by crossing a glomerular filtration barrier. The CDIR2 probe was the first SWIR fluorogen used for a real-time evaluation of renal function in living animals. After it is administered to mice, it passes through the glomeruli filtration system into urine without being reabsorbed or secreted in renal tubules. This unilateral renal clearance pathway allowed one to monitor in real-time the kidney dysfunction during the progression of renal impairment.^216^ In the case of larger particles, uptake of the reticuloendothelial system is the most common pathway for excretion. Nanoparticles will accumulate in the liver if their sizes are comparable to the size of vascular fenestration of the liver (50–100 nm) or end up in the spleen if their sizes are close to interendothelial cell slits of the spleen (100–200 nm).^217^ However, a recent study demonstrated that bone could also be an important organ for the retention of small nanoparticles.^218^ Systemically administered polymer nanoparticles with a diameter of 15 nm show significant accumulation and retention in the bone marrow, while nanoparticles larger than 25 nm were distributed primarily in the liver and spleen.

Organic-based SWIR emitting nanoparticles allow us to generate bright fluorophores with controllable size without affecting desired emission properties. In addition, the fluorescence brightness of organic nanoparticles often correlate positively with the size of these particles. Thus, depending on the size and brightness of the particle required in each imaging experiment, one can, in principle, tailor nanoparticles suitable for the application.

#### 5.3.2 Fluorescence Intensity Saturation

Single-molecule or particle fluorescence imaging normally requires a high excitation power to detect enough numbers of photons from individual molecules and particles. The key physical effect that limits the maximum emission rate from a single emitter at a one-photon excitation condition is the optical saturation of the transition. At a low incident laser power, the excited-state population and fluorescence signal are linearly proportional to the excitation intensity. As the laser power increases, the photon emission rate also increases linearly, as long as the optical transition is not saturated. At the point where the photon absorption rates are comparable to the spontaneous relaxation rate, the excited-state population will no longer increase linearly with increases in the excitation intensity due to the saturation. When the saturation occurs, a further increase of the laser power generates more background photons rather than fluorescence.

**Figure 15:**
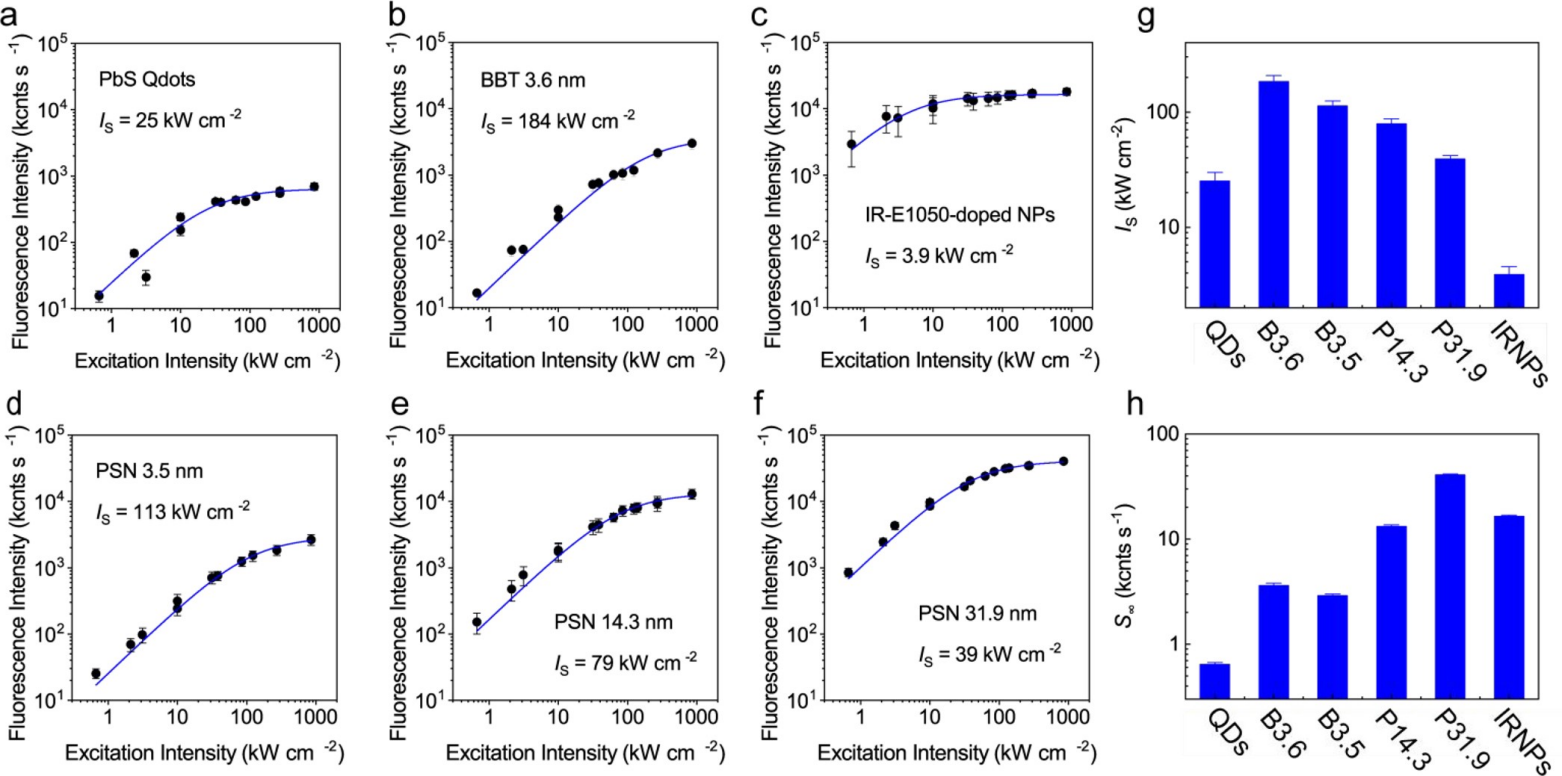
Fluorescence intensity saturation in single-particle SWIR fluorescence imaging. Excitation power dependence of the fluorescence intensity obtained for individual (a) PbS QDs (QDs), (b) 3.6 nm BBT Pdots (B3.6), (c) IR-E1050 dye-doped NPs (IRNPs), (d) 3.5 nm PSN Pdots (P3.5), (e) 14.3 nm PSN Pdots (P14.3), and (f) 31.9 nm PSN Pdots (P31.9) deposited onto a glass coverslip. The solid lines show fitting of the data to a two-state model. (g) Saturation intensities and (h) maximum fluorescence count rates obtained for the SWIR-emitting fluorophores determined by fitting the excitation power-dependent fluorescence intensity displayed in (a–f).^215^ Reproduced with permission from ref (215), copyright 2020 American Chemical Society.

The saturation intensity is directly related to the excited-state lifetime of the emitter. Fluorophores with a longer excited-state lifetime exhibit lower saturation intensity and, thus, a lower maximum photoluminescent count rate. On the one hand, SWIR emitting inorganic nanoparticles (PbS QDs) exhibit relatively low saturation intensities due to the long excited-state lifetime (in the order of microseconds). On the other hand, SWIR emitting Pdots (PSN and BBT) with short excited-state lifetimes (in the order of sub-nanosecond) showed higher saturation intensities (Figure 15).^215^ The difference in the saturation intensities directly translates into different maximum fluorescence count rates. Since many inorganic SWIR emitting fluorophores (e.g., semiconductor quantum dots, rare earth-doped nanoparticles (RENPs)) have long excited-state lifetimes (in the range of microsecond to millisecond), their application to single-particle SWIR imaging could have a limitation. In addition, the fluorescence rate per molecule at the saturation level is additionally affected by the population of the dark states once saturation is reached.

#### 5.3.3 Time-Gated Imaging

The quality of fluorescence images obtained from biological tissues can be dramatically improved by suppressing the light-scattering effect. Thus, a time-gated detection of the fluorescence signal is an effective approach for obtaining fluorescence images of biological specimens with minimal background signal. Time-gated fluorescence imaging has widely been implemented using fluorophores with very long excited-state lifetimes (e.g., RENPs). Although milliseconds time-gated imaging using RENPs is effective in an ensemble measurement, a low repetition rate of the excitation pulse greatly restricts the number of fluorescence photons detected from individual particles. Thus, this mode of time-gated imaging would be ineffective for single-particle imaging.

**Figure 16:**
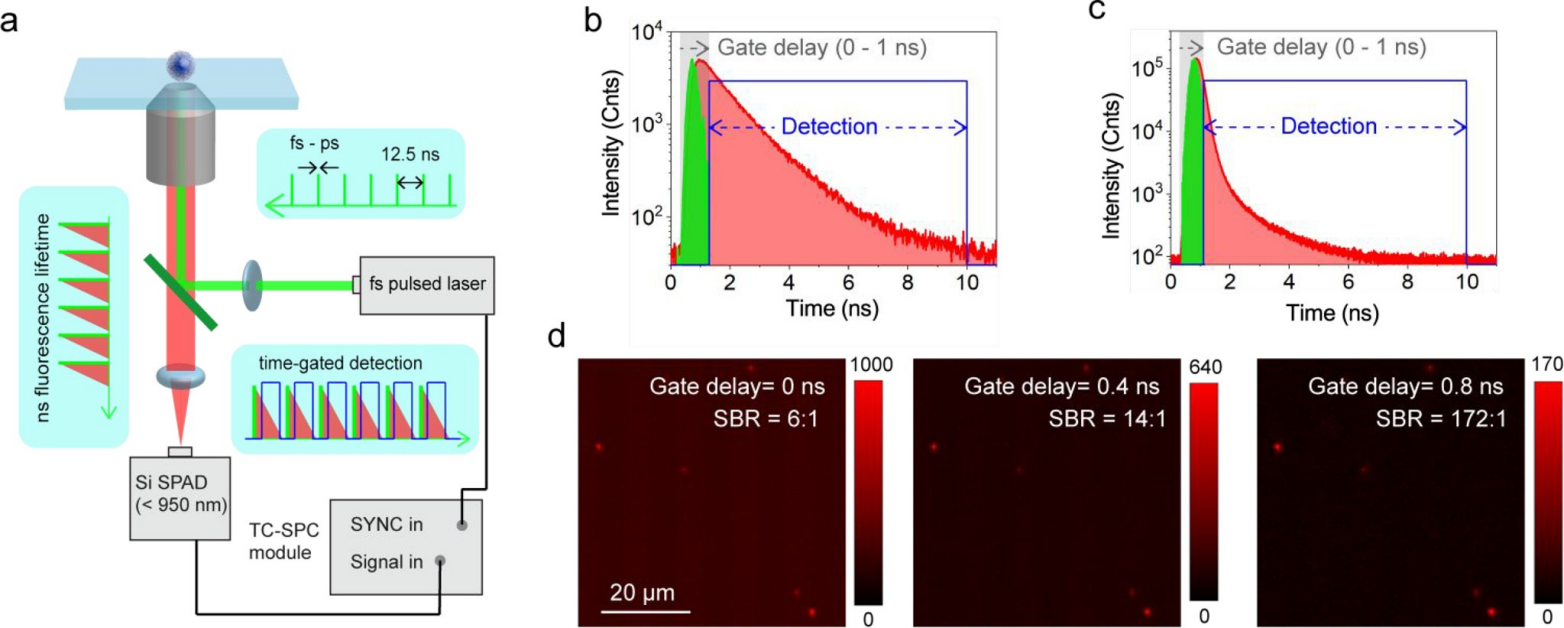
Single-particle time-gated SWIR fluorescence imaging. (a) Schematic illustration describing the basic principle of time-gated detection. (b, c) Fluorescence decay curves of the (b) NPs doped with IR-E1050 dye and (c) PSN Pdots (red) with the instrument response function (green). The gray-shaded regions correspond to the applied time delay of the detection. The blue arrows show the applied time gate of the detection. Fluorescence images of individual (d) PSN Pdots deposited onto a 40-μm-thick tissue phantom captured at varied time gates with signal-to-background ratios at applied time gate depicted.^215^ Reproduced with permission from ref (215), copyright 2020 American Chemical Society.

The short (sub)nanosecond-scale excited-state lifetime of organic fluorophores offers an alternative mode of time-gated imaging; that is, it used a high repetition rate pulsed laser and detected the SWIR fluorescence emission by applying a sub-nanosecond time gate. Using this mode of time-gated imaging, a significant improvement in signal-to-background-ratio (29× improvement of SBR) has been observed for the PSN Pdots (Figure 16).^215^ The improvement of SBR was achieved without substantially affecting the detection efficiency of the emitted fluorescence photon, which is the major disadvantage in afterglow fluorescence imaging, where the improved SBR is achieved at the expense of fluorescence brightness. This imaging mode is applicable to fluorophores with substantially short excited-state lifetimes (nanosecond range). Many inorganic SWIR-emitting fluorophores (e.g., RENPs and QDs) are not compatible because their excited-state lifetimes are on the order of microseconds to milliseconds.

## 6 Summary and Future Outlook

To date, organic SWIR probes have shown great potential for visualization and studies of physiological processes that take place in living organisms, including tumor detection, brain vasculature studies, tumor microenvironment sensing, and stem cell-based regenerative medicine. As we showed in this article, enormous activity has been focused on the development of various novel organic SWIR agents. From the bioimaging application point of view, in particular, potential single-molecule-based imaging applications, it is crucial to yield probes with superior brightness and photostability. Rational molecular engineering of fluorophores based on a deep understanding of their working mechanism is imperative.

**Figure 17:**
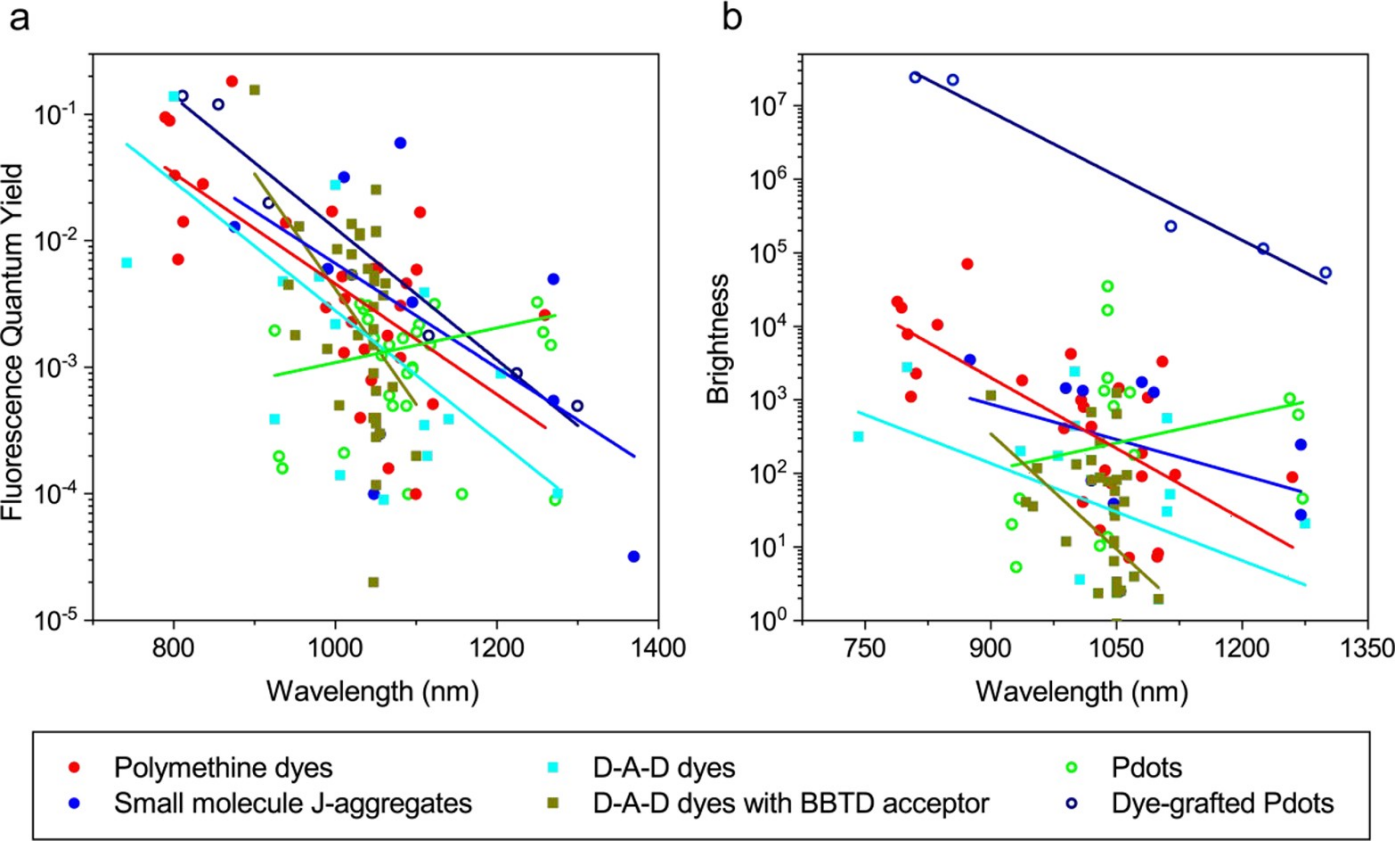
Fluorescence brightness of NIR/SWIR emitting organic fluorophores. Experimentally observed (a) Φ_fl_ and (b) fluorescence brightness (ε × Φ_fl_) as a function of the peak fluorescence wavelength of different classes of NIR/SWIR emitting organic fluorophores.

We performed a Φ_fl_ unification by using the reestablished most reliable Φ_fl_ values of fluorescence standards (see the Supporting Information: Reevaluation of fluorescence quantum yield standards in SWIR spectral region). On the basis of this data set, we constructed a wavelength-dependent Φ_fl_ distribution of reported dyes, together with a wavelength-dependent fluorescence brightness distribution (Figure 17). Figure 17 shows that there is an obvious limitation in obtaining organic dyes and nanoparticles that have a peak fluorescence emission above 1200 nm with a decent fluorescence brightness because of the energy-gap law. Currently, only a handful of organic fluorophores that exhibit peak fluorescence emission above 1200 nm with decent Φ_fl_ values are available. For instance, the peak fluorescence of the D-A-D molecules that incorporate the BBTD acceptor moiety does not exceed 1050 nm, although these molecules exhibit a wide variation of the Φ_fl_ values because of the antiquenching mechanisms introduced into each molecule. Because of the broad emission spectra of organic SWIR fluorophores, the tail-emission fluorescence often extends to the entire SWIR spectral range beyond 1500 nm. This tail fluorescence emission sometimes allows high-contrast imaging with current organic SWIR fluorophores.^29,34,96,219,220^ One of the important areas of the future development of the SWIR imaging technique to expand its applications would be a development of new organic fluorophores with relatively large Φ_fl_ values that exhibit peak fluorescence wavelength greater than 1200 nm.

The polymethine dyes, D-A-D dyes, and dye-grafted CPs exhibit a similar wavelength-dependent Φ_fl_ (Figure 17a). The polymethine dyes and polymethine dye-grafted CPs maintain a moderate Φ_fl_ at longer wavelengths compared with the D-A-D dyes. An incorporation of polymethine dyes into the nanoparticles could thus be one way to develop SWIR fluorophores that have a relatively large Φ_fl_ with a peak fluorescence wavelength greater than 1200 nm. The introduction of the twist to D-A-type molecules or to the backbone of CPs helps to preserve fluorescence brightness by minimizing the π–π quenching between the molecules and polymer chains in the nanoparticles. However, the introduction of twist into the D-A-D system may affect the conjugation system responsible for maintaining a large ε. Preserving the coplanar structure of the conjugated system and introducing shielding motifs by covalently attaching alkylene straps (which prevents from π–π stacking, decreases energetic disorder, and leads to a dramatic increase in backbone collinearity)^221,222^ or rigid three-dimensional pentiptycene (which prevent π-stacking or excimer formation),^223,224^ may become a new method to improve the overall fluorescence brightness.^225,226^ Interestingly, Pdots revealed a trend opposite to that of the polymethine dyes, D-A-D dyes, and dye-grafted CPs (i.e., larger Φ_fl_ at longer peak wavelength). This indicates that long conjugated D–A systems may help in the development of fluorophores with a reasonable Φ_fl_ at a peak fluorescence wavelength greater than 1200 nm.

In order to maximize the fluorescence emission rate, it is necessary to optically excite the molecule with a high probability and to have the largest possible fluorescence Φ_fl_. However, in the SWIR spectral range, Φ_fl_ is a major bottleneck (Φ_fl_ < 1%); thus, to obtain a sufficient fluorescence emission rate, fluorophores must possess a large ε. The importance of absorption is clearly seen when the fluorescence brightness (that is proportional to the product of ε and Φ_fl_) is plotted as a function of the fluorescence wavelength (Figure 17b). While the effect of the particle size or molecular weight of the fluorophores is not normalized in the plot displayed in Figure 17b, this plot provides valuable information about the development of brighter fluorophores. Pdots consisting of dye-grafted CPs show a larger fluorescence brightness compared with other SWIR probes. A large light-harvesting effect of the CPs and relatively high Φ_fl_ values of the grafted dyes enable one to obtain a very high overall fluorescence brightness. In addition, the CPs serve as a matrix that spatially isolates the grafted dyes and provides hydrophobic protection to the grafted dyes,^227^ which prevent fluorescence quenching of the grafted dyes. An optimization of the efficiency of energy transfer between the CPs and the grafted dyes, which requires a proper spatial arrangement of the two components, would be the key for the future development of this type of SWIR emitting organic fluorophores.

Nanoparticles consisting of J-aggregates of organic dyes are clearly one of the promising candidates to extend peak fluorescence wavelength beyond 1200 nm and enhance peak ε by spectral narrowing. Most of the J-aggregate nanoparticles reported so far are composed of polymethine dyes. However, J-aggregates of polymethine dyes exhibit dim monomeric fluorescence, which is further cut when aggregated. Therefore, there is an urgent need to develop alternative structures that have a high Φ_fl_ in the monomer state, which is not reduced by J-aggregate formation in an aqueous environment. One such example is a recently discovered D-A-D system that consists of a carbazole (Cz) donor and a benzothiadiazide (BT) acceptor bridged by an alkyne linker (CzBTCz) whose skeleton structure mimics cyanine dyes.^109^ The CzBTCz dye allowed producing 3.5 nm size J-aggregate nanoparticles that emit fluorescence in the visible wavelength region with Φ_fl_ close to unity. D-A-D molecules with a similar shape and extended π-conjugation system (e.g., replacing the acceptor unit) may push the spectral range of the bright J-aggregate nanoparticles to the SWIR spectral region.

One of the promising directions would be hybrid organic–inorganic nanocomposite SWIR probes that combine the merits of both components — the large ε of organic materials and the large Φ_fl_ of inorganic semiconducting materials. The high durability of such systems has been demonstrated in J-aggregate-based photosensibilization and photoluminescence amplification in SWNTs and NIR emitting PbS quantum dots,^228,229^ organic dye-coated RENPs providing a tunable excitation, and increased SWIR luminescence with up to 40-fold enhancement.^230,231^ The biocompatibility would remain a key issue for the development of hybrid organic–inorganic nanocomposites. In addition, a key issue for the development of hybrid nanocomposites will be the complexity of such a hybrid system. A proper nanoscopic spatial arrangement of each component within the hybrid nanoparticles would be essential to achieve functional SWIR-emitting nanoparticles because each component is responsible for light absorption and emission, respectively, and thus excited-state interactions between each component (e.g., energy transfer, electron transfer, etc.), which is sensitive to the spatial arrangement of each component, must be carefully controlled. Given the importance of uniformity, quality, and reproducibility of the fabrication of the hybrid organic–inorganic nanocomposites for imaging applications, these are the challenges that must be overcome for the practical use of the hybrid nanoparticles. Further development of organic SWIR emitting fluorophores and their integration into hybrid nanocomposites would greatly advance SWIR fluorescence imaging and its bioimaging applications.

## Supporting Information Available

Definition of J-aggregate, definition of aggregation-induced emission, and reevaluation of fluorescence quantum yield standards in SWIR spectral region, table summarizing spectroscopic characteristics of SWIR emitting fluorophores (PDF)
