# Supplementary material for: The Pursuit of Shortwave Infrared-Emitting Nanoparticles with Bright Fluorescence through Molecular Design and Excited-State Engineering of Molecular Aggregates
Source: ACS Nanosci Au. 2022 Feb 21;2(4):253–83. doi: 10.1021/acsnanoscienceau.1c00038 (PMC10125152; doi:10.1021/acsnanoscienceau.1c00038)
Supplement: Supplementary file 1 — ng1c00038_si_001.pdf [file ng1c00038_si_001.pdf]

Supporting Information for

The Pursuit of Shortwave Infrared-Emitting Nanoparticles with Bright Fluorescence through Molecular Design and Excited-State Engineering of Molecular Aggregates

Hubert Piwoński,* Shuho Nozue, and Satoshi Habuchi*

King Abdullah University of Science and Technology (KAUST), Biological and Environmental Science and Engineering Division, Thuwal 23955-6900, Saudi Arabia

* Corresponding should be addressed to Satoshi Habuchi (satoshi.habuchi@kaust.edu.sa) and Hubert Piwoński (hubert.piwonski@kaust.edu.sa)

Supporting Text

Definition of J-aggregates

Despite years of study and a number of scientific reports devoted to mechanisms of self-assembly and structure-related photophysics of J-aggregates, many recent papers that claimed the formation of J-aggregates in fluorescent nanoparticles used observed red shift of the fluorescence upon aggregation as an evidence of J-aggregates formation. According to the theory, the formation of J-aggregates is characterized by increased radiative rate (superradiant emission) that can results in enhanced quantum yield and reduced fluorescence lifetime and an appearance of a new red shifted narrow 'J-band'. The red shift of fluorescence emission is not the evidence for J-aggregates formation, which could be attributed to an increase in charge transfer (CT) character due to a geometry change, reduction in the band gap between the ground and excited states due to the enhanced van der Waals interactions, hydrogen bonding, and so on. In fact, in some cases, the red shift of the fluorescence maximum can be observed in aggregates with the H-type arrangement[1]. Moreover, the twisted charge-transfer π-conjugated systems can show blue-shifted emission upon J-aggregation[2]. Therefore, assigning red shifted fluorescence spectra as J-aggregates without analyzing absorption spectral narrowing and change in the radiative rate constant is misleading.

Definition of aggregation-induced emission

Most of new SWIR dye designs are based on the insertion of propeller shaped free-rotating units (e.g. TPA, TPE) into the molecule framework. This is inspired by the fascinating feature of aggregation-induced emission (AIE)-dyes where free-rotating units cause fluorescence quenching of monomeric dyes in an organic solvent through the opening of alternative/dominant nonradiative channels that depopulate excited state. By hindering the motions of free-rotating units in the aggregate state (RIR mechanisms), the appearance or enhancement of fluorescence emission is observed (AIE, AIEE effects).

We found that some recent articles reported new SWIR-emitting AIEgens based on the introduction of rotating units into fluorescent molecules, where such a new fluorophore possesses decent fluorescence in an organic solvent that is quenched upon aggregation in a water environment (e.g. Figure 12c). The observed quenched fluorescence in aggregated forms were called AIE because of the 1) introduction of well-known AIEgen generating groups and 2) observation of partial recovery of fluorescence at different organic-to-solvent ratios could be attributed to the suppression of TICT. Since the term AIE has a connotation of fluorescence enhancement, describing the partial recovery of the fluorescence intensity in the aggregate states as AIE is a misuse for the term even the partial recovery of the fluorescence intensity could be interpreted by the frozen motion of the TPA/TPE units and the inhibition of π-π stacking due to these bulky groups. The partial recovery of the fluorescence upon aggregation formation should be described as anti-quenching effect.

Reevaluation of fluorescence quantum yield standards in SWIR spectral region

Here, we point out an urgent need for a critical reevaluation of Φ_{fl} values of NIR/SWIR-emissive nanomaterials. The most common method to determine Φ_{fl} is to compare their fluorescence intensity with a standard material that has a known Φ_{fl}. The first choice of fluoresce standard in the SWIR spectral range is IR-26 in dichloroethane (DCE). IR-26 is a relatively stable dye with absorption and fluorescence in a desirable spectral range, yet it has a narrow absorption spectrum with a very low Φ_{fl}, which led to a poorly characterized Φ_{fl}. The most commonly referred values (Φ_{fl} = 0.5 %) somehow appeared during the development of new NIR/SWIR emitters[3, 4]. These articles refer to the Φ_{fl} value of IR-26 determined by both lifetime measurements and absolute measurement, citing original work from early 1980s that reported the Φ_{fl} value of three IR dyes (no. 5, no. 15, no. 9860). In addition, these articles pointed that Φ_{fl} of IR-26 is similar to that of IR dye no. 5. However, the Φ_{fl} value of IR dye no. 5 reported in the original work is Φ_{fl} = 0.05% in DCE)[5]. In another source[6], Φ_{fl} of IR-26 ($\Phi_{fl} = \tau_F/\tau_{rad}$) was calculated based on previously reported fluorescence lifetime (τ_F) and radiative lifetime (τ_{rad}) derived from the S_0–S_1 absorption cross-section integral and the relative fluorescence quantum distribution.

In 2010, Φ_{fl} of IR-26 was determined using integrating sphere that provided most reliable value Φ_{fl} = 0.048% ± 0.002 for IR-26 in DCE directly[7], suggesting that reported Φ_{fl} values of many SWIR materials were overestimated by a factor of ca.~10 if the old value (Φ_{fl} = 0.5%) has been used. Lately, a large number of researchers started to use IR1061 in DCE as an alternate standard. The authors referred to Φ_{fl} (Φ_{fl} = 1.7%) determined by a direct comparison of the emission intensity of the IR1061 (SWIR) dye in dichloromethane relative to the fluorescence intensity of the rhodamine B (visible) dye in ethanol[8]. Recently, Φ_{fl} of IR-1061 in DCE was redetermined with an integrating sphere instrument, giving the value Φ_{fl} = 0.32%, which renders all Φ_{fl} measured relative to this dye overestimated by a factor of ca.~5.3 times[9].

Fluorescence quantum yields of IR-26 and IR-1061 determined using integrated sphere

Standard	Spectral Range	QY	Source
IR-26 in DCE	excitation: 800–1000nm, emission: 1010–1450 nm	0.048 ± 0.002	[7]
IR-1061 in DCE	Excitation: 750-1150 nm, emission: 1000-1400 nm	0.41 ± 0.02<br>0.32 ± 0.04	[10]<br>[9]

Supporting Table: List of the SWIR emitting organic fluorophores with their spectroscopic properties

Fluorophore<br><br>(core structure)<br><br>#host matrix	Extinction coefficient ($x10^5$ $M^{-1}cm^{-1}$) / peak absorption<br>Mass attenuation coefficient	Emission range / peak wavelength (nm)	Reported Φ_{fl} (%)	Corrected Φ_{fl} (%)	Size (nm)	Ref.
**Small molecules**						
**ICG**<br>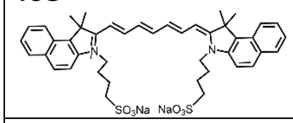	1.56<br>779	740-1100<br>/805	0.71 (SWIR region)<br>2.9 total	0.71	NA	11
**ICG**<br>**Fetal Bovine Serum FBS**	1.62<br>798	740-1100<br>/811	1.43 SWIR only<br>12 all	1.43	NA	11
**IRDye 800CW PEG**<br>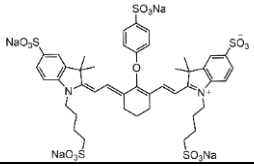	2.4<br>/776	700 – 1,200<br>/ 801	3.3	1.0 – 3.3***	NA	12
**ICG-C11**<br>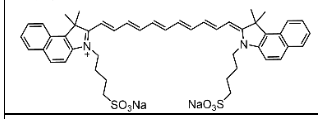	4.3	1030	0.04	0.04	20	13
**FD-1080**<br>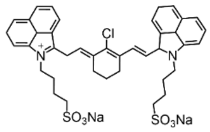<br>**FD-1080-FBS**<br>**(fetal bovine serum)**	2.97<br>/1064	1,000 – 1,400<br>/1,080	0.31<br><br>5.94	0.31<br><br>5.94	NA	14
**Et-1080 (Hydro-1080)**<br>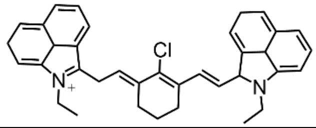	0.937<br>/1039	1000-1400<br>/1044	0.08	0.08	NA	15
**UL-766**<br>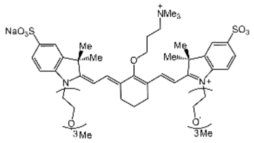<br>**Ureter-Label (UL)**	2.29<br>/766	/789	9.5	9.5	NA	16
**s775z**	2.01	/794	9.0	9.0	NA	17

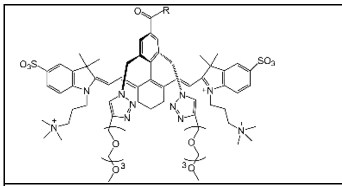	/775					
**LZ-1105**<br>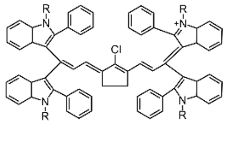	1.01–1.99<br>/1041	1000-1400<br>/1105	1.69	1.69	NA	[18]
**5-Flav7**<br>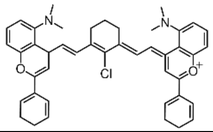	0.79<br>/1004	959-1350<br>/1036	0.14	0.14	NA	[19]
**6-Flav7**<br>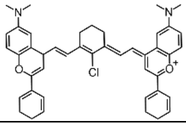	1.6/<br>1048	1000-350<br>/1080	0.12	0.12	NA	[19]
**Flav7**<br>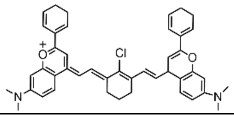	2.41/1027	950-1350<br>/1053	0.61	0.61	NA	[19]
**8-Flav7**<br>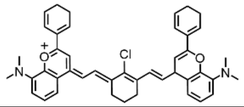	990	800-1150<br>/1015	0.16	0.16	NA	[19]
**BTC1070**<br>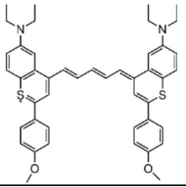	0.45<br>/1015	900-1350<br>/1065	0.016	0.016	NA	[20]
**BTC982**<br>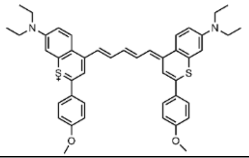	1.375<br>/950	900-1050<br>/988	0.3	0.3	NA	[20]
**BTC980**<br>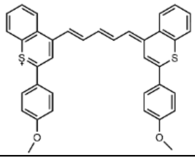	7.96<br>/920	800-1000<br>/950	0.22	0.22	NA	[20]
**MeOFlav7**	1.9<br>/984	/1008	0.52	0.52		[21]

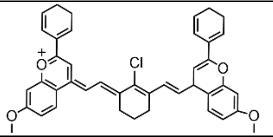						
**JuloFlav7**<br>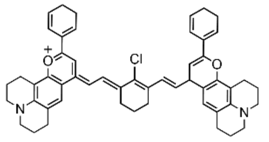<br>**#PEG2000**	2.38<br>/1061	/1088	0.46	0.46	19	[21]
**RosIndz**	0.735<br>/	/1099	0.01	0.01		[22]
**Chrom7**<br>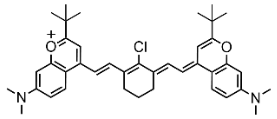<br>**#PEG2000**	2.52<br>/975	/996	1.7	1.7	16	[23]
**Chrom5**<br>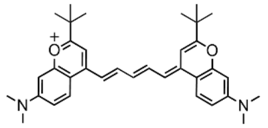<br>**#PEG2000**	3.80<br>/819	/836	2.8	2.8	19	[23]
**Julochrom5**<br>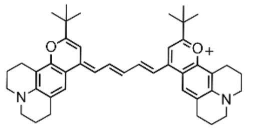<br>**#PEG2000**	3.89<br>/852	/872	18.3	18.3	19	[23]
**Julochrom7**<br>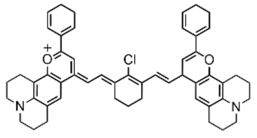<br>***PEG2000**	2.28<br>/1008	/1033	1.58	1.58		[23]
**tolRosIndz**<br>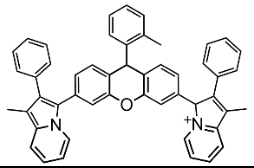	0.735<br>/	/1099	0.26	0.26		[22]
**CX-1**<br>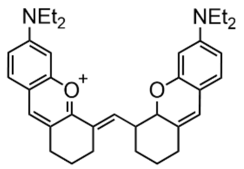<br>***DSPE-mPEG2000**	/880	800-1250<br>/925	0.47	0.47	5.363	[24]
**CX-2**	1.91<br>/972	900-1350<br>/1020	0.23	0.23	8.42	[24]

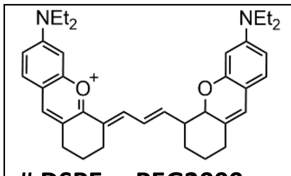<br>**# DSPE-mPEG2000**						
**CX-3**<br>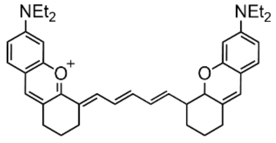<br>**DSPE-mPEG2000**	/1071	1050-1400<br>/1120	0.051	0.051	6.48	[24]
**WH-1**<br>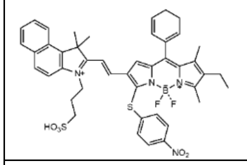	/762	800-1200<br>/925	0.206	0.039	NA	[25]
**WH-2**<br>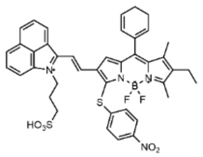	/850	900-1400<br>/1060	0.05	0.009		[25]
**WH-3**<br>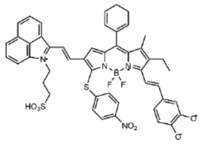	/925	920-1450<br>/1140	0.17	0.032		[25]
**WH-4**<br>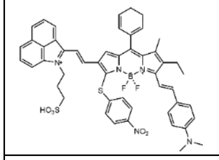	/960	900-1550<br>/1205	0.05	0.009		[25]
**WD-CH3**<br>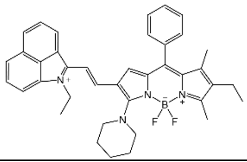	0.257<br>/755	800-1400<br>/1006	0.075	0.022		[26]
**QY-NO**<br>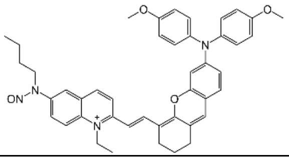	0.421<br>/780	910–1110<br>/935	0.48	0.48		[27]
**IR-FEPC**	/782	900 – 1,400<br>/ 1,053	0.26	0.26		[28]

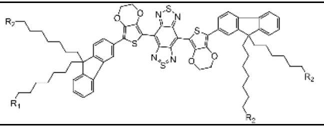						
**IR-FP0P** 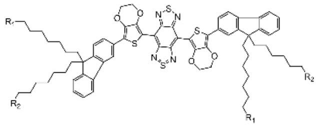	0.12 /732	/1048	0.48	0.48		[29]
**IR-FP8P** 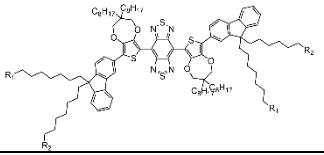	0.13/ 748	900- 1300/1040	0.6	0.6		[29]
**IR-FEP** 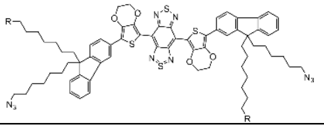	0.057 /780	900-1400 /1047	2.0	0.2		[30]
**IR-FTAP** 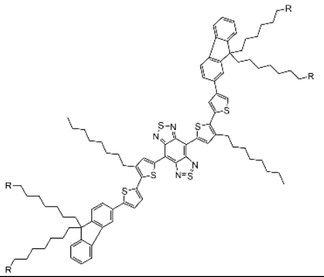	0.05 /733	900 – 1,400 / 1,048	5.3	0.53		[31]
**IR-FTP** 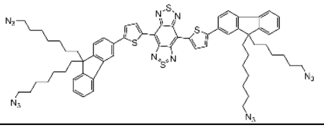	0.797 /828	900-1400 /1047	0.4	0.04		[30]
**IR-BBEP** 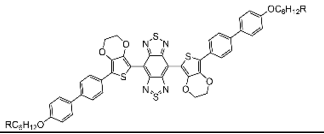	0.41 /741	900-1400 /1047	0.02	0.002		[30]
**CDIR2 [2-hydroxypropyl)-β-cyclodextrin, HPβCD]** 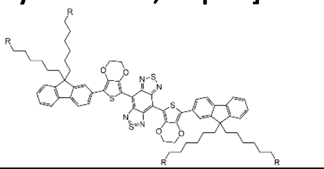	/ 770	820-1300 /1050	2.2	0.22		[32]
**CH4T/PBS**<br><br>**CH4T/FSB**	/738	900 – 1,400 / 1,050	0.098<br><br>4.8	0.098<br><br>0.48		[33]

**CH4T/FBS-HT (heated to 70 °C for 10 min)**<br>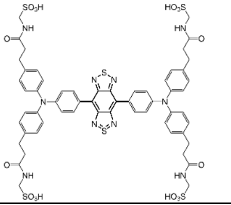			10.8	1.08		
**H3-PEG2k**<br>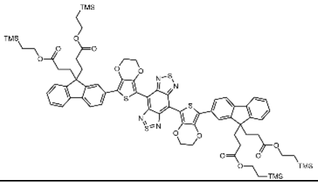	/760	900-1300<br>/1023	1.23	0.123		[34]
**J-Aggregates**						
**MSTPs-FDJ@PAA**<br>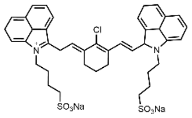<br>**#Mesoporous Silica Coated Titanium Plates (MSTPs)**	0.25<br>/1360	1300-1500<br>/1370	0.0032	0.0032	9.8	[35]
**(PCP-BDP2)**<br>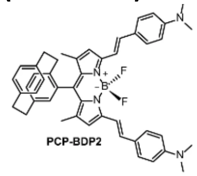<br>**# Pluronic F-127**	0.419<br>/750	900-1300<br>/1010	6.4*	3.2	75	[36]
**FD-1080**<br>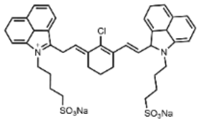<br>**# DMPC**	0.5<br>/1360	/1370	0.0545	0.0545	110	[37]
**IR-140 HMSNs-PEG**<br>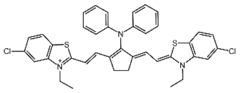<br>*HMSNs	/1038	/1047	0.01	0.01	85	[38]
**THPP**<br>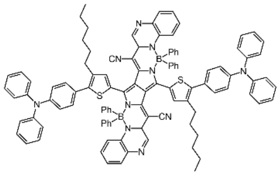<br>**# Pluronic F-127**	2.4<br>/970	/990	0.6	0.6	42.6	[39]
**Small molecule based organic nanoparticles**						
**ZM1068-NPs**	/725; 770	900-1400	0.15	0.15	75.2	[40]

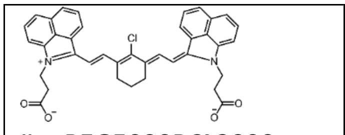 **# mPEG5000PCL3000**		/1050				
**IR-BGP3** 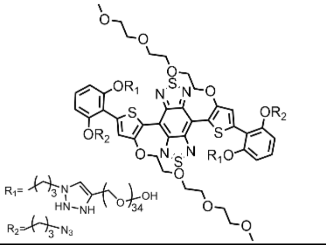	0.072 /714	900 – 1,400 / 1,047	0.9	0.09	2.7	41
**IR-BGP6** 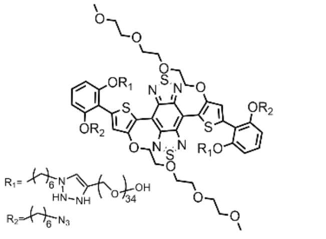	0.081 /736	900 – 1,400 / 1,047	1.5	0.15	3.5	41
**IR-BGP11** 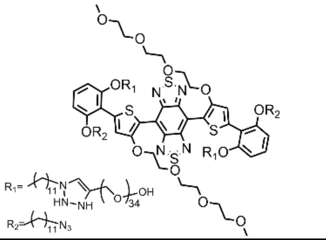	0.108 /763	900 – 1,400 / 1,047	3.0	0.3	40.1	41
**IR-BEMC6P** 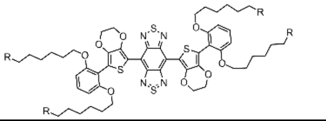	0.0131 /725	900 – 1,400 / 1,028	1.8	0.18	4.0	42
**IR-BTMC6P** 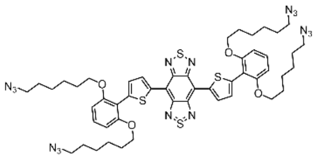	0.0023 /848	900 – 1,400 /1005	0.5	0.05	4.7	42
**IR-BGMC6P** 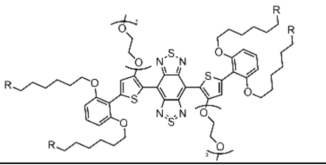	0.242 /737	900 – 1,400 /1036	1.5	0.15	3.5	42

**IR-FGP** 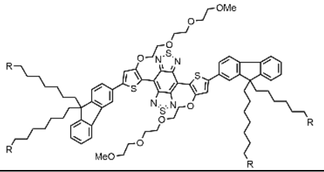	/745	/1050	1.9	0.19	90.1	[43]
**CP-IRT**	/742	900 –1,400 /1,047	1.5	0.15	5	[44]
**T25@F127** 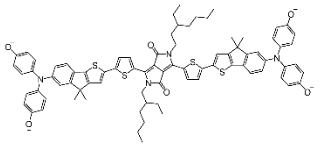 **#Pluronic F-127**	0.715 /700	750-1300 /900	1.84	1.84	30	[45]
**H4–PEG-Glu** 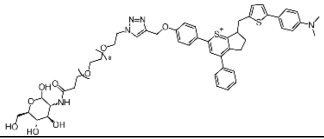	/810	850-1400 /1085	1.3	0.13	96	[46]
**OTPA-BBT dots** 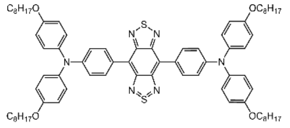 **# Pluronic F-127**	0.5 /770	800-1350 /1020	13.6	1.36	25	[47]
**NIR-II Pdots-GnRH** 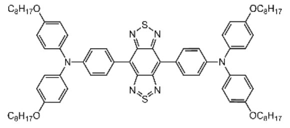 **# PS–PEG–COOH**	/710	800-1350 /1020	5.4	0.54	20	[48]
T-BDP 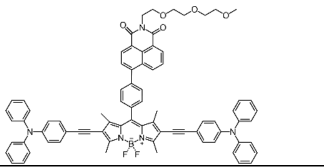	0.473 /631	/742	0.67	0.67	200	[49]
**CH1055-PEG** 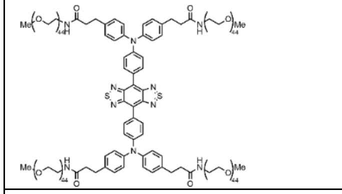	8.5 [48] /750	900-1300 /1055	0.3	0.03	5.8	[50]
**SCH4**	/725	900 – 1,400 / 1,050	0.028	0.028	2	[51]

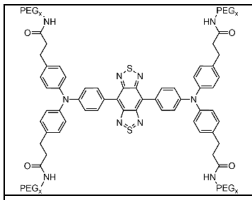						
**SCH1, SCH2, SCH3 NPs** 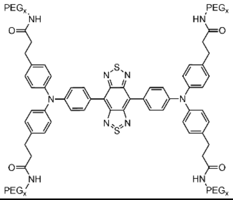	/725 (SCH1:770)	900 – 1,400 / 1,050 (SCH1: 990)	0.14, 0.04, 0.036	0.14, 0.04, 0.036	170, 80, 30	[51]
**TTQ-F-PEG** 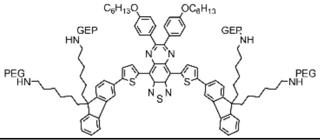	/792	900-1400 /1073	-	-	140	[52]
**IR-E1** 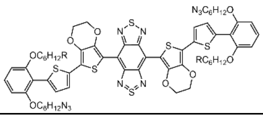	/830	900-1400 /1071	0.7	0.07	3.6	[53]
**OTN-PSt-PEG** 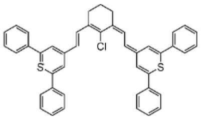 **#Latex**	/1050	1020-1350 /1110	0.65	0.065	40-50	[54]
**IR-1061 NPs** 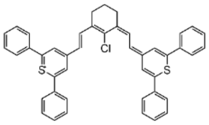 **# DSPE-mPEG5000**	/780	900 – 1,400 / 1,064	1.8	0.18	5.8	[55]
**FIP-99mTc** 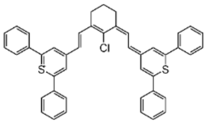 **# Fe3O4 nanoparticles; PLGA;PVA; Na99mTcO4**	750	/1095	-	-	98.4	[56]
**5H5 NPs** 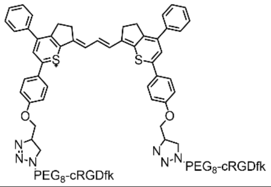	/1069	1000-1400 /1260	2.6	0.26	45	[57]
**CPTIC-4F**	1.45 /876	900-1350 /1110	0.39	0.39	4.5	[58]

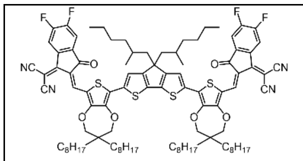 **# DSPE-mPEG2000**						
**COTIC-4F** 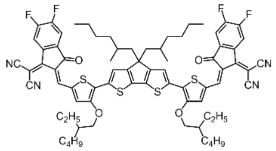 **# DSPE-mPEG2000**	0.87 /934	1000-1350 /1110	0.035	0.035	4.9	[58]
**CBTIC-4F** 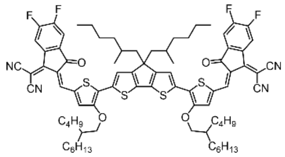 **# DSPE-mPEG2000**	0.88 /949	1000-1350 /1110	0.14	0.14	5.0	[58]
**BDP-T-N-PS-g-PEG** 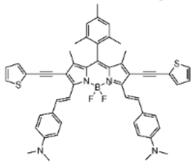 **#PS-g-PEG**	/772	950-1350 /1000	27.6	2.76	5.6	[59]
**CCNU-1060 NPs** 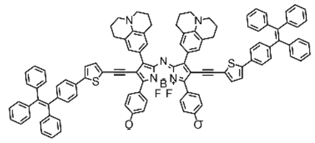 **# DSPE–mPEG5000**	/877	850-1400 /1060	0.03	0.03	100	[60]
**T-IPIC NP** 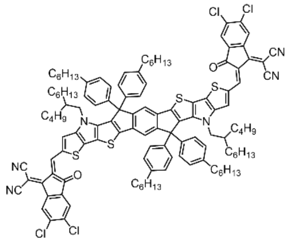 **# TPP-PEG-PPG-PEG-TPP**	2.02 /821	800-1300 /1000	2.2	0.22	80	[61]
**TTDT-TF NPs** 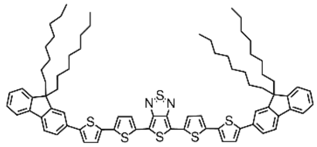 **#DSPE-mPEG5000**	/741	900-1400 /1051	-	-	120	[62]
**TTDT-TSF NPs**	/771	900-1400 /1060	-	-	166	[62]

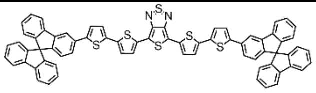<br>**# DSPE-mPEG5000**						
**TPBD-BP**<br>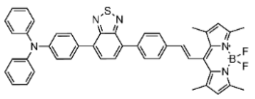<br>**# PEG6000**	/857	/950-1300<br>982; 1080	-	-	435.7	63
**CH1-SV40 dots**<br>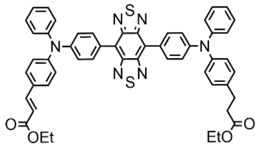<br>**# (Simian Virus 40) SV40 virus-like particles**	/668**	850-1200<br>/955	13.03	1.303	21.5	64
**CH2-SV40 dots**<br>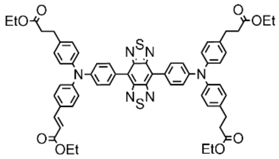<br>**#SV40 VLPs**	/673**	850-1200<br>/942**	4.5	0.45	23.4	64
**Q4NPs SCH1100**<br>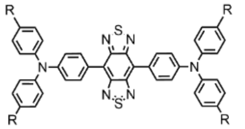<br>R=$CH_2CH_2COOCH_2CH_2TMS$<br>**# DSPE-mPEG5000**	860	900-1400<br>/1100	0.2	0.02	60	65
**TPB-AZO**<br>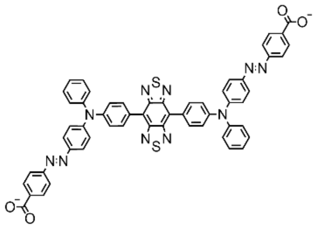<br>**(TA micelles)**<br>**#PS-PEG**	/700	850-1500/<br>900	3.51 (1000-1500)<br>15.62(850-1500)	3.51<br>15.62	43	66
**DTTB@PEG NMs**<br>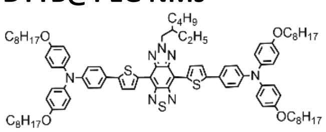<br>**#DSPE-PEG**<br>**(Length 1K, 3K, and 5K)**	/750	900-1350<br>/1050	13.4	2.53	80	67
**2TT-oC6B**	0.08<br>/733	850-1300<br>/1030	11	1.1	160	68

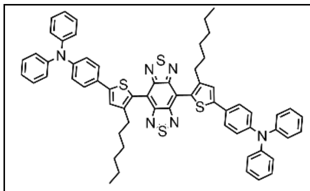<br>**#DSPE-mPEG2000**						
**2TT-Oc26b**<br>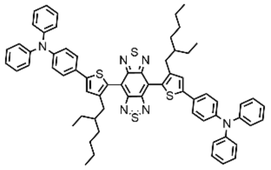<br>**# DSPE-PEG2000**	0.225/ 700	880-1600 /1030	1.15	1.15	60	[69]
**2TT-m,oC6B**<br>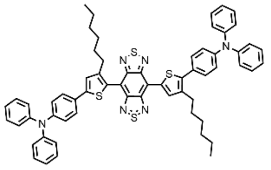<br>**# DSPE-PEG2000**	0.112 /777	800-1400 /1059	3.7	0.37	100	[70]
**TT1-oCB**<br>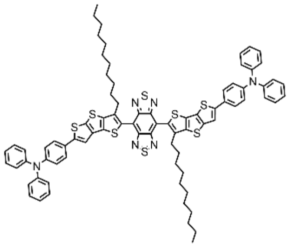<br>**# Pluronic F-127**	0.154 /732	900-1500/1002	8.6	0.86	40	[71]
**TT1-oCB**<br>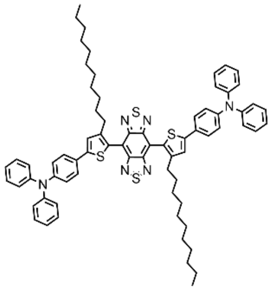<br>**# Pluronic F-127**	0.154 /732	900-1500/1002	8.6	0.86	40	[71]
**TT2-oCB**<br>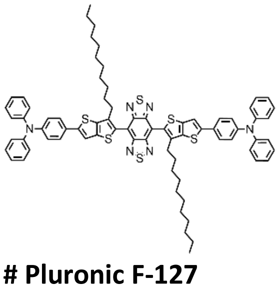<br>**# Pluronic F-127**	0.193 /752	900-1500 /1020	7.8	0.78	40	[71]

**TT3-oCB**<br>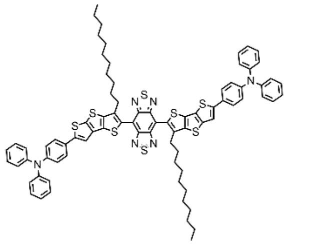<br>*** Pluronic F-127**	0.207<br>/784	900-1500<br>/1062	4.6	0.46	40	[71]
**H1 NPs**<br>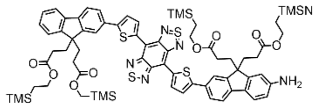<br>**SXH NPs H1 with PEG1000 chains**<br><br>**SDH NPs mono-c(RGDfk) targeting peptide**<br><br>**# DSPE-mPEG5000**	/810	900-1400/<br>1100<br><br>900-1400<br>/1100<br><br>900-1400<br>/1050	2	0.2	70	[72]
**p-FE**<br>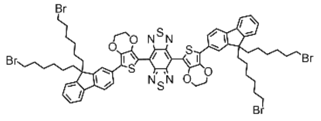<br>**# PS-g-PEG**	/774	900-1350<br>/1010	16.5	1.65	12	[73]
**SYL NPs**<br>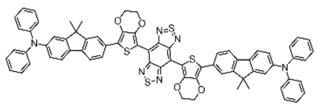<br>**# DSPE-mPEG5000**	/765	850 – 1,300<br>/ 976	-	-	120	
**TQ-BPN NPs**<br>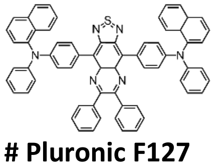<br>**# Pluronic F127**	0.2 [48]<br>/630	700 – 1,200<br>/ 810	13.9	13.9,<br>2.8 (> 900 nm)	33	[74]
**BPN-BBTD NPs**<br>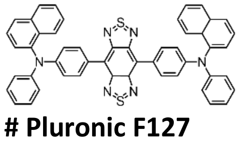<br>**# Pluronic F127**	0.2 [48]<br>/700	800 – 1,200<br>/ 950	1.8	0.18	37	[75]
**HLZ-BTED dots**<br>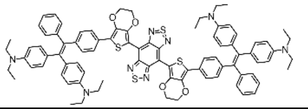	805	900-1400/<br>1034	0.18	0.018	50	[76]

**#DSPE-PEG5000**						
**(L1013 NPs)**<br>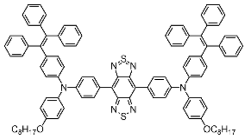<br>**# DSPE-PEG2000**	/761	800-1400<br>/1013	9.9	0.99	38	
XA1 **NPs**<br>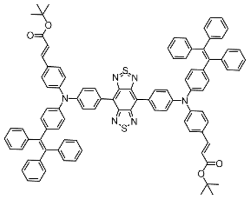<br>**# Pluronic F-127**	780	900-1350<br>/1000	14.8	1.48	38	77
**HQL2 dots**<br>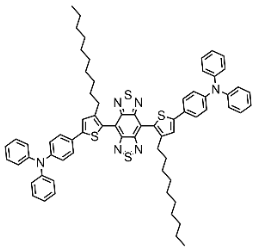<br>**# DSPE-PEG5000**	0.544/<br>710	900-1600<br>/1050	1.19	1.19	125.9	78
HL3 **dots**<br>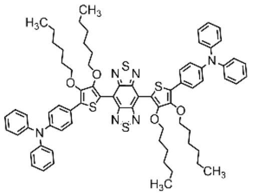<br>*** DPPE-PEG5000**	0.07/<br>750	900-1600<br>/1050	11.7	1.17	90	79
**TB1 dots**<br>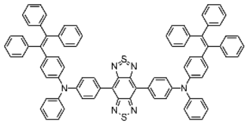<br>**# DSPE-PEG2000**	/740	900-1500<br>/975	6.2	0.62	36-41	80
**L897 NPs**<br>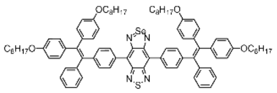<br>**# DSPE-PEG2000**	/711	800 – 1,200<br>/ 897	5.8	0.58	34	81
**FM1210**<br>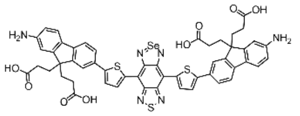<br>**#DSPE-mPEG5000**	/980	900-1600<br>/1210	<0.01	<0.01	20–22	82
**TADAT**	2.6	/1114	0.2	0.02	126,2	83

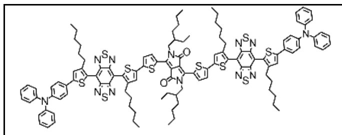<br>**# DSPE-PEG2000**	/754					
**TDADT**<br>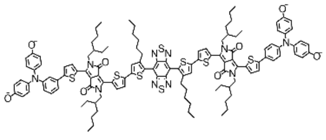<br>**# DSPE-PEG2000**	2.1<br>/-580; 838	/1275	0.1	0.01	131.7	[83]
**DPP-BDT NPs**<br>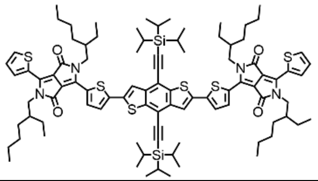	0.335<br>/660	900 – 1,400<br>/ 980	0.52	-	90	[84]
**TQFP-10 NPs**<br>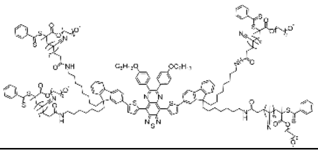	0.15<br>/726	1000–1500<br>1021	1.9	0.66	132	[85]
**TQF@NPs**<br>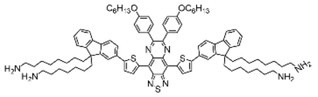<br>**#Pluronic F127**	/736	1000–1500<br>1041	0.9	0.31	165	[85]
**TQF-PEG5K NPs**<br>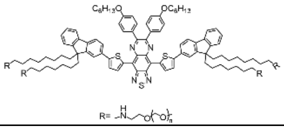	/738	1000–1500<br>/1043	0.5	0.17	192	[85]
**BBT-SF@NPs**<br>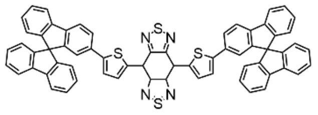<br>**#Pluronic 127**	/818<br>13.03 L g^{-1}<br>cm^{-1}	900-1350<br>/1050	0.04	0.012	90	[86]
**TTQ-SF@NPs**<br>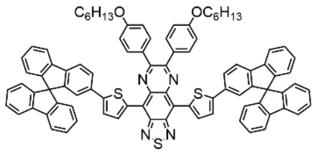<br>**#Pluronic 127**	/735<br>5.96 L g^{-1}<br>cm^{-1}	900-1350<br>/1050	0.84	0.247	110	[86]
**TTDT-SF@NPs**<br>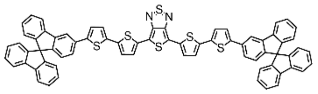<br>**#Pluronic 127**	/704<br>3.91 L g^{-1}<br>cm^{-1}	900-1350<br>/1050	0.15	0.044	104	[86]
**Planar polymers:**						

**PSN (Mn =30kDa)**<br>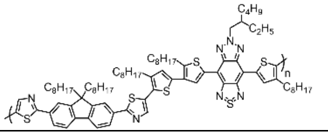	6.4<br>/704	900 – 1,400<br>/ 1,040	0.31	0.31	3.5	[87]
**PSeN (Mn=12.6kDa)**<br>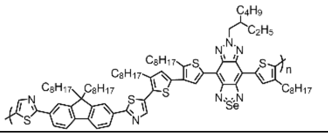	2.9<br>/812	850-1500<br>/934	0.016	0.016	3.0	[87]
**SPNP25 (Mn=43 kDa)**<br>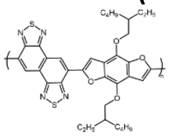<br>**# PEG-b-PPG-b-PEG**	/735	1000- 1,400<br>/ 1,010	0.21	0.021	43	[88]
**PDFT1032 (Mn=52kDa)**<br>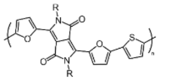<br>**# DSPE-mPEG5000**	/809	850-1400<br>/1032	-	-	68	[89]
**m-PBTQ**<br>**(Mn=25.8kDa)**<br>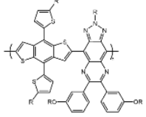<br>**# PS-PEG-COOH**	/923	900-1380<br>/961;1095	1	0.1	21	[90]
**m-PBTQ2F**<br>**(Mn=29.8kDa)**<br>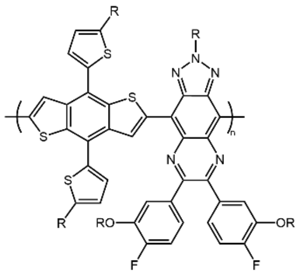<br>**#PS-PEG-COOH**	/931	880-1380<br>/972;1103	2.2	0.22	24	[90]
**m-PBTQ4F**<br>**(Mn=30.2kDa)**<br>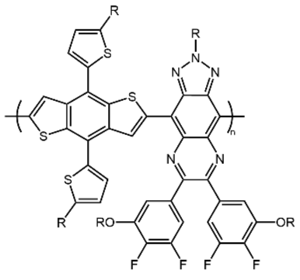<br>**# PS-PEG-COOH**	/946	900-1360<br>/995;1123	3.2	0.32	20	[90]
**p-PBTQ (Mn=25.8kDa)**	/892	800-1350	0.6	0.06	37	[90]

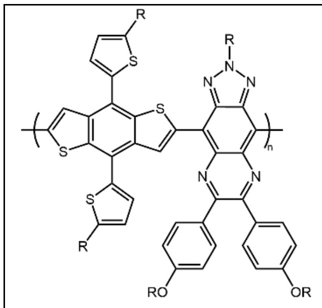 **#PS-PEG-COOH**		/938;1066				
**p-PBTQ2F (Mn=26.1kDa)** 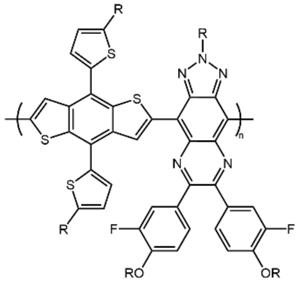 **#PS-PEG-COOH**	/925	900-1350 /956;1089	0.9	0.09	28	[90]
**p-PBTQ4F (Mn=26.7kDa)** 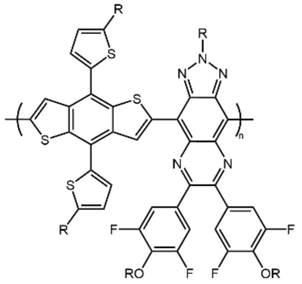 **#PS-PEG-COOH**	/941	900-1350 /986;1117	1.5	0.15	32	[90]
**CN-PPV/PBTQ4F** 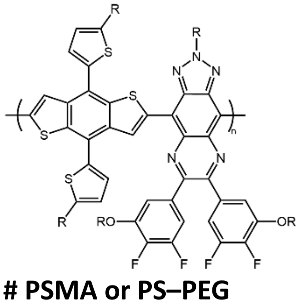 **# PSMA or PS–PEG**	/944	900-1320 /990;1100	1.9	0.19	15	[91]
**pDA-PEG (Mn=16.19kDa)** 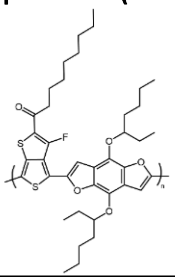	/654	900-1350 /1047	1.7	0.17	2.9 (17.8 DLS)	[92]

**#*DSPE-PEG5000**						
**TT-T CPs (Mn=29 kDa)** 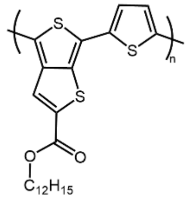 **#Pluronic F-127**	/812	900-1380 /1108;1250	-	-	51-70	[93]
**TT-2T CPs** 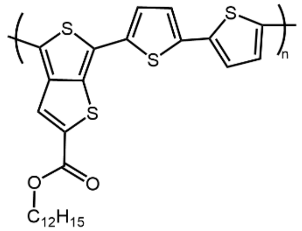 **#Pluronic F-127**	/667	900-1380 /1077;1250	-	-	51-70	[93]
**TT-3T CPs (Mn=58kDa)** 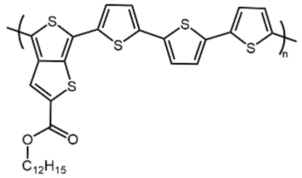 **#Pluronic F-127**	/640	900-1380 /1090;1250	1.75	0.33	51-70	[93]
**SPN (Mn=31.36kDa)** 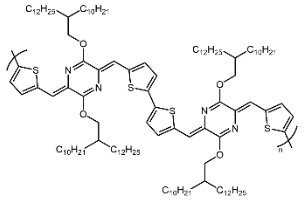 **# DSPE-mPEG2000**	/521	850-1350 /930	0.2	0.02	44.5	[94]
**Lip(DPQ+2DG) NPs** 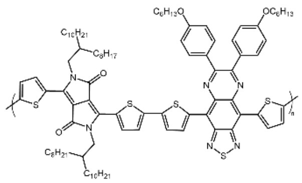 **# DSPE-PEG5000-FA**	/1015	1200-1400 /1300	-	-	100	[95]
P-TT	3.58 /686	800-1400 /1071	0.5	0.05	20-40	[96]

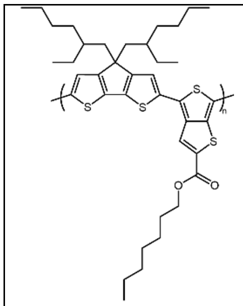<br>**# DSPE-mPEG2000**						
P-DPP<br>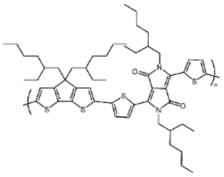<br>**# DSPE-mPEG2000**	8.37<br>/763	800-1400<br>/1066	1.5	0.15	20-40	[96]
**PBT (Mn=71.2 kDa)**<br>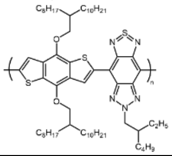	/998<br>35.2 L g^{-1} cm^{-1}	1000-1600<br>/1156	0.1	0.01	50	[97]
**P1-Pdots (Mn=25.8kDa)**<br>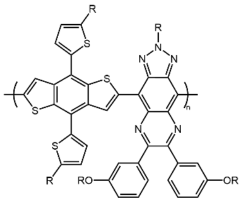<br>**# PS–PEG–COOH**	/923	900-1400<br>/1095	0.98	0.098	28	[98]
**P2-Pdots (Mn=24.2kDa)**<br>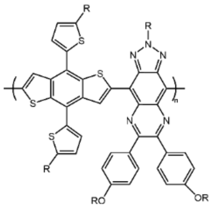<br>**# PS–PEG–COOH**	/870	900-1400<br>/1063	-	-	37	[98]
**P3-Pdots (Mn=21.9kDa)**<br>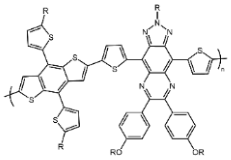<br>**# PS–PEG–COOH**	/703	900-1400<br>/1058	-	-	24	[98]
**P1 (Double-acceptor type) (Mn=40kDa)**	/705<br>23.7 L g^{-1} cm^{-1}	900-1500<br>/1257	0.1	0.019	96	[99]

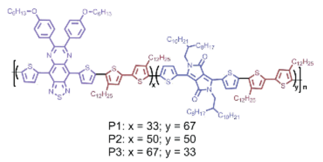						
**P2 (Double-acceptor type) (Mn=31kDa)**<br>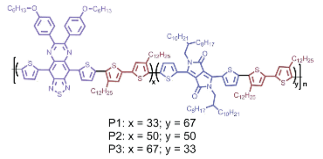	/609<br>13.6 L g^{-1} cm^{-1}	900-1500 /1267	0.08	0.015	92	[99]
**P3 (Double-acceptor type) (Mn=34kDa)**<br>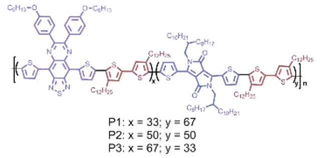	/712<br>15.0 L g^{-1} cm^{-1}	900-1500 /1272	0.05	0.009	106	[99]
**L1057 NPs PTQ polymer (Mn=8.044kDa)**<br>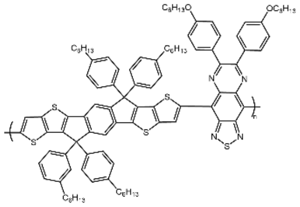<br>**# DSPE-PEG2000**	/937	850-1400 /1057	1.25	0.125	51	[100]
**Twisted polymers:**						
**BBT (Mn=5.5kDa)**<br>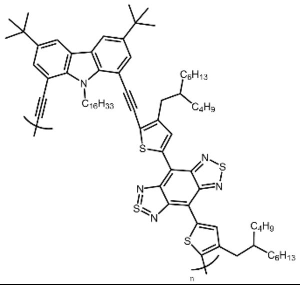	/835	900 – 1,400 / 1,035	0.29	0.29	3.6	[87]
**P3a Pdots (Mn=3.2 kDa)**<br>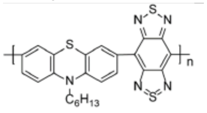<br>P3a<br>**# PS-PEG1000**	/736	900-1400 /1090	0.1	0.01	24	[101]
**P3b Pdots (Mn=4.9 kDa)**	/759	900-1400 /1088	0.6	0.06	14	[101]

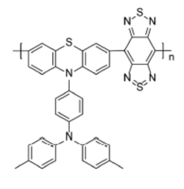<br>P3b<br>**# PS-PEG1000**						
**P3c Pdots (Mn=2.8 kDa)**<br>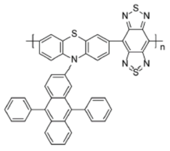<br>P3c<br>**# PS-PEG1000**	746	900-1500<br>/1083	1.7	0.17	16	[101]
**pNIR-4 (Mn=4.8kDa)**<br>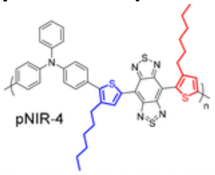<br>**# DSPE-mPEG2000**	/750	850-1400<br>/1040	2.24	0.24	103	[102]
**pNIR-3 (Mn=9.2kDa)**<br>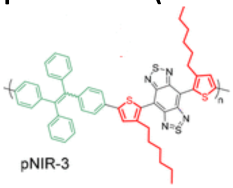<br>**# DSPE-mPEG2000**	/688	850-1300<br>/925	1.96	0.196		[102]
**pNIR-2**<br>**(Mn=5.9kDa)**<br>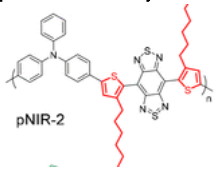<br>**# DSPE-mPEG2000**	/700	800-1350<br>/1030	3.2	0.32		[102]
**NK@AIEdots**<br>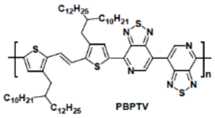<br>**# DSPE-PEG200 + natural killer cell membrane wrapping**	/700	900-1200<br>/960	7.9	0.79	78	[103]
**Pttc-TaQ-NIR855**	1880<br>/816	800-1000<br>/855	12	12	62	[104]

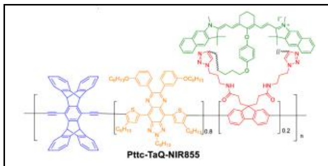 **# CM–PEG–DSPE**						
**Pttc-TaQ-NIR917** 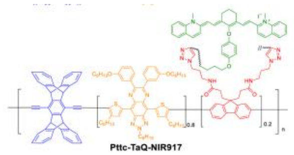 **# CM–PEG–DSPE**	/865	850-1200 /917	2	2	65	104
**Pttc-SeBTa-NIR1125** 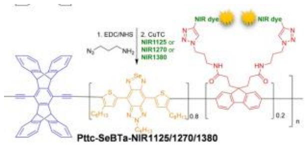 **# CM–PEG–DSPE**	1270 /1082	1000-1280/1115	0.18	0.18	35	105
**Pttc-SeBTa-NIR1270** 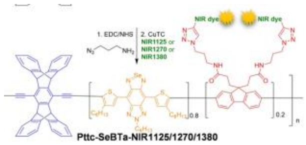 **# CM–PEG–DSPE**	1730 /1200	1000-1350 /1225	0.09	0.09	39	105
**Pttc-SeBTa-NIR1380** 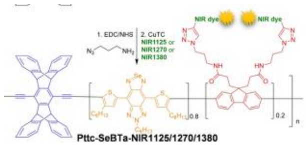 **# CM–PEG–DSPE**	1080 /1290	1000-1500 /1300	0.05	0.05	73	105

Second column of the table: light absorption efficiency is shown in either molar extinction coefficient (10^5 M^{-1} cm^{-1}, black) or mass attenuation coefficient (L g^{-1} cm^{-1}, red).

*reference dye IR 26, Φf = 0.1%.

**based on spectra in SI.

*** The reported values were calculated based on the QYs of the ICG dye reported in the reference [https://doi.org/10.1088/0031-9155/23/1/017] and [https://doi.org/10.1073/pnas.1718917115]. Since the reported QY of ICG varies, these QY values remain a matter of debate.

Supporting References

1. Wang, L.; Shen, Y.; Yang, M.; Zhang, X.; Xu, W.; Zhu, Q.; Wu, J.; Tian, Y.; Zhou, H., Novel highly emissive H-aggregates with aggregate fluorescence change in a phenylbenzoxazole-based system. *Chemical Communications* **2014,** *50* (63), 8723-8726.

2. Piwoński, H.; Nozue, S.; Fujita, H.; Michinobu, T.; Habuchi, S., Organic J-Aggregate Nanodots with Enhanced Light Absorption and Near-Unity Fluorescence Quantum Yield. *Nano Letters* **2021,** *21* (7), 2840-2847.
3. Murphy, J. E.; Beard, M. C.; Norman, A. G.; Ahrenkiel, S. P.; Johnson, J. C.; Yu, P. R.; Micic, O. I.; Ellingson, R. J.; Nozik, A. J., PbTe colloidal nanocrystals: Synthesis, characterization, and multiple exciton generation. *Journal of the American Chemical Society* **2006,** *128* (10), 3241-3247.
4. Wehrenberg, B. L.; Wang, C. J.; Guyot-Sionnest, P., Interband and intraband optical studies of PbSe colloidal quantum dots. *J. Phys. Chem. B* **2002,** *106* (41), 10634-10640.
5. Seilmeier, A.; Kopainsky, B.; Kaiser, W., Infrared fluorescence and laser action of fast mode-locking dyes. *Applied physics* **1980,** *22* (4), 355-359.
6. Penzkofer, A.; Lammel, O.; Tsuboi, T., Emission spectroscopic characterisation of F2− colour centres in a LiF crystal. *Optics Communications* **2002,** *214* (1), 305-313.
7. Semonin, O. E.; Johnson, J. C.; Luther, J. M.; Midgett, A. G.; Nozik, A. J.; Beard, M. C., Absolute Photoluminescence Quantum Yields of IR-26 Dye, PbS, and PbSe Quantum Dots. *The Journal of Physical Chemistry Letters* **2010,** *1* (16), 2445-2450.
8. Casalboni, M.; De Matteis, F.; Prosposito, P.; Quatela, A.; Sarcinelli, F., Fluorescence efficiency of four infrared polymethine dyes. *Chemical Physics Letters* **2003,** *373* (3), 372-378.
9. Cosco, E. D.; Caram, J. R.; Bruns, O. T.; Franke, D.; Day, R. A.; Farr, E. P.; Bawendi, M. G.; Sletten, E. M., Flavylium Polymethine Fluorophores for Near- and Shortwave Infrared Imaging. *Angewandte Chemie International Edition* **2017,** *56* (42), 13126-13129.
10. Hoshi, R.; Suzuki, K.; Hasebe, N.; Yoshihara, T.; Tobita, S., Absolute Quantum Yield Measurements of Near-Infrared Emission with Correction for Solvent Absorption. *Analytical Chemistry* **2020,** *92* (1), 607-611.
11. Cosco, E. D.; Lim, I.; Sletten, E. M., Photophysical Properties of Indocyanine Green in the Shortwave Infrared Region. *ChemPhotoChem* **2021,** *5* (8), 727-734.
12. Carr, J. A.; Franke, D.; Caram, J. R.; Perkinson, C. F.; Saif, M.; Askoxylakis, V.; Datta, M.; Fukumura, D.; Jain, R. K.; Bawendi, M. G.; Bruns, O. T., Shortwave infrared fluorescence imaging with the clinically approved near-infrared dye indocyanine green. *Proceedings of the National Academy of Sciences* **2018,** *115* (17), 4465-4470.
13. Swamy, M. M. M.; Murai, Y.; Monde, K.; Tsuboi, S.; Jin, T., Shortwave-Infrared Fluorescent Molecular Imaging Probes Based on π-Conjugation Extended Indocyanine Green. *Bioconjugate Chemistry* **2021,** *32* (8), 1541-1547.
14. Li, B.; Lu, L.; Zhao, M.; Lei, Z.; Zhang, F., An Efficient 1064 nm NIR-II Excitation Fluorescent Molecular Dye for Deep-Tissue High-Resolution Dynamic Bioimaging. *Angewandte Chemie International Edition* **2018,** *57* (25), 7483-7487.
15. Feng, W.; Zhang, Y.; Li, Z.; Zhai, S.; Lv, W.; Liu, Z., Lighting Up NIR-II Fluorescence in Vivo: An Activable Probe for Noninvasive Hydroxyl Radical Imaging. *Analytical Chemistry* **2019,** *91* (24), 15757-15762.
16. Cha, J.; Nani, R. R.; Luciano, M. P.; Kline, G.; Broch, A.; Kim, K.; Namgoong, J.-M.; Kulkarni, R. A.; Meier, J. L.; Kim, P.; Schnermann, M. J., A chemically stable fluorescent marker of the ureter. *Bioorganic & Medicinal Chemistry Letters* **2018,** *28* (16), 2741-2745.
17. Li, D.-H.; Schreiber, C. L.; Smith, B. D., Sterically Shielded Heptamethine Cyanine Dyes for Bioconjugation and High Performance Near-Infrared Fluorescence Imaging. *Angewandte Chemie International Edition* **2020,** *59* (29), 12154-12161.
18. Li, B.; Zhao, M.; Feng, L.; Dou, C.; Ding, S.; Zhou, G.; Lu, L.; Zhang, H.; Chen, F.; Li, X.; Li, G.; Zhao, S.; Jiang, C.; Wang, Y.; Zhao, D.; Cheng, Y.; Zhang, F., Organic NIR-II molecule with long blood half-life for in vivo dynamic vascular imaging. *Nature Communications* **2020,** *11* (1), 3102.

19. Pengshung, M.; Li, J.; Mukadum, F.; Lopez, S. A.; Sletten, E. M., Photophysical Tuning of Shortwave Infrared Flavylium Heptamethine Dyes via Substituent Placement. *Organic Letters* **2020,** *22* (15), 6150-6154.
20. Wang, S.; Fan, Y.; Li, D.; Sun, C.; Lei, Z.; Lu, L.; Wang, T.; Zhang, F., Anti-quenching NIR-II molecular fluorophores for in vivo high-contrast imaging and pH sensing. *Nature Communications* **2019,** *10* (1), 1058.
21. Tian, C.; Burgess, K., Flavylium- and Silylrhodapolymethines In Excitation Multiplexing. *ChemPhotoChem* **2021,** *5* (8), 702-704.
22. Chatterjee, S.; Meador, W. E.; Smith, C.; Chandrasiri, I.; Zia, M. F.; Nguyen, J.; Dorris, A.; Flynt, A.; Watkins, D. L.; Hammer, N. I.; Delcamp, J. H., SWIR emissive RosIndolizine dyes with nanoencapsulation in water soluble dendrimers. *RSC Advances* **2021,** *11* (45), 27832-27836.
23. Cosco, E. D.; Arús, B. A.; Spearman, A. L.; Atallah, T. L.; Lim, I.; Leland, O. S.; Caram, J. R.; Bischof, T. S.; Bruns, O. T.; Sletten, E. M., Bright Chromenylium Polymethine Dyes Enable Fast, Four-Color In Vivo Imaging with Shortwave Infrared Detection. *Journal of the American Chemical Society* **2021,** *143* (18), 6836-6846.
24. Lei, Z.; Sun, C.; Pei, P.; Wang, S.; Li, D.; Zhang, X.; Zhang, F., Stable, Wavelength-Tunable Fluorescent Dyes in the NIR-II Region for In Vivo High-Contrast Bioimaging and Multiplexed Biosensing. *Angewandte Chemie International Edition* **2019,** *58* (24), 8166-8171.
25. Dou, K.; Feng, W.; Fan, C.; Cao, Y.; Xiang, Y.; Liu, Z., Flexible Designing Strategy to Construct Activatable NIR-II Fluorescent Probes with Emission Maxima beyond 1200 nm. *Analytical Chemistry* **2021,** *93* (8), 4006-4014.
26. Dou, K.; Huang, W.; Xiang, Y.; Li, S.; Liu, Z., Design of Activatable NIR-II Molecular Probe for In Vivo Elucidation of Disease-Related Viscosity Variations. *Analytical Chemistry* **2020,** *92* (6), 4177-4181.
27. Sun, L.; Ouyang, J.; Ma, Y.; Zeng, Z.; Zeng, C.; Zeng, F.; Wu, S., An Activatable Probe with Aggregation-Induced Emission for Detecting and Imaging Herbal Medicine Induced Liver Injury with Optoacoustic Imaging and NIR-II Fluorescence Imaging. *Advanced Healthcare Materials n/a* (n/a), 2100867.
28. Zhu, S.; Herraiz, S.; Yue, J.; Zhang, M.; Wan, H.; Yang, Q.; Ma, Z.; Wang, Y.; He, J.; Antaris, A. L.; Zhong, Y.; Diao, S.; Feng, Y.; Zhou, Y.; Yu, K.; Hong, G.; Liang, Y.; Hsueh, A. J.; Dai, H., 3D NIR-II Molecular Imaging Distinguishes Targeted Organs with High-Performance NIR-II Bioconjugates. *Advanced Materials* **2018,** *30* (13), 1705799.
29. Ma, H.; Liu, C.; Hu, Z.; Yu, P.; Zhu, X.; Ma, R.; Sun, Z.; Zhang, C.-H.; Sun, H.; Zhu, S.; Liang, Y., Propylenedioxy Thiophene Donor to Achieve NIR-II Molecular Fluorophores with Enhanced Brightness. *Chemistry of Materials* **2020,** *32* (5), 2061-2069.
30. Yang, Q.; Ma, Z.; Wang, H.; Zhou, B.; Zhu, S.; Zhong, Y.; Wang, J.; Wan, H.; Antaris, A.; Ma, R.; Zhang, X.; Yang, J.; Zhang, X.; Sun, H.; Liu, W.; Liang, Y.; Dai, H., Rational Design of Molecular Fluorophores for Biological Imaging in the NIR-II Window. *Advanced Materials* **2017,** *29* (12), 1605497.
31. Yang, Q.; Hu, Z.; Zhu, S.; Ma, R.; Ma, H.; Ma, Z.; Wan, H.; Zhu, T.; Jiang, Z.; Liu, W.; Jiao, L.; Sun, H.; Liang, Y.; Dai, H., Donor Engineering for NIR-II Molecular Fluorophores with Enhanced Fluorescent Performance. *Journal of the American Chemical Society* **2018,** *140* (5), 1715-1724.
32. Huang, J.; Xie, C.; Zhang, X.; Jiang, Y.; Li, J.; Fan, Q.; Pu, K., Renal-clearable Molecular Semiconductor for Second Near-Infrared Fluorescence Imaging of Kidney Dysfunction. *Angewandte Chemie International Edition* **2019,** *58* (42), 15120-15127.
33. Antaris, A. L.; Chen, H.; Diao, S.; Ma, Z.; Zhang, Z.; Zhu, S.; Wang, J.; Lozano, A. X.; Fan, Q.; Chew, L.; Zhu, M.; Cheng, K.; Hong, X.; Dai, H.; Cheng, Z., A high quantum yield molecule-protein complex fluorophore for near-infrared II imaging. *Nature Communications* **2017,** *8* (1), 15269.

34. Zeng, X.; Xie, L.; Chen, D.; Li, S.; Nong, J.; Wang, B.; Tang, L.; Li, Q.; Li, Y.; Deng, Z.; Hong, X.; Wu, M.; Xiao, Y., A bright NIR-II fluorescent probe for breast carcinoma imaging and image-guided surgery. *Chemical Communications* **2019,** *55* (95), 14287-14290.
35. Sun, C.; Sun, X.; Pei, P.; He, H.; Ming, J.; Liu, X.; Liu, M.; Zhang, Y.; Xia, Y.; Zhao, D.; Li, X.; Xie, Y.; Zhang, F., NIR-II J-Aggregates Labelled Mesoporous Implant for Imaging-Guided Osteosynthesis with Minimal Invasion. *Advanced Functional Materials* **2021,** *31* (23), 2100656.
36. Li, K.; Duan, X.; Jiang, Z.; Ding, D.; Chen, Y.; Zhang, G.-Q.; Liu, Z., J-aggregates of meso-[2.2]paracyclophanyl-BODIPY dye for NIR-II imaging. *Nature Communications* **2021,** *12* (1), 2376.
37. Sun, C.; Li, B.; Zhao, M.; Wang, S.; Lei, Z.; Lu, L.; Zhang, H.; Feng, L.; Dou, C.; Yin, D.; Xu, H.; Cheng, Y.; Zhang, F., J-Aggregates of Cyanine Dye for NIR-II in Vivo Dynamic Vascular Imaging beyond 1500 nm. *Journal of the American Chemical Society* **2019,** *141* (49), 19221-19225.
38. Chen, W.; Cheng, C.-A.; Cosco, E. D.; Ramakrishnan, S.; Lingg, J. G. P.; Bruns, O. T.; Zink, J. I.; Sletten, E. M., Shortwave Infrared Imaging with J-Aggregates Stabilized in Hollow Mesoporous Silica Nanoparticles. *Journal of the American Chemical Society* **2019,** *141* (32), 12475-12480.
39. Zhang, Q.; Yu, P.; Fan, Y.; Sun, C.; He, H.; Liu, X.; Lu, L.; Zhao, M.; Zhang, H.; Zhang, F., Bright and Stable NIR-II J-Aggregated AIE Dibodipy-Based Fluorescent Probe for Dynamic In Vivo Bioimaging. *Angewandte Chemie International Edition* **2021,** *60* (8), 3967-3973.
40. Zhang, Y.; Zhao, M.; Fang, J.; Ye, S.; Wang, A.; Zhao, Y.; Cui, C.; He, L.; Shi, H., Smart On-Site Immobilizable Near-Infrared II Fluorescent Nanoprobes for Ultra-Long-Term Imaging-Guided Tumor Surgery and Photothermal Therapy. *ACS Applied Materials & Interfaces* **2021,** *13* (11), 12857-12865.
41. Wan, H.; Ma, H.; Zhu, S.; Wang, F.; Tian, Y.; Ma, R.; Yang, Q.; Hu, Z.; Zhu, T.; Wang, W.; Ma, Z.; Zhang, M.; Zhong, Y.; Sun, H.; Liang, Y.; Dai, H., Developing a Bright NIR-II Fluorophore with Fast Renal Excretion and Its Application in Molecular Imaging of Immune Checkpoint PD-L1. *Advanced Functional Materials* **2018,** *28* (50), 1804956.
42. Tian, R.; Ma, H.; Yang, Q.; Wan, H.; Zhu, S.; Chandra, S.; Sun, H.; Kiesewetter, D. O.; Niu, G.; Liang, Y.; Chen, X., Rational design of a super-contrast NIR-II fluorophore affords high-performance NIR-II molecular imaging guided microsurgery. *Chemical Science* **2019,** *10* (1), 326-332.
43. Zhu, S.; Yang, Q.; Antaris, A. L.; Yue, J.; Ma, Z.; Wang, H.; Huang, W.; Wan, H.; Wang, J.; Diao, S.; Zhang, B.; Li, X.; Zhong, Y.; Yu, K.; Hong, G.; Luo, J.; Liang, Y.; Dai, H., Molecular imaging of biological systems with a clickable dye in the broad 800- to 1,700-nm near-infrared window. *Proceedings of the National Academy of Sciences* **2017,** *114* (5), 962-967.
44. Wang, W.; Ma, Z.; Zhu, S.; Wan, H.; Yue, J.; Ma, H.; Ma, R.; Yang, Q.; Wang, Z.; Li, Q.; Qian, Y.; Yue, C.; Wang, Y.; Fan, L.; Zhong, Y.; Zhou, Y.; Gao, H.; Ruan, J.; Hu, Z.; Liang, Y.; Dai, H., Molecular Cancer Imaging in the Second Near-Infrared Window Using a Renal-Excreted NIR-II Fluorophore-Peptide Probe. *Advanced Materials* **2018,** *30* (22), 1800106.
45. Yang, Z.; Fan, X.; Li, H.; Li, X.; Li, S.; Zhang, Z.; Lin, H.; Qian, J.; Hua, J., A Small-Molecule Diketopyrrolopyrrole-Based Dye for in vivo NIR-IIa Fluorescence Bioimaging. *Chemistry – A European Journal n/a* (n/a).
46. Zheng, Y.; Li, Q.; Wu, J.; Luo, Z.; Zhou, W.; Li, A.; Chen, Y.; Rouzi, T.; Tian, T.; Zhou, H.; Zeng, X.; Li, Y.; Cheng, X.; Wei, Y.; Deng, Z.; Zhou, F.; Hong, X., All-in-one mitochondria-targeted NIR-II fluorophores for cancer therapy and imaging. *Chemical Science* **2021,** *12* (5), 1843-1850.
47. Feng, Z.; Bai, S.; Qi, J.; Sun, C.; Zhang, Y.; Yu, X.; Ni, H.; Wu, D.; Fan, X.; Xue, D.; Liu, S.; Chen, M.; Gong, J.; Wei, P.; He, M.; Lam, J. W. Y.; Li, X.; Tang, B. Z.; Gao, L.; Qian, J., Biologically Excretable Aggregation-Induced Emission Dots for Visualizing Through the Marmosets Intravitally: Horizons in Future Clinical Nanomedicine. *Advanced Materials* **2021,** *33* (17), 2008123.
48. Zhou, X.; Liu, Q.; Yuan, W.; Li, Z.; Xu, Y.; Feng, W.; Xu, C.; Li, F., Ultrabright NIR-II Emissive Polymer Dots for Metastatic Ovarian Cancer Detection. *Advanced Science* **2021,** *8* (4), 2000441.

49. Yang, M.; Deng, J.; Su, H.; Gu, S.; Zhang, J.; Zhong, A.; Wu, F., Small organic molecule-based nanoparticles with red/near-infrared aggregation-induced emission for bioimaging and PDT/PTT synergistic therapy. *Materials Chemistry Frontiers* **2021,** *5* (1), 406-417.
50. Antaris, A. L.; Chen, H.; Cheng, K.; Sun, Y.; Hong, G.; Qu, C.; Diao, S.; Deng, Z.; Hu, X.; Zhang, B.; Zhang, X.; Yaghi, O. K.; Alamparambil, Z. R.; Hong, X.; Cheng, Z.; Dai, H., A small-molecule dye for NIR-II imaging. *Nature Materials* **2016,** *15* (2), 235-242.
51. Ding, F.; Li, C.; Xu, Y.; Li, J.; Li, H.; Yang, G.; Sun, Y., PEGylation Regulates Self-Assembled Small-Molecule Dye–Based Probes from Single Molecule to Nanoparticle Size for Multifunctional NIR-II Bioimaging. *Advanced Healthcare Materials* **2018,** *7* (23), 1800973.
52. He, K.; Chen, S.; Chen, Y.; Li, J.; Sun, P.; Lu, X.; Fan, Q.; Huang, W., Water-Soluble Donor–Acceptor–Donor-Based Fluorophore for High-Resolution NIR-II Fluorescence Imaging Applications. *ACS Applied Polymer Materials* **2021,** *3* (6), 3238-3246.
53. Zhang, X.-D.; Wang, H.; Antaris, A. L.; Li, L.; Diao, S.; Ma, R.; Nguyen, A.; Hong, G.; Ma, Z.; Wang, J.; Zhu, S.; Castellano, J. M.; Wyss-Coray, T.; Liang, Y.; Luo, J.; Dai, H., Traumatic Brain Injury Imaging in the Second Near-Infrared Window with a Molecular Fluorophore. *Advanced Materials* **2016,** *28* (32), 6872-6879.
54. Ueya, Y.; Umezawa, M.; Takamoto, E.; Yoshida, M.; Kobayashi, H.; Kamimura, M.; Soga, K., Designing highly emissive over-1000 nm near-infrared fluorescent dye-loaded polystyrene-based nanoparticles for in vivo deep imaging. *RSC Advances* **2021,** *11* (31), 18930-18937.
55. Tao, Z.; Hong, G.; Shinji, C.; Chen, C.; Diao, S.; Antaris, A. L.; Zhang, B.; Zou, Y.; Dai, H., Biological Imaging Using Nanoparticles of Small Organic Molecules with Fluorescence Emission at Wavelengths Longer than 1000 nm. *Angewandte Chemie International Edition* **2013,** *52* (49), 13002-13006.
56. Cai, W.; Fan, G.; Zhou, H.; Chen, L.; Ge, J.; Huang, B.; Zhou, D.; Zeng, J.; Miao, Q.; Hu, C., Self-Assembled Hybrid Nanocomposites for Multimodal Imaging-Guided Photothermal Therapy of Lymph Node Metastasis. *ACS Applied Materials & Interfaces* **2020,** *12* (44), 49407-49415.
57. Ding, B.; Xiao, Y.; Zhou, H.; Zhang, X.; Qu, C.; Xu, F.; Deng, Z.; Cheng, Z.; Hong, X., Polymethine Thiopyrylium Fluorophores with Absorption beyond 1000 nm for Biological Imaging in the Second Near-Infrared Subwindow. *Journal of Medicinal Chemistry* **2019,** *62* (4), 2049-2059.
58. Zhu, X.; Liu, C.; Hu, Z.; Liu, H.; Wang, J.; Wang, Y.; Wang, X.; Ma, R.; Zhang, X.; Sun, H.; Liang, Y., High brightness NIR-II nanofluorophores based on fused-ring acceptor molecules. *Nano Research* **2020,** *13* (9), 2570-2575.
59. Liu, Q.; Tian, J.; Tian, Y.; Sun, Q.; Sun, D.; Liu, D.; Wang, F.; Xu, H.; Ying, G.; Wang, J.; Yetisen, A. K.; Jiang, N., Thiophene donor for NIR-II fluorescence imaging-guided photothermal/photodynamic/chemo combination therapy. *Acta Biomaterialia* **2021,** *127*, 287-297.
60. Huang, W.; Yang, H.; Hu, Z.; Fan, Y.; Guan, X.; Feng, W.; Liu, Z.; Sun, Y., Rigidity Bridging Flexibility to Harmonize Three Excited-State Deactivation Pathways for NIR-II-Fluorescent-Imaging-Guided Phototherapy. *Advanced Healthcare Materials n/a* (n/a), 2101003.
61. Wang, Q.; Xu, J.; Geng, R.; Cai, J.; Li, J.; Xie, C.; Tang, W.; Shen, Q.; Huang, W.; Fan, Q., High performance one-for-all phototheranostics: NIR-II fluorescence imaging guided mitochondria-targeting phototherapy with a single-dose injection and 808 nm laser irradiation. *Biomaterials* **2020,** *231*, 119671.
62. Sun, P.; Chen, Y.; Sun, B.; Zhang, H.; Chen, K.; Miao, H.; Fan, Q.; Huang, W., Thienothiadiazole-Based NIR-II Dyes with D–A–D Structure for NIR-II Fluorescence Imaging Systems. *ACS Applied Bio Materials* **2021,** *4* (5), 4542-4548.
63. Zhang, R.; He, X.; Jiang, J.-M.; Li, P.-P.; Wang, H.-Y.; Li, L.; Yang, J.-X.; Kong, L., A computational and experimental investigation of donor-acceptor BODIPY based near-infrared fluorophore for in vivo imaging. *Bioorganic Chemistry* **2021,** *110*, 104789.

64. Min, X.; Zhang, J.; Li, R.-H.; Xia, F.; Cheng, S.-Q.; Li, M.; Zhu, W.; Zhou, W.; Li, F.; Sun, Y., Encapsulation of NIR-II AIEgens in Virus-like Particles for Bioimaging. *ACS Applied Materials & Interfaces* **2021,** *13* (15), 17372-17379.
65. Sun, Y.; Qu, C.; Chen, H.; He, M.; Tang, C.; Shou, K.; Hong, S.; Yang, M.; Jiang, Y.; Ding, B.; Xiao, Y.; Xing, L.; Hong, X.; Cheng, Z., Novel benzo-bis(1,2,5-thiadiazole) fluorophores for in vivo NIR-II imaging of cancer. *Chemical Science* **2016,** *7* (9), 6203-6207.
66. Zhang, L. e.; Liu, C.; Zhou, S.; Wang, R.; Fan, Q.; Liu, D.; Wu, W.; Jiang, X., Improving Quantum Yield of a NIR-II Dye by Phenylazo Group. *Advanced Healthcare Materials* **2020,** *9* (4), 1901470.
67. Li, S.; Chen, H.; Liu, H.; Liu, L.; Yuan, Y.; Mao, C.; Zhang, W.; Zhang, X.; Guo, W.; Lee, C.-S.; Liang, X.-J., In Vivo Real-Time Pharmaceutical Evaluations of Near-Infrared II Fluorescent Nanomedicine Bound Polyethylene Glycol Ligands for Tumor Photothermal Ablation. *ACS Nano* **2020,** *14* (10), 13681-13690.
68. Du, J.; Liu, S.; Zhang, P.; Liu, H.; Li, Y.; He, W.; Li, C.; Chau, J. H. C.; Kwok, R. T. K.; Lam, J. W. Y.; Cai, L.; Huang, Y.; Zhang, W.; Hou, J.; Tang, B. Z., Highly Stable and Bright NIR-II AIE Dots for Intraoperative Identification of Ureter. *ACS Applied Materials & Interfaces* **2020,** *12* (7), 8040-8049.
69. Li, Y.; Cai, Z.; Liu, S.; Zhang, H.; Wong, S. T. H.; Lam, J. W. Y.; Kwok, R. T. K.; Qian, J.; Tang, B. Z., Design of AIEgens for near-infrared IIb imaging through structural modulation at molecular and morphological levels. *Nature Communications* **2020,** *11* (1), 1255.
70. Liu, S.; Li, Y.; Zhang, J.; Zhang, H.; Wang, Y.; Chuah, C.; Tang, Y.; Lam, J. W. Y.; Kwok, R. T. K.; Ou, H.; Ding, D.; Tang, B. Z., A two-in-one Janus NIR-II AIEgen with balanced absorption and emission for image-guided precision surgery. *Materials Today Bio* **2021,** *10*, 100087.
71. Liu, S.; Chen, R.; Zhang, J.; Li, Y.; He, M.; Fan, X.; Zhang, H.; Lu, X.; Kwok, R. T. K.; Lin, H.; Lam, J. W. Y.; Qian, J.; Tang, B. Z., Incorporation of Planar Blocks into Twisted Skeletons: Boosting Brightness of Fluorophores for Bioimaging beyond 1500 Nanometer. *ACS Nano* **2020,** *14* (10), 14228-14239.
72. Sun, Y.; Ding, M.; Zeng, X.; Xiao, Y.; Wu, H.; Zhou, H.; Ding, B.; Qu, C.; Hou, W.; Er-bu, A. G. A.; Zhang, Y.; Cheng, Z.; Hong, X., Novel bright-emission small-molecule NIR-II fluorophores for in vivo tumor imaging and image-guided surgery. *Chemical Science* **2017,** *8* (5), 3489-3493.
73. Wan, H.; Yue, J.; Zhu, S.; Uno, T.; Zhang, X.; Yang, Q.; Yu, K.; Hong, G.; Wang, J.; Li, L.; Ma, Z.; Gao, H.; Zhong, Y.; Su, J.; Antaris, A. L.; Xia, Y.; Luo, J.; Liang, Y.; Dai, H., A bright organic NIR-II nanofluorophore for three-dimensional imaging into biological tissues. *Nature Communications* **2018,** *9* (1), 1171.
74. Qi, J.; Sun, C.; Zebibula, A.; Zhang, H.; Kwok, R. T. K.; Zhao, X.; Xi, W.; Lam, J. W. Y.; Qian, J.; Tang, B. Z., Real-Time and High-Resolution Bioimaging with Bright Aggregation-Induced Emission Dots in Short-Wave Infrared Region. *Advanced Materials* **2018,** *30* (12), 1706856.
75. Alifu, N.; Zebibula, A.; Qi, J.; Zhang, H.; Sun, C.; Yu, X.; Xue, D.; Lam, J. W. Y.; Li, G.; Qian, J.; Tang, B. Z., Single-Molecular Near-Infrared-II Theranostic Systems: Ultrastable Aggregation-Induced Emission Nanoparticles for Long-Term Tracing and Efficient Photothermal Therapy. *ACS Nano* **2018,** *12* (11), 11282-11293.
76. Lin, J.; Zeng, X.; Xiao, Y.; Tang, L.; Nong, J.; Liu, Y.; Zhou, H.; Ding, B.; Xu, F.; Tong, H.; Deng, Z.; Hong, X., Novel near-infrared II aggregation-induced emission dots for in vivo bioimaging. *Chemical Science* **2019,** *10* (4), 1219-1226.
77. Xu, P.; Kang, F.; Yang, W.; Zhang, M.; Dang, R.; Jiang, P.; Wang, J., Molecular engineering of a high quantum yield NIR-II molecular fluorophore with aggregation-induced emission (AIE) characteristics for in vivo imaging. *Nanoscale* **2020,** *12* (8), 5084-5090.
78. Li, Q.; Ding, Q.; Li, Y.; Zeng, X.; Liu, Y.; Lu, S.; Zhou, H.; Wang, X.; Wu, J.; Meng, X.; Deng, Z.; Xiao, Y., Novel small-molecule fluorophores for in vivo NIR-IIa and NIR-IIb imaging. *Chemical Communications* **2020,** *56* (22), 3289-3292.

79. Li, Y.; Liu, Y.; Li, Q.; Zeng, X.; Tian, T.; Zhou, W.; Cui, Y.; Wang, X.; Cheng, X.; Ding, Q.; Wang, X.; Wu, J.; Deng, H.; Li, Y.; Meng, X.; Deng, Z.; Hong, X.; Xiao, Y., Novel NIR-II organic fluorophores for bioimaging beyond 1550 nm. *Chemical Science* **2020,** *11* (10), 2621-2626.
80. Sheng, Z.; Guo, B.; Hu, D.; Xu, S.; Wu, W.; Liew, W. H.; Yao, K.; Jiang, J.; Liu, C.; Zheng, H.; Liu, B., Bright Aggregation-Induced-Emission Dots for Targeted Synergetic NIR-II Fluorescence and NIR-I Photoacoustic Imaging of Orthotopic Brain Tumors. *Advanced Materials* **2018,** *30* (29), 1800766.
81. Wu, W.; Yang, Y.; Yang, Y.; Yang, Y.; Zhang, K.; Guo, L.; Ge, H.; Chen, X.; Liu, J.; Feng, H., Molecular Engineering of an Organic NIR-II Fluorophore with Aggregation-Induced Emission Characteristics for In Vivo Imaging. *Small* **2019,** *15* (20), 1805549.
82. Fang, Y.; Shang, J.; Liu, D.; Shi, W.; Li, X.; Ma, H., Design, Synthesis, and Application of a Small Molecular NIR-II Fluorophore with Maximal Emission beyond 1200 nm. *Journal of the American Chemical Society* **2020,** *142* (36), 15271-15275.
83. Li, Y.; Zhang, J.; Liu, S.; Zhang, C.; Chuah, C.; Tang, Y.; Kwok, R. T. K.; Lam, J. W. Y.; Ou, H.; Ding, D.; Tang, B. Z., Enlarging the Reservoir: High Absorption Coefficient Dyes Enable Synergetic Near Infrared-II Fluorescence Imaging and Near Infrared-I Photothermal Therapy. *Advanced Functional Materials* **2021,** *31* (29), 2102213.
84. Wang, Q.; Xia, B.; Xu, J.; Niu, X.; Cai, J.; Shen, Q.; Wang, W.; Huang, W.; Fan, Q., Biocompatible small organic molecule phototheranostics for NIR-II fluorescence/photoacoustic imaging and simultaneous photodynamic/photothermal combination therapy. *Materials Chemistry Frontiers* **2019,** *3* (4), 650-655.
85. Chen, S.; Miao, H.; Jiang, X.; Sun, P.; Fan, Q.; Huang, W., Starlike polymer brush-based ultrasmall nanoparticles with simultaneously improved NIR-II fluorescence and blood circulation for efficient orthotopic glioblastoma imaging. *Biomaterials* **2021,** *275*, 120916.
86. He, K.; Chen, S.; Xu, W.; Tai, X.; Chen, Y.; Sun, P.; Fan, Q.; Huang, W., High-stability NIR-II fluorescence polymer synthesized by atom transfer radical polymerization for application in high-resolution NIR-II imaging. *Biomaterials Science* **2021**.
87. Piwoński, H.; Li, W.; Wang, Y.; Michinobu, T.; Habuchi, S., Improved Fluorescence and Brightness of Near-Infrared and Shortwave Infrared Emitting Polymer Dots for Bioimaging Applications. *ACS Applied Polymer Materials* **2020,** *2* (2), 569-577.
88. Tang, Y.; Li, Y.; Lu, X.; Hu, X.; Zhao, H.; Hu, W.; Lu, F.; Fan, Q.; Huang, W., Bio-Erasable Intermolecular Donor–Acceptor Interaction of Organic Semiconducting Nanoprobes for Activatable NIR-II Fluorescence Imaging. *Advanced Functional Materials* **2019,** *29* (10), 1807376.
89. Shou, K.; Tang, Y.; Chen, H.; Chen, S.; Zhang, L.; Zhang, A.; Fan, Q.; Yu, A.; Cheng, Z., Diketopyrrolopyrrole-based semiconducting polymer nanoparticles for in vivo second near-infrared window imaging and image-guided tumor surgery. *Chemical Science* **2018,** *9* (12), 3105-3110.
90. Liu, Y.; Liu, J.; Chen, D.; Wang, X.; Zhang, Z.; Yang, Y.; Jiang, L.; Qi, W.; Ye, Z.; He, S.; Liu, Q.; Xi, L.; Zou, Y.; Wu, C., Fluorination Enhances NIR-II Fluorescence of Polymer Dots for Quantitative Brain Tumor Imaging. *Angewandte Chemie International Edition* **2020,** *59* (47), 21049-21057.
91. Chen, D.; Liu, Y.; Zhang, Z.; Liu, Z.; Fang, X.; He, S.; Wu, C., NIR-II Fluorescence Imaging Reveals Bone Marrow Retention of Small Polymer Nanoparticles. *Nano Letters* **2021,** *21* (1), 798-805.
92. Hong, G.; Zou, Y.; Antaris, A. L.; Diao, S.; Wu, D.; Cheng, K.; Zhang, X.; Chen, C.; Liu, B.; He, Y.; Wu, J. Z.; Yuan, J.; Zhang, B.; Tao, Z.; Fukunaga, C.; Dai, H., Ultrafast fluorescence imaging in vivo with conjugated polymer fluorophores in the second near-infrared window. *Nature Communications* **2014,** *5* (1), 4206.
93. Zhang, W.; Huang, T.; Li, J.; Sun, P.; Wang, Y.; Shi, W.; Han, W.; Wang, W.; Fan, Q.; Huang, W., Facial Control Intramolecular Charge Transfer of Quinoid Conjugated Polymers for Efficient in Vivo NIR-II Imaging. *ACS Applied Materials & Interfaces* **2019,** *11* (18), 16311-16319.

94. Miao, Y.; Gu, C.; Yu, B.; Zhu, Y.; Zou, W.; Shen, Y.; Cong, H., Conjugated-Polymer-Based Nanoparticles with Efficient NIR-II Fluorescent, Photoacoustic and Photothermal Performance. *ChemBioChem* **2019,** *20* (21), 2793-2799.
95. Dai, Y.; Sun, Z.; Zhao, H.; Qi, D.; Li, X.; Gao, D.; Li, M.; Fan, Q.; Shen, Q.; Huang, W., NIR-II fluorescence imaging guided tumor-specific NIR-II photothermal therapy enhanced by starvation mediated thermal sensitization strategy. *Biomaterials* **2021,** *275*, 120935.
96. Zhu, Y.; Gu, C.; Miao, Y.; Yu, B.; Shen, Y.; Cong, H., D–A polymers for fluorescence/photoacoustic imaging and characterization of their photothermal properties. *Journal of Materials Chemistry B* **2019,** *7* (42), 6576-6584.
97. Guo, B.; Feng, Z.; Hu, D.; Xu, S.; Middha, E.; Pan, Y.; Liu, C.; Zheng, H.; Qian, J.; Sheng, Z.; Liu, B., Precise Deciphering of Brain Vasculatures and Microscopic Tumors with Dual NIR-II Fluorescence and Photoacoustic Imaging. *Advanced Materials* **2019,** *31* (30), 1902504.
98. Liu, Y.; Liu, J.; Chen, D.; Wang, X.; Liu, Z.; Liu, H.; Jiang, L.; Wu, C.; Zou, Y., Quinoxaline-Based Semiconducting Polymer Dots for in Vivo NIR-II Fluorescence Imaging. *Macromolecules* **2019,** *52* (15), 5735-5740.
99. Chen, Y.; Sun, B.; Jiang, X.; Yuan, Z.; Chen, S.; Sun, P.; Fan, Q.; Huang, W., Double-acceptor conjugated polymers for NIR-II fluorescence imaging and NIR-II photothermal therapy applications. *Journal of Materials Chemistry B* **2021,** *9* (4), 1002-1008.
100. Yang, Y.; Fan, X.; Li, L.; Yang, Y.; Nuernisha, A.; Xue, D.; He, C.; Qian, J.; Hu, Q.; Chen, H.; Liu, J.; Huang, W., Semiconducting Polymer Nanoparticles as Theranostic System for Near-Infrared-II Fluorescence Imaging and Photothermal Therapy under Safe Laser Fluence. *ACS Nano* **2020,** *14* (2), 2509-2521.
101. Zhang, Z.; Fang, X.; Liu, Z.; Liu, H.; Chen, D.; He, S.; Zheng, J.; Yang, B.; Qin, W.; Zhang, X.; Wu, C., Semiconducting Polymer Dots with Dual-Enhanced NIR-IIa Fluorescence for Through-Skull Mouse-Brain Imaging. *Angewandte Chemie International Edition* **2020,** *59* (9), 3691-3698.
102. Liu, S.; Ou, H.; Li, Y.; Zhang, H.; Liu, J.; Lu, X.; Kwok, R. T. K.; Lam, J. W. Y.; Ding, D.; Tang, B. Z., Planar and Twisted Molecular Structure Leads to the High Brightness of Semiconducting Polymer Nanoparticles for NIR-IIa Fluorescence Imaging. *Journal of the American Chemical Society* **2020,** *142* (35), 15146-15156.
103. Deng, G.; Peng, X.; Sun, Z.; Zheng, W.; Yu, J.; Du, L.; Chen, H.; Gong, P.; Zhang, P.; Cai, L.; Tang, B. Z., Natural-Killer-Cell-Inspired Nanorobots with Aggregation-Induced Emission Characteristics for Near-Infrared-II Fluorescence-Guided Glioma Theranostics. *ACS Nano* **2020,** *14* (9), 11452-11462.
104. Liu, M.-H.; Chen, T.-C.; Vicente, J. R.; Yao, C.-N.; Yang, Y.-C.; Chen, C.-P.; Lin, P.-W.; Ho, Y.-C.; Chen, J.; Lin, S.-Y.; Chan, Y.-H., Cyanine-Based Polymer Dots with Long-Wavelength Excitation and Near-Infrared Fluorescence beyond 900 nm for In Vivo Biological Imaging. *ACS Applied Bio Materials* **2020,** *3* (6), 3846-3858.
105. Liu, M.-H.; Zhang, Z.; Yang, Y.-C.; Chan, Y.-H., Polymethine-Based Semiconducting Polymer Dots with Narrow-Band Emission and Absorption/Emission Maxima at NIR-II for Bioimaging. *Angewandte Chemie International Edition* **2021,** *60* (2), 983-989.
